# Cationic Triarylchlorostibonium Lewis Acids

**DOI:** 10.1021/acs.organomet.2c00426

**Published:** 2023-02-20

**Authors:** Omar Coughlin, Tobias Krämer, Sophie L. Benjamin

**Affiliations:** † Department of Chemistry, Nottingham Trent University, Clifton Lane, Nottingham NG11 8NS, U.K.; ‡ Department of Chemistry, Maynooth University, Maynooth, Co. Kildare W23 F2H6, Ireland

## Abstract

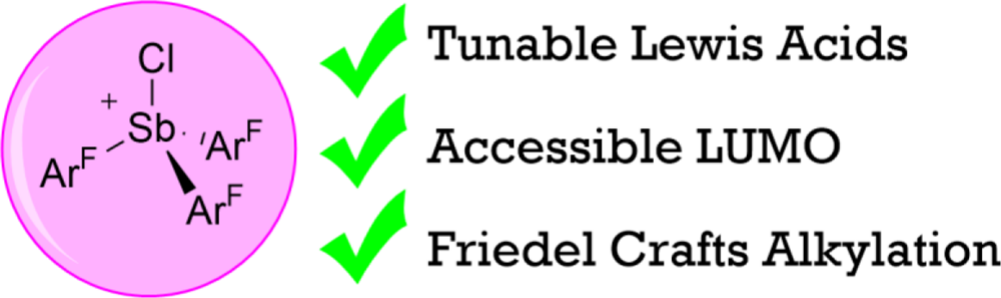

Organopnictogen cations show promise as powerful, tunable main-group Lewis acid catalysts. The synthesis, solid-state structures, and reactivity of a series of weakly coordinated triarylchlorostibonium salts [Ar_3_SbCl][B(C_6_F_5_)_4_] (Ar = Ph, 3-FC_6_H_4_, 4-FC_6_H_4_, 3,5-F_2_C_6_H_3_, 2,4,6-F_3_C_6_H_2_) are reported. The cation in each adopts a tetrahedral coordination environment of antimony, with near complete separation from the anion. Structural, computational, and reactivity studies reveal that the Lewis acidity of [Ar_3_SbCl]^+^ generally increases with increased fluorination of the Ar substituents, with a secondary quenching effect from *para* fluorination. [Ar_3_SbCl]^+^ is reduced to Ar_3_Sb in the presence of Et_3_SiH, and the mechanism of this reaction has been modeled computationally. Preliminary studies demonstrate that they are useful catalysts for the dimerization of 1,1-diphenylethylene and the Friedel–Crafts alkylation of benzene.

## Introduction

**Figure 1:**
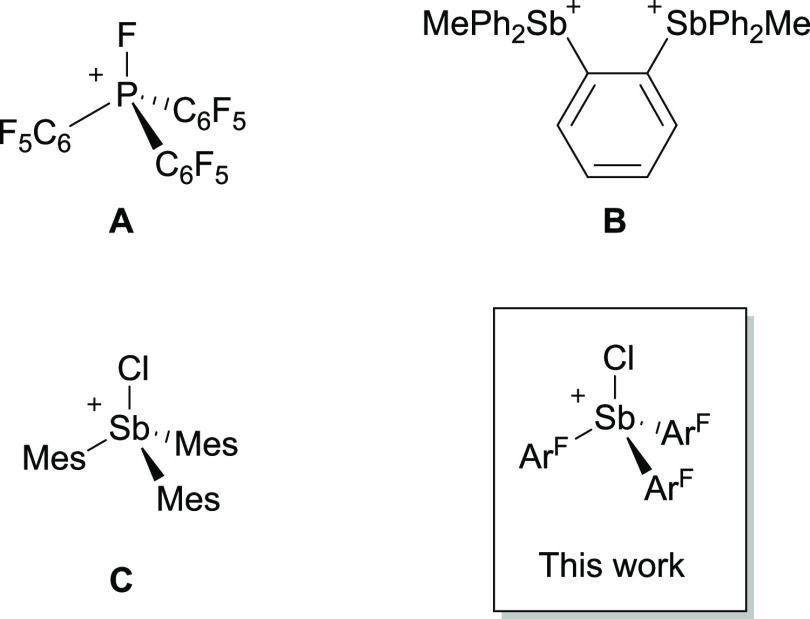
Weakly coordinated cationic Pn(V) catalysts.

Organopnictogen derivatives are gaining increasing attention due to their catalytic potential, including in previous transition metal-dominated redox catalysis and as a new generation of tunable Lewis acid catalysts.^1−5^ Electrophilic group 15 cations in particular are versatile Lewis acids, with potential applications in catalysis and anion sensing. Fluorophosphonium salts such as [(C_6_F_5_)_3_PF][B(C_6_F_5_)_4_] (**A**, Figure 1) and its derivatives have been shown to catalyze a diverse range of organic transformations, including C–X activation and C–C bond forming reactions.^6−12^ These compounds benefit from possessing a well-defined site of Lewis acidity, namely, the low-lying σ*_P–F_ lowest unoccupied molecular orbital (LUMO), *trans* to the fluoride substituent, the accessibility of which relies on the use of a bulky, non-coordinating anion. Related Pn(V) cations of the heavier pnictogens (Pn = As, Sb, and Bi) are attractive targets as the increased electropositivity of these elements compared to P offers the possibility of considerably increased Lewis acidity, as well as potentially divergent reactivity compared with P congeners. A small number of organostibonium cations have been investigated for their catalytic reactivity, including [(C_6_F_5_)_4_Sb][B(C_6_F_5_)_4_], which promotes the hydrodefluorination of compounds containing strong C^sp3^–F bonds in the presence of Et_3_SiH.^13^ Dicationic bis-stibonium ions (**B** for example, Figure 1) and phosphine-supported stibonium derivatives have also been shown to activate aldehydes and catalyze the transfer hydrogenation of quinolines.^14−17^ Very few halide-substituted stibonium cations have been reported.^18−21^ Of these, [Mes_3_SbCl][SbCl_6_] (**C**, Figure 1) is the only example not supported by cation–anion interactions in the solid state; the structure of [Ph_3_SbCl][SbCl_6_] contains Sb···ClSbCl_5_ contacts (3.231(6) Å).^18,19^ Both of these chlorostibonium salts slowly promote the polymerization of tetrahydrofuran (THF); however, while [Ph_3_SbCl][SbCl_6_] efficiently catalyzes the dimerization of 1,1-diphenylethylene (DPE) (Scheme 5b), **C** is inactive in this reaction, attributed to the lower electron deficiency of the Sb center.^18^ We reasoned that weakly coordinated cations of the form [R_3_SbX]^+^ (X = halide) would have the threefold advantages of a highly Lewis acidic cationic Sb(V) center, a well-defined Lewis acidic site *trans* to the halide substituent, and the potential to modulate reactivity by varying the organic substituents, making them ideal targets in the design of tunable Lewis acid catalysts.

Here, we report the synthesis of a series of triarylchlorostibonium salts featuring near-tetrahedral [Ar^F^_3_SbCl]^+^ cations with different degrees of fluorination at the aryl substituents and an investigation of their Lewis acidity and reactivity using both computational and experimental methods.

## Results and Discussion

**Scheme 1:**
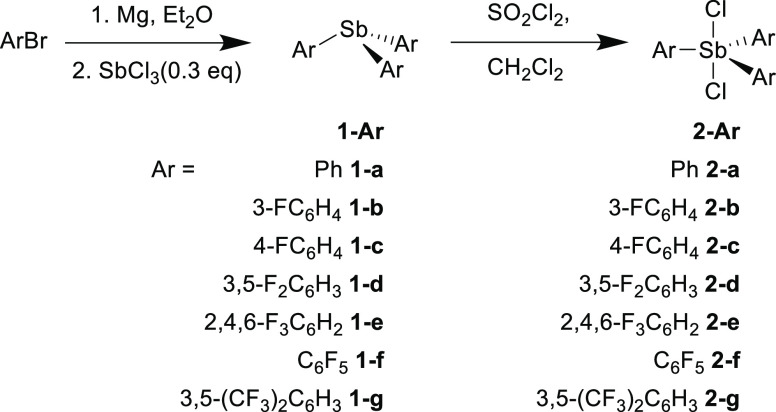
Synthesis of Triarylstibines (**1-Ar**) and Triarylstibine Dihalides (**2-Ar**)

**Scheme 2:**
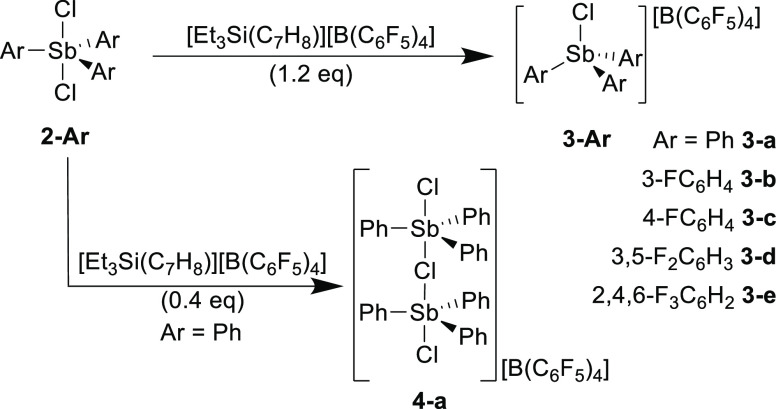
Synthesis of Triarylchlorostibonium Tetrakis(pentafluorophenyl)borates **3-Ar** and **4-a** Conditions: toluene, room temperature.

In order to isolate cations of the form [Ar_3_SbCl]^+^ with an accessible acidic site, minimizing the formation of cation–anion interactions, salts of the form [Ar_3_SbCl][B(C_6_F_5_)_4_] were targeted due to the weakly coordinating and chemically inert nature of the borate anion.^22^ The triarylstibine dichlorides Ar_3_SbCl_2_ (**2-Ar**) were synthesized by oxidative chlorination of the arylstibines **1-Ar** (Scheme 1). Adding a solution of **2-a** to a suspension of a slight excess of [(Et_3_Si)(C_7_H_8_)][B(C_6_F_5_)_4_] in toluene, followed by recrystallization, yielded analytically pure [Ph_3_SbCl][B(C_6_F_5_)] (**3-a**) (Scheme 2). The synthesis of four other triarylchlorostibonium salts **3-b** to **3-e** was carried out in an equivalent manner. Similar treatment of **2-f** and **2-g** resulted in multiple products, predominantly unreacted triarylstibine dichlorides. It appears that in these cases, the presence of highly electron withdrawing fluorinated substituents makes chloride abstraction by the silyl cation unfavorable.

**Figure 2:**
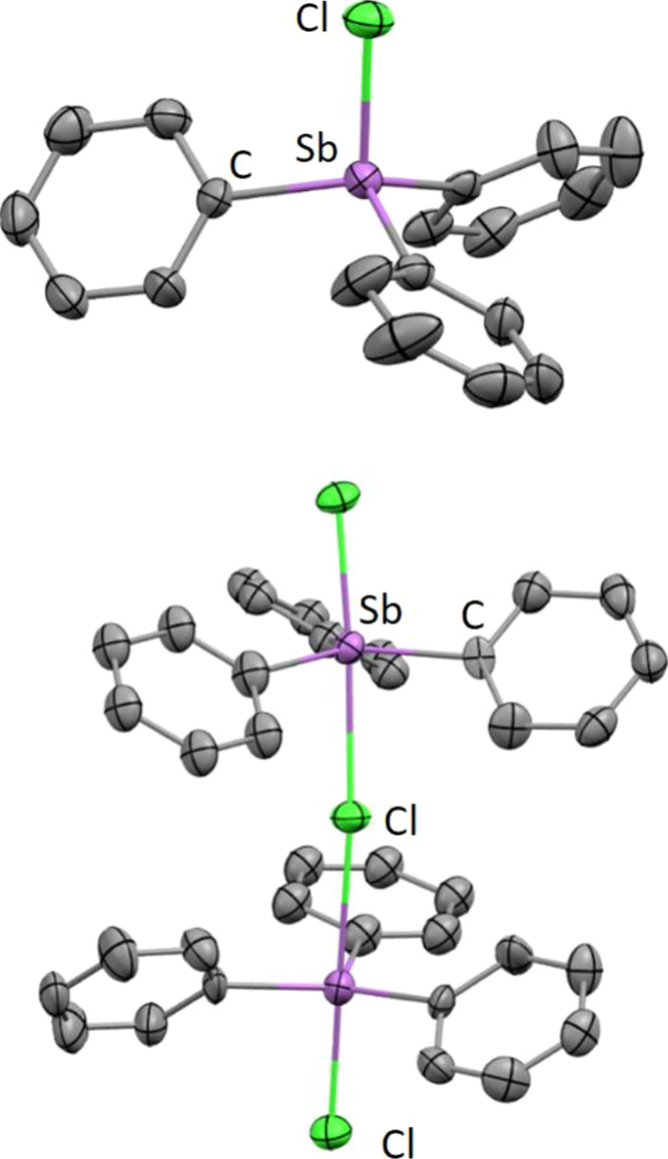
Solid-state structure of the cations in **3-a** (top) and **4-a** (bottom). Ellipsoids are shown at 50% probability, and hydrogen atoms have been omitted for clarity. One phenyl group in **3-a** is disordered over two positions, and only one is shown here.

The ^1^H, ^13^C{^1^H}, and ^19^F NMR spectra of **3-a** to **3-e** showed signals in the expected regions for the cations, with only small changes in the chemical shift compared to the respective dichlorides **2-Ar** (see Electronic Supplementary Information, ESI). In all cases, the *ipso* C could not be identified in the ^13^C{^1^H} NMR spectrum due to broadening from coupling with quadrupolar Sb nuclei. The presence of the anion was confirmed by the characteristic ^19^F resonance at −133.7 ppm, though anion resonances in the ^13^C{^1^H} spectra were weak and broad, presumably due to extensive coupling with ^19^F and ^11^B, and only the sharpest *para*-F resonances have been assigned as these are characteristic. The solid-state structures of **3-Ar** were determined by X-ray crystallography (Figures 2 and S6–S9). Key structural parameters for the series are summarized in Table 1. In all cases, the cation adopts a distorted tetrahedral geometry, with mean Cl–Sb–C angles ranging between 105.1° and 108.1°, in marked contrast to 98.3° in the near-trigonal bipyramidal [Ph_3_SbCl][SbCl_6_]. The borate counterions are very weakly coordinating, with between two and four long Sb···F–B contacts that vary significantly in length and angle of approach within the **3-Ar** series, including between the two independent cations within the structure of **3-d** (Figure S14),^23^ suggesting that the dominant factor dictating these contacts is packing effects rather than any directional interaction. The minimum Sb···F–B distance in **3-a** is 3.80 Å, considerably longer than the Sb···Cl–Sb distance of 3.20 Å in the [SbCl_6_] salt of this anion despite the smaller radius of F. However, some Sb···F–B contacts remain within the sum of the van der Waals radii of Sb and F (3.93 Å), meaning that while the cations in **3-Ar** cannot be described as fully uncoordinated, they can be considered to be very weakly coordinated, with a vacant acidic site *trans* to Sb–Cl, which is not significantly blocked by anion interactions. Across the series, the length of the covalent Sb–Cl bond decreases slightly with increased fluorination of the aryl substituents as would be expected for an increasingly electron deficient Sb center (with the exception of **3-b**, in which the bond length is surprisingly slightly longer than that in **3-a**), and are generally around 0.2 Å shorter than those in the parent stibine dihalides **2-Ar**.^24^

**Table 1:** Selected Data for the **3-Ar** Series of Compounds

compound	Sb–Cl (Å)	minimum Sb···FB distance (Å)	average Sb–C (Å)	average Sb–Cl in parent **2-Ar** (Å)	average ∠Cl–Sb–C (°)	τ4	Sb–B (Å)	FIA (kJ/mol)	LUMO (eV)
**3-a**	2.2821(14)	3.80	2.086	2.463	105.93	0.90	6.344	566	–4.64
**3-b**	2.2924(11)	3.61	2.086		107.53	0.92	7.363	595	–4.97
**3-c**	2.2757(9)	3.44	2.083		105.10	0.96	7.795	591	–4.82
**3-d**	2.2604(19), 2.2608(16)	3.36	2.091	2.455	108.12	0.96	7.484	623	–5.28
**3-e**	2.2532(19)	3.67	2.073	2.415	107.2	0.97	7.297	597	–5.15

During early attempts to synthesize **3-a** in which **2-a** was used in slight excess, small amounts of a dimeric monocation of the form [(Ph_3_SbCl)_2_(μ-Cl)][B(C_6_F_5_)_4_] (**4-a**) were also isolated and characterized crystallographically (Figure 2). By using an appropriate reaction stoichiometry, **4-a** was directly targeted and isolated in 62% yield (Scheme 2), though elemental analysis suggests some **3-a** may be present as a minor product, potentially exchanging in solution with **4-a** on the spectroscopic timescale, resulting in only one set of NMR resonances.

Similar dinuclear fluorobismuthonium cations were recently reported, along with mononuclear and trinuclear examples, though in their case, aggregation appears to be entirely controlled by steric factors rather than the precursor ratio.^25^ A fluoride-bridged tetraarylstibonium dimer with a related structure was also reported recently.^26^ The structural parameters of the two trigonal bipyramidal Sb centers in **4-a** are similar, with terminal Sb–Cl bond lengths (mean 2.380 Å) shorter than those in the neutral **2-a** (2.481 Å) but longer than those in the monomeric **3-a** cation (2.282 Å), indicating an intermediate positive charge at each Sb. A small amount of the tetraarylstibonium salt [(4-FC_6_H_4_)_4_Sb][B(C_6_F_5_)_4_] was also isolated from a solution of **3-c** after standing for several days and was crystallographically characterized (Figure S11).

We proceeded to investigate the Lewis acidity of the **3-Ar** series both computationally and experimentally. Fluoride ion affinity (FIA) is one measure that can be used to compare the Lewis acidities of a series of compounds.^27^ FIAs were calculated for the cationic fragments of the salts **3-Ar** (**Ar = a–g**) in dichloromethane (DCM) and are included in Tables 1 and S2. While the figures obtained suggest significant Lewis acidity in all cases, it is unsuitable to quantitively compare them with FIAs reported for other Lewis acids, which are calculated using different levels of theory. However, within the **3-Ar** series, some general trends can be identified. Increasing fluorination of the aryl substituents leads to an increased FIA, with fluoride in the *meta* position having a stronger effect than fluoride in the *para* position, in line with their respective Hammett parameters;^28^ hence, **3c** and **3e** have lower predicted FIAs than **3-b** or **3-d**. As expected, FIAs for the unobtainable **3-f** and **3-g** are the highest in the series, though they remain lower than that of [Et_3_Si(tol)]^+^. However, it is notable that the LUMO energies of these two cations are the only ones significantly more negative than [Et_3_Si(tol)]^+^, perhaps providing an explanation for the failure to synthesize them from [Et_3_Si(tol)][B(C_6_F_5_)_4_] (Table S2).

**Figure 3:**
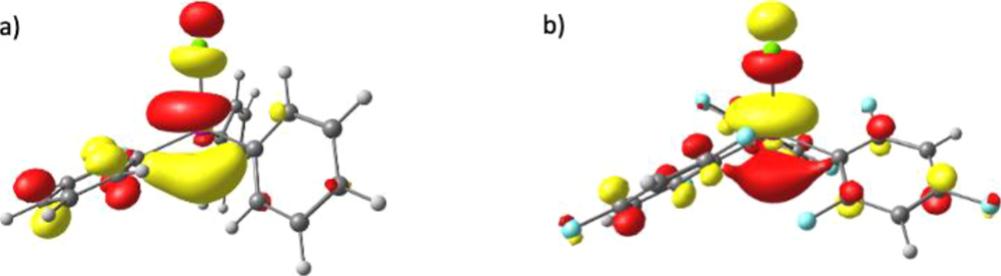
DFT-calculated Kohn–Sham molecular orbitals (isovalue 0.05) representing (a) LUMO of **3-a** and (b) LUMO of **3-e**.

A close examination of the Kohn–Sham orbitals of [Ar_3_SbCl]^+^ (Figure 3 and ESI) demonstrates that in all cases, the LUMO has a predominantly σ*_Sb–Cl_ character, similar to those previously computed for cations of this type^18^ and reminiscent of the σ*_P–F_ orbital that has been shown to act as the Lewis acidic site in related fluorophosphonium species.^6^ The large component *trans* to the Sb–Cl bond gives a likely angle of approach for Lewis base interactions.

**Scheme 3:**
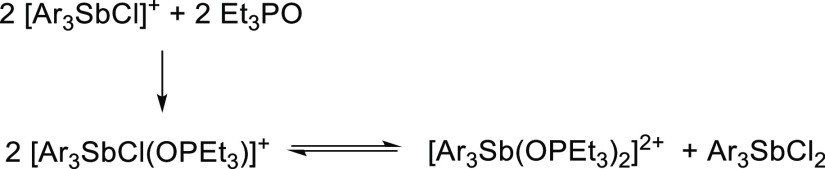
Proposed Reactivity of **3-Ar** with Et_3_PO

The Gutmann–Beckett method is a semi-quantitative measure of effective Lewis acidity based on the change in the ^31^P NMR shift of Et_3_PO in the presence of a Lewis acid.^29^ This method assumes simple adduct formation; however, the reactivity of **3-Ar** in the presence of Et_3_PO appears more complicated, with multiple peaks observed in the ^31^P NMR spectrum in some cases. Crystals obtained from a mixture of **3-e** and Et_3_PO in CD_2_Cl_2_ were determined to be highly disordered **2-e** co-crystallized with a molecule of Et_3_PO (Figure S12). This reactivity is reminiscent of **A** (Figure 1), which forms the respective phosphine difluoride (C_6_F_5_)_3_PF_2_ on mixing with dimethylformamide.^6^ We hypothesize that the source of Cl is another molecule of **3-e** and that a Schlenk-like equilibrium may exist (Scheme 3).

While it was not possible to isolate the [B(C_6_F_5_)_4_]^−^ salts of the postulated [Ar_3_Sb(OPEt_3_)_2_]^2+^ dication in the solid state, equivalent treatment of the known compound [Ph_3_SbCl(OTf)]^30^ with Et_3_PO generated a mixture from which a few clear, colorless crystals of [Ph_3_Sb(OPEt_3_)_2_][OTf]_2_ were obtained (Figure S13). While X-ray structural data are of poor quality, the previously reported trigonal bipyramidal dication^31^ can be unambiguously identified, giving further weight to this interpretation.

**Figure 4:**
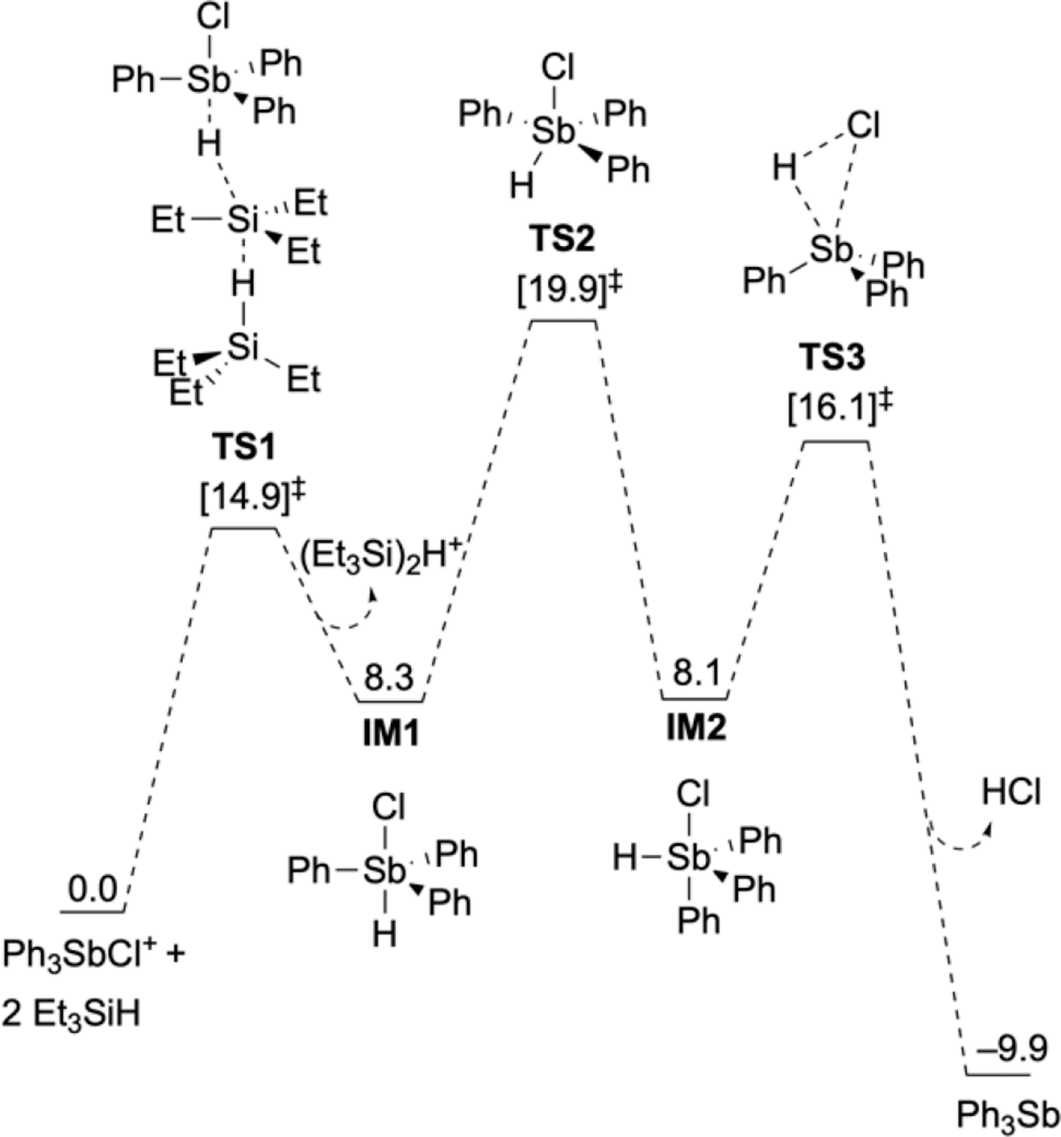
Calculated reaction profile for the reduction of Ph_3_SbCl^+^ by Et_3_SiH. Gibbs free energies (in kcal mol^–1^) relative to starting materials Ph_3_SbCl^+^ and 2 Et_3_SiH. All energies are calculated at the M06-2X(D3)/def2-QZVPP//M06-2X(D3)/def2-SVP level of theory corrected for the CH_2_Cl_2_ solvent.

Initial attempts to synthesize **3-Ar** employed [(Et_3_Si)_2_(μ-H)][B(C_6_F_5_)_4_] prepared in situ from trityl borate and neat Et_3_SiH.^32^ These reactions were not high yielding, and nuclear magnetic resonance (NMR) spectroscopy suggested that some reduction to the parent stibine (**1-Ar**) had occurred. This was overcome by the use of purified [(Et_3_Si)(C_7_H_8_)][B(C_6_F_5_)_4_],^33^ prepared by recrystallizing [(Et_3_Si)_2_(μ-H)][B(C_6_F_5_)_4_]^34,35^ in toluene. We suspected that the generation of **1-Ar** in the first instance could be attributed to the reduction of the stibonium product by residual Et_3_SiH. Direct reaction of excess Et_3_SiH with isolated **3-a** and **4-a** resulted in stoichiometric conversion to the parent stibine **1-a**. We undertook density functional theory (DFT) calculations to elucidate the mechanism of this reduction. The most energetically accessible pathway involves nucleophilic attack on the Sb center by a hydride, yielding a *trans-*Ar_3_SbClH intermediate (**IM1**) that isomerizes to *cis-*Ar_3_SbClH (**IM2**) followed by reductive elimination of HCl (Figure 4). Other routes involving elimination of benzene or chlorobenzene or initial chloride abstraction from **IM2** were modeled and found to be kinetically inaccessible (Figures S66 and S67). A number of fluorine-containing impurities in addition to Et_3_SiF were identified by NMR, possibly the result of decomposition of the borate anion by a transient uncoordinated Et_3_Si^+^ cation. This reactivity is not unprecedented; [(C_6_F_5_)_4_Sb][B(C_6_F_5_)_4_] also reduces to the parent stibine in the presence of Et_3_SiH,^13^ and the reduction of [(C_6_F_5_)_3_PF]^+^ (**A**, Figure 1) occurs via a similar mechanism.^36^ This reactivity precludes the use of these salts as direct catalysts for reactions involving silanes, such as the hydrodefluorination of halocarbons.

One potentially powerful feature of group 15 cations is the ability to activate C–X bonds. The stibonium salts **3-a** and **3-e** and the dimer **4-a** rapidly react with trityl chloride or trityl fluoride (Ph_3_CCl/Ph_3_CF) in CD_2_Cl_2_ to form Ar_3_SbCl_2_/Ar_3_SbClF, respectively, demonstrating the halophilicity of these cations. The stability of the resulting trityl cation assists the reaction thermodynamically.

**Scheme 4:**
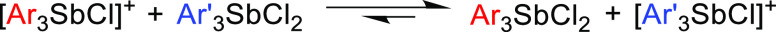
Chloride Exchange between **3-Ar** and **2-Ar′**

To probe the relative chlorophilicity of the triarylstibonium salts, competition reactions were performed in which **3-Ar** were mixed with **2-Ar′** (Ar ≠ Ar′). Chloride exchange between these two species generates **2-Ar** and **3-Ar′** (Scheme 4), the equilibrium favoring the formation of the dichloride with the more electron withdrawing aryl substituents and the stibonium cation with the less electron withdrawing aryl substituents. Mixing an equimolar solution of **3-b** and **2-d** in CD_2_Cl_2_ led to the complete formation of **2-b** and **3-d** by NMR. Mixing a solution of **3-c** and **2-a** under the same conditions resulted in the complete formation of **2-c** and **3-a**. Both results are consistent with the relative Lewis acidities inferred from the calculated FIA values for **3-Ar**.

**Scheme 5:**
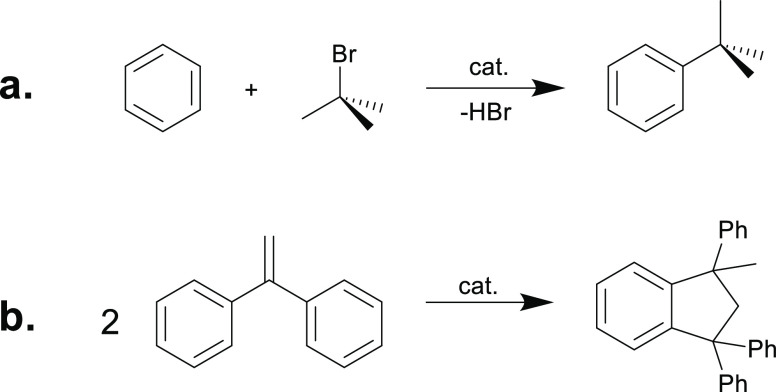
(a) Friedel–Crafts Alkylation of Benzene; (b) Friedel–Crafts Dimerization of DPE

To assess the catalytic potential of **3-Ar** for C–X bond activation, we chose the Friedel–Crafts alkylation of benzene as a test reaction (Scheme 5a). ^*t*^BuBr was selected as an alkylating agent as we reasoned that the Sb–Br bond in the putative Ar_3_SbClBr intermediate would be weak enough to eliminate HBr on reacting with the Wheland intermediate, and the bulky ^*t*^Bu would impede any carbocation rearrangements. A series of **3-Ar** with increasing fluorination of the aryl substituents were tested and are moderate catalysts in this reaction (Table 2); interestingly, **3-c** gives the highest yield despite crystallographic and computational data suggesting that it is of intermediate Lewis acidity. This may be due to requiring a balance between the rate of initial C–Br activation by the **3-Ar** cation and that of Sb–Br cleavage from the resulting **2-Ar** intermediate; however, more detailed mechanistic studies would be required to confirm this. No decomposition of the catalyst was observed in any of these reactions.

**Table 2:** Friedel–Crafts Alkylation of Benzene by ^*t*^BuBr Catalyzed by **3-Ar**^a^

catalyst	aryl group	loading (%)	conversion (%)
**3-a**	Ph	1	8
**3-a**	Ph	5	37
**3-c**	4-FC_6_H_4_	1	81
**3-e**	2,4,6-F_3_C_6_H_2_	1	12

a Conversion based on ^1^H NMR yield after 30 min in CDCl_3_.

The stibonium cations **3-Ar** also catalyze the dimerization of DPE, a common test reaction for Lewis acid catalysis (Scheme 5b). The reaction was complete after 2 h at room temperature in CD_2_Cl_2_ with 5% catalyst loading. We also tested **4-a**, which only gave traces of the product under the same conditions, suggesting that the dimeric structure is largely retained in solution, leaving no vacant catalytic site.

## Conclusions

In conclusion, we have reported the synthesis of a family of distorted tetrahedral chlorostibonium ions with electron withdrawing aryl substituents. Both experimental and computational investigations of their reactivity demonstrate that they are halophilic Lewis acids with well-defined LUMO sites that are accessible due to very weakly coordinating borate anions. Acidity was seen to increase with increasing fluorination of the aryl substituents, with a slight quenching effect from *para* fluorides. The chlorostibonium ions are reduced in the presence of Et_3_SiH, this reduction being proceeded through Sb–H containing intermediates. We have conducted the first investigation into the catalytic potential of these types of cations for C–C bond-forming Friedel–Crafts alkylation reactions, demonstrating that the catalyst efficiency is highly dependent on the aryl substituent.

## Experimental Methods

### General Considerations

Caution: All antimony-containing compounds should be treated as toxic. All manipulations were performed under an atmosphere of dry N_2_ using standard Schlenk or glovebox (Mbraun Unilab 2000) techniques unless otherwise stated. All glassware was dried in an oven at 150 °C and cooled under vacuum before use. THF, DCM, toluene, and *n*-hexane were dried using an Mbraun MB SPS5. All deuterated solvents were dried and stored over 4 Å molecular sieves. SbCl_3_ was sublimed in vacuo at 40–65 °C before use. Triethylsilane was distilled over CaH_2_, degassed by freeze/pump/thaw, and stored over 4 Å molecular sieves. All other reagents were used as received unless otherwise stated. [Et_3_Si(C_7_H_8_)][B(C_6_F_5_)_4_] was synthesized according to literature methods^32,37,38^ and recrystallized in toluene at −10 °C or by the addition of Et_3_SiH to a solution of [Ph_3_C][B(C_6_F_5_)_4_] in toluene.^35^

Synthetic details for the preparation of **1-Ar** and **2-Ar** are given in the Supplementary Information.

#### **3-a** [Ph_3_SbCl][B(C_6_F_5_)_4_]

To a stirring solution of [(Et_3_Si)C_7_H_8_][B(C_6_F_5_)_4_] (0.416 g, 0.47 mmol) in toluene (30 mL) was added a solution of Ph_3_SbCl_2_ (0.165 g, 0.39 mmol) in toluene (15 mL) at room temperature. An off-white oily suspension formed instantly. This was stirred at room temperature for 90 min. The oil was allowed to settle, and the solution was decanted. The oil was dried in vacuo to an oily solid, which was dissolved in CH_2_Cl_2_ and layered with hexane to give colorless crystals suitable for X-ray diffraction (XRD), which were isolated by filtration (0.065 g, 0.06 mmol, 15%). ^1^H NMR (400 MHz, CD_3_CN) δ ppm 7.67–7.87 (m, 9H, o/p-H) 8.06 (br d, *J* = 7.78 Hz, 6H, m-H). ^13^C{^1^H} NMR (101 MHz, CD_3_CN) δ ppm 131.07 (s) 134.0 (s) 134.2 (s), 148.1 (d, *J* = 238 Hz). ^19^F NMR (376 MHz, CD_3_CN) δ ppm −168.25 (br d, *J* = 14.45 Hz, B-C_6_F_5_) −163.81 (br d, *J* = 20.23 Hz, B-C_6_F_5_) −133.66 (br s, B-C_6_F_5_). Elemental analysis, found (calcd for C_42_H_15_BClF_20_Sb): H: 1.49% (1.42%) C: 47.15% (47.25%).

#### **3-b** [(3-FC_6_H_4_)_3_SbCl][B(C_6_F_5_)_4_]

To a stirring solution of [(Et_3_Si)C_7_H_8_][B(C_6_F_5_)_4_] (0.416 g, 0.47 mmol) in toluene (30 mL) was added a solution of (3-FC_6_H_4_)_3_SbCl_2_ (0.186 g, 0.39 mmol) in toluene (15 mL) at room temperature. A red oil formed instantly, and the reaction mixture was stirred at room temperature for 90 min. The oil was allowed to settle, and the solution was decanted. The oil was dried under vacuum and triturated with hexane to give a white waxy solid, which was dissolved in CH_2_Cl_2_ (5 mL) and layered with hexane (30 mL) to give colorless crystals suitable for XRD, which were isolated by filtration (0.120 g, 0.1 mmol, 25%). ^1^H NMR (400 MHz, CD_3_CN): 7.53–7.62 (m, 1H) 7.79 (td, *J* = 8.23, 5.49 Hz, 1H) 7.86–7.93 (m, 2H). ^13^C NMR (101 MHz, CD_3_CN): 120.9 (d, *J* = 25 Hz), 121.0 (d, *J* = 21 Hz) 130.0 (d, *J* = 4 Hz) 132.5 (d, *J* = 8 Hz) 133.9 (d, *J* = 8 Hz) 147.7 (br d, *J* = 242 Hz) 162.8 (d, *J* = 252 Hz). ^19^F NMR (376 MHz, CD_3_CN): −168.23 (br t, *J* = 17.34 Hz, B-C_6_F_5_) −163.80 (br t, *J* = 19 Hz, B-C_6_F_5_) −133.67 (br s, B-C_6_F_5_) −108.90 (br s, m-F). ^11^B NMR (128 MHz, CD_3_CN): −17.73 (s). Elemental analysis, found (calcd for C_42_H_11_BClF_23_Sb): H: 1.15% (1.08%) C: 45.12% (44.98%).

#### **3-c** [(4-FC_6_H_4_)_3_SbCl][B(C_6_F_5_)_4_]

To a stirring solution of [(Et_3_Si)C_7_H_8_][B(C_6_F_5_)_4_] (0.354 g, 0.40 mmol) in toluene (30 mL) was added a solution of (4-FC_6_H_4_)_3_SbCl_2_ (0.162 g, 0.34 mmol) in toluene (15 mL) at room temperature. A red oil formed instantly, and the reaction mixture was stirred at room temperature for 90 min. The oil was allowed to settle, and the solution was decanted. The oil was dried under vacuum to give a white foam, which was dissolved in CH_2_Cl_2_ (10 mL), giving a dark solution. The solution was filtered through celite and layered with hexane to give clear colorless crystals suitable for XRD, which were isolated by filtration (0.056 g, 0.05 mmol, 15%). ^1^H NMR (400 MHz, CD_3_CN) δ ppm 7.49–7.54 (t, *J* = 8.66 Hz, 6H, m-H) 8.08–8.12 (m, 6H, o-H). ^13^C{^1^H} NMR (101 MHz, CD_3_CN) δ ppm 118.4 (d, *J* = 22 Hz), 137.1 (d, *J* = 10 Hz) 165.9 (d, *J* = 255). ^19^F NMR (376 MHz, CD_3_CN) δ ppm −168.27 (br t, *J* = 15.89 Hz, B-C_6_F_5_) −163.83 (br t, *J* = 18.79 Hz-133.71 br s, B-C_6_F_5_) −104.63 (br s,p-F). Elemental analysis, found (calcd for C_42_H_11_BClF_23_Sb): H: 1.01% (1.08%) C: 44.45% (44.98%). A few colorless crystals of [(4-FC_6_H_4_)_4_Sb][B(C_6_F_5_)_4_] were isolated on standing of the mother liquor.

#### **3-d** [(3,5-F_2_C_6_H_3_)_3_SbCl][B(C_6_F_5_)_4_]

Synthesized as per **3-c**. Yielded off-white crystals (0.186 g, 0.16 mmol, 34%). ^1^H NMR (400 MHz, CD_3_CN): 7.44 (br t, *J* = 8.80 Hz, 3H, o-H) 7.77 (br s, 6H, p-H). ^13^C{^1^H} NMR (101 MHz, CD_3_CN): 110.2 (t, *J* = 25 Hz) 117.7 (m) 148.1 (d, *J* = 236 Hz) 138.3 (br d, *J* = 46 Hz) 163.5 (dd, *J*_1_ = 255 Hz, *J*_2_ = 12 Hz). ^19^F NMR (376 MHz, CD_3_CN): −168.28 (s, B-C_6_F_5_) −163.84 (br t, *J* = 20.23 Hz, B-C_6_F_5_) −133.72 (s, B-C_6_F_5_) −105.80 (s, m-F). Elemental analysis, found (calcd for C_42_H_9_BClF_26_Sb): H: 0.72% (0.77%) C: 42.75% (42.91%).

#### **3-e** [(2,4,6-F_3_C_6_H_2_)_3_SbCl][B(C_6_F_5_)_4_]

A solution of [(Et_3_Si)_2_H][B(C_6_F_5_)_4_](0.132 g, 0.15 mol) in toluene was added dropwise to a solution of (2,4,6-F_3_C_6_H_2_)_3_SbCl_2_ (0.0879 g, 0.15 mmol) in toluene (2 mL), which resulted in the formation of a red-orange oil, which was stirred for 1 h. The solvent was removed in vacuo to afford a red oil, which was washed with hexane. The oil was dissolved in CH_2_Cl_2_ (∼10 mL) and layered with hexane (∼15 mL) to afford green clear crystals suitable for XRD, which were isolated by filtration (0.078 g, 0.06 mmol, 40%). ^1^H NMR (400 MHz, CDCl_3_) δ ppm 7.19–7.37 (m, 9 H). ^13^C{^1^H} NMR (101 MHz, CD_3_CN) δ ppm 103.6 (t, *J* = 28 Hz), 148.1 (d, *J* = 239 Hz), 163.3 (d, *J* = 255 Hz), 165.9 (m). ^19^F NMR (376 MHz, CDCl_3_) δ ppm −166.66 (s, B-C_6_F_5_) −162.62 (s, B-C_6_F_5_) −132.75 (s, B-C_6_F_5_) −91.92 (s) −85.68 (s). Elemental analysis, found (calcd for C_42_H_6_BClF_29_Sb): H: 0.62% (0.49%) C: 41.15% (41.03%).

#### **4-a** [(Ph_3_SbCl)_2_(μ-Cl)][B(C_6_F_5_)_4_]

A solution of Ph_3_SbCl_2_ (0.254 g, 0.60 mmol) in toluene (15 mL) was added to a suspension of [Et_3_Si][B(C_6_F_5_)_4_] in toluene (10 mL), freshly prepared from [Ph_3_C][B(C_6_F_5_)_4_] (0.231 g, 0.25 mmol), to yield a reddish oily suspension, which was stirred at room temperature for 90 min. Then, the solution was decanted and stood at room temperature, yielding white crystals, which were washed with *n*-hexane (3 × 5 mL) and dried in vacuo to yield a white crystalline solid (0.022 g, 0.015 mmol). ^1^H NMR (400 MHz, CDCl_3_) δ ppm 7.61–7.72 (m, 9H), 7.97–8.03 (m, 6H). ^13^C{^1^H} NMR (101 MHz, CDCl_3_) δ ppm 130.9 (s,) 133.8 (s) 134.23 (s). ^19^F NMR (376 MHz, CDCl_3_) δ ppm −166.56 (br t, *J* = 17.34 Hz), −162.85 (br t, *J* = 20.23 Hz), −132.38 (br d, *J* = 8.68 Hz). Crystals of [(Ph_3_SbCl)_2_(μ-Cl)][B(C_6_F_5_)_4_], identified by XRD, were obtained by layering a DCM solution with *n*-hexane. The red oil that dried in vacuo was then washed with *n*-hexane (3 × 5 mL) and dried in vacuo to give a light red powder, which was spectroscopically identical to **4-a**. Combined yield: 0.222 g, 0.156 mmol, 62%. A reliable elemental analysis could not be obtained probably due to impurities including **3-a** formed as a minor product in the synthesis, which would be difficult to distinguish spectroscopically.

### Gutmann–Beckett Method

The stibonium salt (0.05 mmol) and Et_3_PO (0.0012 g, 0.001 mmol) were mixed in CD_2_Cl_2_ and loaded into a J-Young NMR tube. ^1^H, ^19^F, and ^31^P{^1^H} spectra were obtained.

### Decomposition by Silane

Et_3_SiH (0.030 g, 0.26 mmol) was added to a solution of [Ph_3_SbCl][B(C_6_F_5_)_4_] (**3-a**) (0.002 g, 0.002 mmol) in CDCl_3_, giving the instant formation of a yellow solution. ^1^H and ^19^F NMR spectra were obtained and showed only peaks corresponding to **2-a** and silane. The equivalent reaction was performed with **3-e** and **4-a**.

### Stoichiometric Dehalogenation of Trityl Halide

An NMR tube was charged with a sample of [(2,4,6-F_3_C_6_H_2_)_3_SbCl][B(C_6_F_5_)_4_] (**3-e**) (0.002 g, 0.002 mmol) and Ph_3_CCl/Ph_3_CF (0.0015 g, 0.05 mmol) in CDCl_3_ (0.5 mL). A red-colored solution formed instantly on mixing. ^1^H and ^19^F NMR indicated the formation of [Ph_3_C][B(C_6_F_5_)_4_] in both cases. The equivalent reaction was also performed with [Ph_3_SbCl][B(C_6_F_5_)_4_] (**3-a**) and [(Ph_3_SbCl)_2_(μ-Cl)][B(C_6_F_5_)_4_] (**4-a**).

### Friedel–Crafts Alkylation of Benzene

An NMR tube was loaded with ^*t*^BuBr (14 mg, 0.10 mmol), benzene (16.0 mg, 0.20 mmol), and the catalyst (0.005 mmol) in CDCl_3_ (0.7 mL). The mixture was allowed to stand for 30 min, and then a ^1^H NMR spectrum was obtained. Conversion was determined by relative integration of the ^*t*^BuBr (δ_H_ = 1.81 ppm) and ^*t*^BuPh (δ_H_ = 1.34 ppm) peaks.

### Dimerization of 1,1-Diphenylethylene

A J-Young NMR tube was loaded with DPE (0.018 g, 0.1 mmol) and [(2,4,6-F_3_C_6_H_2_)_3_SbCl][B(C_6_F_5_)_4_] (**3-e**) (0.008 g, 0.005 mmol, 5% loading) in CD_2_Cl_2_ (0.7 mL). The CD_2_Cl_2_ solution turned yellow instantly on mixing. The tubes were stood for 2 h, and then ^1^H and ^19^F NMR spectra were obtained. Conversion was determined by relative integration of DPE (δ_H_ = 5.46 ppm) to a dimerized product (δ_H_ = 3.14 ppm). The equivalent reaction was also performed with [(3,5-F_2_C_6_H_3_)_3_SbCl][B(C_6_F_5_)_4_] (**3-d**) (0.007 g, 0.005 mmol, 5% loading) and **4-a.**

## Computational Methods

All calculations were performed using Gaussian 09 Revision E0.01.^39^ All geometries were optimized in the presence of a self-consistent reaction field, specifically the solvation model based on density (SMD, DCM) without imposing symmetry constraints at the M062X/def2-SVP level of theory in conjunction with the associated effective core potential on Sb. London dispersion effects were included via Grimme’s D3 atom pairwise correction.^40−42^ Counteranions were omitted from all calculations. Subsequent analytical vibrational frequency calculations on optimized geometries were utilized to confirm the nature of stationary points (zero and exactly one imaginary mode for minima and transition states, respectively). Moreover, within the ideal gas/rigid rotor/harmonic approximation, these calculations also provided thermal and entropic corrections to the Gibbs free energy at 1 atm and 298.15 K. Electronic energies were obtained from single-point calculations at the M062X-D3/def2-QZVPP (in conjunction with the associated effective core potential on Sb) level of theory including a polarizable continuum model (SMD) to account for solvent effects (parameters corresponding to those of DCM).^43^ The Kohn–Sham orbitals were visualized using Chemcraft.^44^ Single-point energies for FIAs were calculated in the gas phase using M062X-D3/def2-QZVPP^40,45^ and subsequently corrected for the solvent effect via the self-consistent reaction field (SMD with parameters corresponding to DCM). FIAs were then calculated using an isodesmic reaction, which was referenced against the defluorination reaction COF_3_^–^ → COF_2_ + F^–^. The enthalpy change for this anchor reaction was considered to be 208.8 kJ mol^–1^ from the experiment.^27^

## X-ray Crystallography

All crystallographic measurements were performed at 150 K using an Oxford Diffraction single-crystal diffractometer with a Sapphire 3 CCD plate (graphite-monochromated Mo Kα radiation, λ = 0.71073 Å or Cu Kα radiation λ = 1.54184 Å). In each case, a specimen of suitable size and quality was selected, coated with the Fomblin Y oil, and mounted onto a nylon loop. Unit cell finding, data collection, data reduction, and space group determination were performed using CrysAlis Pro. The analytical numeric absorption correction using a multifaceted crystal model was implemented.^46^ The empirical absorption correction using spherical harmonics, implemented in the SCALE3 ABSPACK scaling algorithm, was applied for absorption correction.^47^ Using Olex2,^48^ the structure was solved with the ShelXT structure solution program using intrinsic phasing and refined with the ShelXL refinement package using least-squares minimization.^49,50^ All hydrogen atoms were geometrically placed and refined using the riding model approximation.

## Supporting Information Available

Additional details of synthetic methods, crystallographic data, crystal structure figures, NMR spectra, and calculated frontier molecular orbitals (PDF)
Coordinates for computational studies (XYZ)

## Supplementary Material

om2c00426_si_001.pdf

om2c00426_si_002.xyz
