# Supplementary material for: Cationic Triarylchlorostibonium Lewis Acids
Source: Organometallics. 2023 Feb 20;42(5):339–46. doi: 10.1021/acs.organomet.2c00426 (PMC10015551; doi:10.1021/acs.organomet.2c00426)
Supplement: Supplementary file 1 — om2c00426_si_001.pdf [file om2c00426_si_001.pdf]

Electronic Supplementary Information: Cationic Triarylchlorostibonium Lewis Acids

Omar Coughlin,[a] Tobias Krämer[b] and Sophie L. Benjamin[a*]
*sophie.benjamin@ntu.ac.uk

a) Department of Chemistry, Nottingham Trent University, Clifton Lane, Nottingham, NG11 8NS, UK.
b) Department of Chemistry, Maynooth University, Maynooth, Co. Kildare, Ireland.

Contents

Synthetic Methods S1
Crystallographic data S4
Crystal structure figures S10
NMR Spectra S22
Calculated Frontier Molecular Orbitals S46
Computational investigation of alternative reaction pathways in the reduction of Ph_3SbCl^+ S47
References S49

Synthetic Methods

General

Caution: All antimony containing compounds should be treated as toxic. All manipulations were performed under an atmosphere of dry N_2 using standard Schlenk or glovebox (Mbraun Unilab 2000) techniques unless otherwise stated. All glassware was dried in an oven at 150ºC and cooled under vacuum before use. Tetrahydrofuran (THF), dichloromethane, toluene, and n-hexane were dried using an Mbraun MB SPS5. All deuterated solvents were dried and stored over 4 Å molecular sieves. $SbCl_3$ was sublimed in vacuo at 40-65°C before use. Triethylsilane was distilled over CaH_2, degassed by freeze/pump/thaw and stored over 4 Å moleuclar sieves. All other reagents were used as received unless otherwise stated. $[Et_3Si(C_7H_8)][B(C_6F_5)_4]$ was synthesised according to literature methods[1–3] and recrystalised in toluene at -10 ºC or by the addition of Et_3SiH to a solution of $[Ph_3C][B(C_6F_5)_4]$ in toluene.[4] Modified literature methods were used for the synthesis of the known compounds **1-Ar** [5–7] and **2-Ar**[8] as detailed below.

1-c $(4\text{-}FC_6H_4)_3Sb$

A solution of 4-fluorophenyl bromide (1.7523 g, 1.1 mL, 10 mmol) in diethyl ether (30 mL) was added to freshly activated magnesium turnings (5.00 g, 205 mmol) at such a rate as to maintain a reflux. The reaction mixture was stirred for 3 hours then filtered. Freshly sublimed $SbCl_3$ (0.65 g, 2.84 mmol) was added dropwise over 15 minutes to the filtered solution to form a white precipitate. The solution was stirred at room temperature for 1 hour and at reflux for a further hour. The reaction mixture was exposed to the atmosphere and H_2O was added dropwise until effervescent ceased. The diethyl ether fraction was removed and the aqueous layer was extracted with ether (30 x 3 mL). Combined extractions were dried over $MgSO_4$ and diethyl ether was removed i*n vacuo*. The resulting oily solid was recrystallized in hexanes.

Yield: 0.5567 g, 0.80 mmol 28%. ^{1}H-NMR (400MHz, $CDCl_3$,) δ ppm: 7.43-7.31 (m, 6H, o-H), 7.10 – 6.98 (m, 6H, m-H). ^{13}C{^{1}H} NMR ($CDCl_3$, 100 MHz) δ ppm: 137.90 (d, J = 7.67Hz,), 116.45 (d, J = 20.09Hz) (ortho and ipso carbon not obversed.). ^{19}F-NMR ($CDCl_3$, 376 MHz) δ ppm: -111.90 (s). Elemental Analysis, Found (Calcd for $C_{18}H_{12}F_3Sb$): C: 53.21% (53.11%) H: 3.04% (2.97%)

1-b $(3\text{-}FC_6H_4)_3Sb$

Synthesised in an equivalent manner to $(4\text{-}FC_6H_4)_3Sb$ (*vide supra*). Yield: 0.5373 g, 1.32mmol, 46%. Clear oil. Repeated crystallisation attempts failed. ^{1}H NMR (400 MHz, $CDCl_3$) δ ppm: 6.99 - 7.13 (m, 2 H, o-H) 7.20 (d, J = 7.32 Hz, 1 H, p-H) 7.35 (td, J = 7.78, 5.49 Hz, 1 H, m-H) ^{13}C{^{1}H} NMR (100 MHz, $CDCl_3$) δ ppm: 116.2 (d, J = 21 Hz) 122.44 (d, J = 19 Hz) 131.14 (dd, J = 118.8, 5 Hz) (ipso carbon not observed). ^{19}F NMR (376 MHz, $CDCl_3$) δ ppm: -111.63 (s, 1 F) Elemental Analysis, Found (Calcd for $C_{18}H_{12}F_3Sb$): C: 52.89% (53.11%) H: 2.97 % (2.97%)

1-d $(3,5\text{-}F_2C_6H_3)_3Sb$

Synthesised in an equivalent manner to $(4\text{-}FC_6H_4)_3Sb$ (*vide supra*). Yield: 0.7330g, 1.59 mmol, 56%. ^{1}H-NMR ($CDCl_3$, 400 MHz) δ ppm: 6.89-6.95 (m, 6H, o-H), 6.88 – 6.81 (tt, J = 8.92 Hz, 2.29 Hz, 3H, p-H). ^{13}C{^{1}H} NMR (100 MHz, $CDCl_3$) δ ppm 105.3 (t, J = 25 Hz), 118.3 (dd J = 9 ,6 Hz), 140.4 (s), 163.4 (dd, J = 256, 11 Hz) ^{19}F-NMR ($CDCl_3$, 376 MHz) δ ppm: -107.92 (s). Elemental Analysis, Found (Calcd for $C_{18}H_9F_6Sb$): C: 46.69% (46.89%) H: 1.67% (1.69%) Crystals suitable for SCXRD were removed from the bulk sample.

1-e $(2,4,6\text{-}F_3C_6H_2)_3Sb$

Synthesised in an equivalent manner to $(4\text{-}FC_6H_4)_3Sb$ (*vide supra*). Red oil. Repeated crystallisation attempts failed. 1H NMR (400 MHz, $CDCl_3$) δ ppm: -0.10 - 0.29 (impurity) 5.31 (impurity) 6.66 (br dd, J = 8.80, 6.29 Hz, 6 H, m-H). 7.15 (ddd, J = 7.89, 2.29, 1.26 Hz, impurity) ^{19}F NMR (376 MHz, $CDCl_3$) δ ppm: -105.60 (s, 6 F, o-F), -90.57 (s, 3 F, p-F). Due to the presence of impurities yield, elemental analysis and ^{13}C{^{1}H} NMR are not reported.

1-f $(C_6F_5)_3Sb$

Mg turnings (0.4735 g, 19.7 mmol) in diethyl ether (15 mL) were activated by the addition of a crystal of I_2, which was indicated by a slight effervesce and diminishing of the brown colour. Bromopentafluorobenzene (4.8153 g, 19.4 mmol) was added over 20 minutes at 0°C. The solution turned a brown colour over the course of the addition. This was stirred at 0°C for 30 mins, then warmed to room temperature and stirred for 1h. $SbCl_3$ (1.4258 g, 6.24 mmol) in diethyl ether (5mL) was added dropwise over 15 minutes at -15°C . The solution was slowly warmed to room temperature over 16h. The reaction mixture was exposed to the atmosphere and 0.5M HCl (5 mL) was added dropwise until effervescent ceased. The diethyl ether fraction was removed and the aqueous layer was extracted trice with diethyl ether. Decolourising charcoal was added to combined extractions and the solution was filtered through celite to result in a brown solution. The solution were dried over $MgSO_4$ and diethyl ether was removed i*n vacuo*. The resulting brown oil was dissolved in hexane and filtered through silica to result in an off-white solution, which was reduced to a colourless oil *in vacuo.* The oil solidified to a white solid at to -15°C which was isolated by vacuum filtration to give a white solid (2.920 g, 4.7mmol, 75%). ^{19}F NMR (376 MHz, $CDCl_3$) δ ppm: -158.10 (br t, *J* = 17.34 Hz) -147.67 (s) -121.37 (s). NMR spectrum matched expected values.[61]

1-g (3,5-$(CF_3)_2C_6H_3)_3Sb$

2.3M nBuLi in hexanes (4.8 mL, 11 mmol) was added dropwise to a solution of 3,5-$(CF_3)_2C_6H_3Br$ (3.2527 g, 11.10 mmol) in Et_2O (20 mL) at -78°C, yielding the instant formation of an off-white precipitate which was stirred at -78°C for 3 hours. $SbCl_3$ (0.767 g, 3.33 mmol) in Et_2O (15 mL) was added dropwise at -78°C affecting the instant formation of a deep red solution which was stirred at -78°C for 1 hour then slowly warmed to room temperature overnight, turning dark yellow and then black over the course of warming and producing a white precipitate. The reaction mixture was exposed to the atmosphere and H_2O was added dropwise until effervescent ceased. The black diethyl ether fraction was removed and the aqueous layer was extracted with diethyl ether (30 x 3 mL). Combined extractions were dried over $MgSO_4$ and diethyl ether was removed i*n vacuo*. The resulting red oil was recrystallized in hexane.

Yield: 95%. Off white solid. ^{1}H-NMR ($CDCl_3$, 400 MHz) δ ppm: 7.88 (s, 6 H, o-H) 7.96 (s, 3 H, p-H). ^{13}C{^{1}H} NMR ($CDCl_3$, 100MHz) δ ppm: 121.5 (s) 124.1 (m) 132.6 (q, *J* = 34 Hz) 135.6 (br s) 139.2 (s) ^{19}F-NMR ($CDCl_3$, 376 MHz) δ ppm: -63.65 - -62.53 (m). Elemental Analysis, Found (Calcd for $C_{24}H_9F_{18}Sb$): C: 37.71% (37.88%) H: 1.09% (1.19%) Crystals suitable for XRD were grown by layering a CH_2Cl_2 solution with hexane and cooling to -18°C.

2-c (4-$FC_6H_4)_3SbCl_2$

To a stirring solution of the **1-b** (0.2997 g, 0.75 mmol) in CH_2Cl_2 (12 mL) at -78°C, a 1M solution of SO_2Cl_2 (0.135 g, 1 mmol, 1mL) in CH_2Cl_2 was added dropwise over the course of 15 minutes. The clear solution was stirred for a further hour, when it was allowed to slowly warm to room temperature and stirred overnight. solvent was removed i*n vacuo* on a rotary evaporator. The resulting white solid was recrystallized in hexane/CH_2Cl_2.

Yield: 0.2074 g, 0.36 mmol, 48%. ^{1}H-NMR ($CDCl_3$, 400MHz) δ ppm: 8.20 -8.15 (m, 6H, o-H), 7.23 – 7.17 (m, 6H, m-H). ^{13}C{^{1}H} NMR ($CDCl_3$, 100 MHz) δ ppm: 116.9 (d, J = 21 Hz) 136.5 (d, J = 9 Hz, 1 C) ^{19}F-NMR ($CDCl_3$, 376 MHz) δ ppm: -106.06 (s). Elemental Analysis, Found (Calcd for $C_{18}H_{12}Cl_2F_3Sb$): C: 45.37% (45.23%) H: 2.39% (2.53%) Crystals suitable for XRD were removed from the bulk sample.

2-b (3-$FC_6H_4)_3SbCl_2$

Synthesised from **1-c** in an equivalent manner to (4-$FC_6H_4)_3SbCl_2$ (*vide supra*). Yield: 0.3585 g, 0.75 mmol,100%. ^{1}H NMR (400 MHz, $CDCl_3$) δ ppm: 7.28 - 7.38 (m, 1 H, m-H) 7.58 (td, J = 8.06, 5.49 Hz, 1 H, p-H) 7.96 - 8.15 (m, 2 H, o-H). ^{19}F NMR (376 MHz, $CDCl_3$) δ ppm: -108.52 (br s). ^{13}C{^{1}H} NMR (100 MHz, $CDCl_3$) δ ppm: 120.4 (dd, J = 196, 23 Hz) 130.4 (dd, J = 112, 5 Hz, 1 C) 140.5 (s, 1 C) 161.5 (s,) 164.0 (s). Elemental Analysis, Found (Calcd for $C_{18}H_{12}Cl_2F_3Sb$): C: 45.43% (45.23%) H: 2.42 % (2.53%)

2-d (3,5-$F_2C_6H_3)_3SbCl_2$

Synthesised from **1-d** in an equivalent manner to (4-$FC_6H_4)_3SbCl_2$ (*vide supra*). Yield: 0.2007 g, 0.42 mmol,56%. ^{1}H-NMR ($CDCl_3$, 400MHz): δ ppm: .84 (m, 6H, o-H/m-H), 7.08 (tt, 3H, J = 2.29 Hz, 8.46 Hz, p-H). ^{13}C{^{1}H} NMR ($CDCl_3$, 100 MHz) δ ppm: 108.0 (t, J_{C-F} = 25 Hz, p-C), 117.6 (dd, J_{C-F} = 80 Hz, 8 Hz, m-C) 140.8 (t, J_{C-F} = 7.2 Hz, ipso-C) 162.9 (dd, J_{C-F} = 512 Hz, 11 Hz, m-C) ^{19}F-NMR ($CDCl_3$, 376 MHz) δ ppm: -107.92 (s) .Elemental analysis, Found (Calcd for $C_{18}H_9Cl_2F_6Sb$): C: 40.75% (40.64%) H: 1.52% (1.71%) Single crystals suitable for XRD were grown by slow evaporation of a CH_2Cl_2 solution in air.

2-e $(2,4,6\text{-}F_3C_6H_2)_3SbCl_2$

Synthesised from crude **1-e** in an equivalent manner to $(4\text{-}FC_6H_4)_3SbCl_2$ (*vide supra*). Yield 6% (based on $SbCl_3$ used in synthesis of **1-e)**. ^{1}H NMR (400 MHz, $CDCl_3$) δ ppm 6.93 (br d, *J* = 7.20 Hz, 6 H, m-H). ^{13}C{^{1}H} NMR (100 MHz, $CDCl_3$) δ ppm 101.9 (br t, J = 25.4 Hz) 116.0 (br dd, J = 34, 10 Hz) 162.3 (br dd, J = 251, 12 Hz, 164.7 - 168.4 (m) ^{19}F NMR (376 MHz, $CDCl_3$) δ ppm -98.93 (s) -93.71 (s). Single crystals suitable for XRD were obtained from recrystallization of the bulk sample.

2-f $(C_6F_5)_3SbCl_2$

Synthesised from **1-f** in an equivalent manner to $(4\text{-}FC_6H_4)_3SbCl_2$ (*vide supra*). Yield 0.2220 g, 0.32 mmol,43 %. ^{13}C{^{1}H} NMR (100 MHz, $CDCl_3$-d) δ ppm 116.7 (br m) 137.8 (dt, J = 260 Hz, 15 Hz) 145.3 (dt, J = 261 Hz, 15 Hz).^{19}F NMR (376 MHz, $CDCl_3$) δ ppm -155.47 (br t, J = 17.34 Hz) -144.30 - -141.94 (m) -126.63 - -124.27 (m). Elemental Analysis, Found (Calcd for $C_{18}Cl_2F_{15}Sb$): C: 31.30% (31.16%) H: 0.00 % (0.00%).

2-g $(3,5\text{-}(CF_3)_2C_6H_3)_3SbCl_2$

Synthesised from **1-g** in an equivalent manner to $(4\text{-}FC_6H_4)_3SbCl_2$ (*vide supra*) Yield: 0.4659 g ,0.56 mmol,74%. ^{1}H-NMR ($CDCl_3$, 400MHz) δ ppm: 8.17 (s, 3H, p-H), 8.79 (s, 8 H, o-H) ^{13}C{^{1}H}NMR ($CDCl_3$, 100 MHz) δ ppm: 123.9 (s) 126.9 (br d, J = 3.83 Hz) 133.3 (q, J = 34.18 Hz) 134.58 (br s) 140.4 (s) ^{19}F-NMR ($CDCl_3$, 376 MHz) δ ppm: -62.69 (br m) Elemental Analysis, Found (Calcd for $C_{24}H_9Cl_2F_{18}Sb$): C: 34.52% (34.65%) H: 1.16% (1.09%). Single crystals suitable for XRD were obtained from recrystallization of the bulk sample.

Crystallographic data

General refinement considerations:

In **1-g** and **2-g** rotational disorder of the CF_3 groups has been modelled over two positions, though in the case of a few CF_3 groups it was not possible to obtain a good model and the ADP ratios remain large. In **3-a** the disorder of one aryl ring in the cation is modelled over two positions. In **2-e** there is large residual positive electron density, this is localised near to the Sb atoms and is a commonly observed result of difficulties with absorption correction for heavy atoms. **2-e·Et₃PO** was modelled as a racemic twin. Where FCF reflections are missing, this is generally the result of shadowing from the double beamstop of the Gemini instrument.

Table S1. Crystallographic refinement parameters.

Compound	1-d	1-g	2-d
Empirical formula	C18H9F6Sb	C24H9F18Sb	C18H9Cl2F6Sb
Formula weight	461.00	761.06	531.90
Temperature/K	150.00(10)	150.01(10)	150.01(10)
Crystal system	monoclinic	triclinic	monoclinic
Space group	P21/c	P-1	C2/c
a/Å	5.2441(2)	10.0041(2)	15.7714(3)
b/Å	15.5747(4)	12.0201(3)	10.8973(3)
c/Å	19.4901(6)	12.6678(3)	10.4367(2)
α/°	90	113.030(2)	90
β/°	96.736(4)	104.357(2)	102.368(2)
γ/°	90	101.497(2)	90
Volume/Å^3	1580.87(9)	1280.91(6)	1752.08(7)
Z	4	2	4
ρ_{calc} g/cm^3	1.937	1.973	2.016
μ /mm^{-1}	1.807	1.222	1.940
F(000)	888.0	732.0	1024.0
Crystal size/mm^3	0.157 × 0.126 × 0.099	0.435 × 0.403 × 0.211	? × ? × ?
Radiation	Mo Kα (λ = 0.71073)	Mo Kα (λ = 0.71073)	MoKα (λ = 0.71073)
2Θ range for data collection/°	6.716 to 61.688	6.654 to 54.968	6.44 to 54.966
Index ranges	-6 ≤ h ≤ 7, -21 ≤ k ≤ 20, -27 ≤ l ≤ 27	-12 ≤ h ≤ 12, -15 ≤ k ≤ 15, -16 ≤ l ≤ 16	-20 ≤ h ≤ 19, -13 ≤ k ≤ 14, -13 ≤ l ≤ 13
Reflections collected	24686	43694	7649
Independent reflections	4594 [R_{int} = 0.0844, R_{sigma} = 0.0865]	5848 [R_{int} = 0.0396, R_{sigma} = 0.0245]	1979 [R_{int} = 0.0209, R_{sigma} = 0.0205]
Data/restraints/parameters	4594/0/226	5848/291/556	1979/0/124
Goodness-of-fit on F_2	1.023	1.083	1.122
Final R indexes [I>=2σ (I)]	R_1 = 0.0499, wR_2 = 0.0739	R_1 = 0.0261, wR_2 = 0.0542	R_1 = 0.0185, wR_2 = 0.0410
Final R indexes [all data]	R_1 = 0.0937, wR_2 = 0.0886	R_1 = 0.0294, wR_2 = 0.0556	R_1 = 0.0198, wR_2 = 0.0414
Largest diff. peak/hole / e Å^{-3}	1.16/-0.79	0.53/-0.37	0.32/-0.63

Compound	2-e	2-g	3-a
Empirical formula	C36H12Cl4F18Sb2	C24H9Cl2F18Sb	C42H15BClF20Sb
Formula weight	1171.76	831.96	1067.55
Temperature/K	150.01(10)	150.01(10)	150.00(10)
Crystal system	orthorhombic	monoclinic	monoclinic
Space group	Pbca	C2/c	Ia
a/Å	20.9948(9)	16.284(2)	18.2922(3)
b/Å	14.5992(8)	22.8997(7)	11.1990(2)
c/Å	24.9300(16)	28.052(4)	19.0613(4)
α/°	90	90	90
β/°	90	145.53(4)	92.763(2)
γ/°	90	90	90
Volume/Å^3	7641.2(7)	5920(3)	3900.25(13)
Z	8	8	4
ρ_{calc} g/cm^3	2.037	1.867	1.818
μ /mm^{-1}	1.811	1.241	0.905
F(000)	4480.0	3200.0	2080.0
Crystal size/mm^3	0.275 × 0.19 × 0.043	0.4 × 0.102 × 0.092	0.299 × 0.174 × 0.094
Radiation	Mo Kα (λ = 0.71073)	MoKα (λ = 0.71073)	Mo Kα (λ = 0.71073)
2Θ range for data collection/°	6.66 to 58.836	6.508 to 54.966	7.278 to 61.762
Index ranges	-27 ≤ h ≤ 19, -16 ≤ k ≤ 20, -15 ≤ l ≤ 31	-20 ≤ h ≤ 20, -29 ≤ k ≤ 29, -36 ≤ l ≤ 36	-25 ≤ h ≤ 25, -15 ≤ k ≤ 16, -26 ≤ l ≤ 27
Reflections collected	20803	25543	50560
Independent reflections	8919 [R_{int} = 0.1223, R_{sigma} = 0.2279]	6597 [R_{int} = 0.0286, R_{sigma} = 0.0294]	11251 [R_{int} = 0.0684, R_{sigma} = 0.0713]
Data/restraints/parameters	8919/0/541	6597/1162/574	11251/118/641
Goodness-of-fit on F_2	0.990	1.099	1.044
Final R indexes [I>=2σ (I)]	R_1 = 0.0835, wR_2 = 0.1030	R_1 = 0.0324, wR_2 = 0.0655	R_1 = 0.0478, wR_2 = 0.0674
Final R indexes [all data]	R_1 = 0.2050, wR_2 = 0.1593	R_1 = 0.0403, wR_2 = 0.0682	R_1 = 0.0715, wR_2 = 0.0753
Largest diff. peak/hole / e Å^{-3}	1.65/-1.15	0.54/-0.35	0.55/-0.45

Compound	3-b	3-c·0.5CH_2Cl_2	3-d
Empirical formula	C42H11BClF23Sb	C42.5H13BCl2F23Sb	C42H9BClF26Sb
Formula weight	1120.52	1163.99	1175.50
Temperature/K	150.01(10)	150.01(10)	150.01(10)
Crystal system	monoclinic	monoclinic	monoclinic
Space group	P21	P21/n	Pn
a/Å	8.92125(11)	15.4617(3)	13.6723(2)
b/Å	20.4543(3)	14.4843(4)	15.5501(3)
c/Å	10.90377(15)	18.3497(5)	19.0384(3)
α/°	90	90	90
β/°	100.5011(12)	97.825(2)	94.8414(16)
γ/°	90	90	90
Volume/Å^3	1956.37(4)	4071.19(18)	4033.23(12)
Z	2	4	4
ρ_{calc} g/cm^3	1.902	1.899	1.936
μ /mm^{-1}	0.918	0.949	0.906
F(000)	1086.0	2260.0	2272.0
Crystal size/mm^3	0.456 × 0.393 × 0.181	0.303 × 0.192 × 0.19	0.164 × 0.151 × 0.086
Radiation	Mo Kα (λ = 0.71073)	Mo Kα (λ = 0.71073)	Mo Kα (λ = 0.71073)
2Θ range for data collection/°	6.516 to 61.784	6.474 to 61.682	6.488 to 54.968
Index ranges	-12 ≤ h ≤ 12, -29 ≤ k ≤ 29, -14 ≤ l ≤ 15	-21 ≤ h ≤ 21, -20 ≤ k ≤ 20, -26 ≤ l ≤ 24	-17 ≤ h ≤ 17, -20 ≤ k ≤ 19, -24 ≤ l ≤ 24
Reflections collected	43237	62976	48693
Independent reflections	11169 [R_{int} = 0.0408, R_{sigma} = 0.0428]	11917 [R_{int} = 0.0828, R_{sigma} = 0.0819]	17928 [R_{int} = 0.0576, R_{sigma} = 0.0801]
Data/restraints/parameters	11169/1/622	11917/556/640	17928/2/1280
Goodness-of-fit on F_2	1.027	1.022	1.000
Final R indexes [I>=2σ (I)]	R_1 = 0.0311, wR_2 = 0.0548	R_1 = 0.0552, wR_2 = 0.0959	R_1 = 0.0461, wR_2 = 0.0542
Final R indexes [all data]	R_1 = 0.0384, wR_2 = 0.0578	R_1 = 0.1081, wR_2 = 0.1152	R_1 = 0.0657, wR_2 = 0.0598
Largest diff. peak/hole / e Å^{-3}	0.41/-0.33	0.57/-0.70	1.08/-0.62

Compound	3-e	4-a	2-e·Et_3PO
Empirical formula	C42H6BClF29Sb	C60H30BCl3F20Sb2	$C_{15}H_{18}ClF_{4.5}OPSb_{0.5}$
Formula weight	1229.48	1491.50	427.09
Temperature/K	150.01(10)	150.01(10)	150.01(10)
Crystal system	monoclinic	monoclinic	trigonal
Space group	P21/n	P21/n	P321
a/Å	11.9508(4)	14.9294(4)	11.3249(3)
b/Å	16.2223(7)	20.3833(6)	11.3249(3)
c/Å	21.3075(7)	18.2449(5)	7.9259(2)
α/°	90	90	90
β/°	99.747(3)	92.505(2)	90
γ/°	90	90	120
Volume/$Å^3$	4071.2(3)	5546.8(3)	880.33(5)
Z	4	4	2
ρ_{calc} g/cm^3	2.006	1.786	1.611
μ /mm^{-1}	7.566	10.034	1.101
F(000)	2368.0	2912.0	428.0
Crystal size/mm^3	0.159 × 0.089 × 0.073	0.089 × 0.067 × 0.03	0.171 × 0.07 × 0.054
Radiation	CuKα (λ = 1.54184)	CuKα (λ = 1.54184)	Mo Kα (λ = 0.71073)
2Θ range for data collection/°	13.254 to 133.202	7.344 to 143.912	6.61 to 58.706
Index ranges	-14 ≤ h ≤ 11, -19 ≤ k ≤ 17, -25 ≤ l ≤ 25	-16 ≤ h ≤ 11, -24 ≤ k ≤ 24, -22 ≤ l ≤ 21	-15 ≤ h ≤ 15, -15 ≤ k ≤ 15, -10 ≤ l ≤ 10
Reflections collected	17949	29392	13627
Independent reflections	7132 [R_{int} = 0.0723, R_{sigma} = 0.1006]	10148 [R_{int} = 0.0845, R_{sigma} = 0.1467]	1507 [R_{int} = 0.0542, R_{sigma} = 0.0363]
Data/restraints/parameters	7132/0/667	10148/0/661	1507/24/74
Goodness-of-fit on F_2	0.991	1.144	1.147
Final R indexes [I>=2σ (I)]	R_1 = 0.0573, wR_2 = 0.1183	R_1 = 0.0734, wR_2 = 0.1546	R_1 = 0.0548, wR_2 = 0.1463
Final R indexes [all data]	R_1 = 0.0907, wR_2 = 0.1375	R_1 = 0.1190, wR_2 = 0.1786	R_1 = 0.0609, wR_2 = 0.1510
Largest diff. peak/hole / e $Å^{-3}$	0.63/-1.27	1.18/-1.04	1.72/-1.23

**Compound**	**$[(4\text{-}FC_6H_4)_4Sb][B(C_6F_5)_4]$**
**Empirical formula**	C48H16BF24Sb
**Formula weight**	1181.17
**Temperature/K**	150.01(10)
**Crystal system**	triclinic
**Space group**	P-1
**a/Å**	11.2248(3)
**b/Å**	13.1814(3)
**c/Å**	15.2737(3)
**α/°**	84.529(2)
**β/°**	79.518(2)
**γ/°**	86.792(2)
**Volume/Å^3**	2210.32(9)
**Z**	2
**ρ_{calc} g/cm^3**	1.775
**μ /mm^{-1}**	0.762
**F(000)**	1152.0
**Crystal size/mm^3**	0.27 × 0.148 × 0.088
**Radiation**	Mo Kα (λ = 0.71073)
**2Θ range for data collection/°**	6.478 to 57.398
**Index ranges**	-15 ≤ h ≤ 15, -17 ≤ k ≤ 17, -20 ≤ l ≤ 20
**Reflections collected**	56571
**Independent reflections**	11387 [R_{int} = 0.0634, R_{sigma} = 0.0609]
**Data/restraints/parameters**	11387/0/667
**Goodness-of-fit on F_2**	1.032
**Final R indexes [I>=2σ (I)]**	R_1 = 0.0492, wR_2 = 0.0952
**Final R indexes [all data]**	R_1 = 0.0825, wR_2 = 0.1092
**Largest diff. peak/hole / e Å^{-3}**	0.51/-0.44

Crystal structure figures

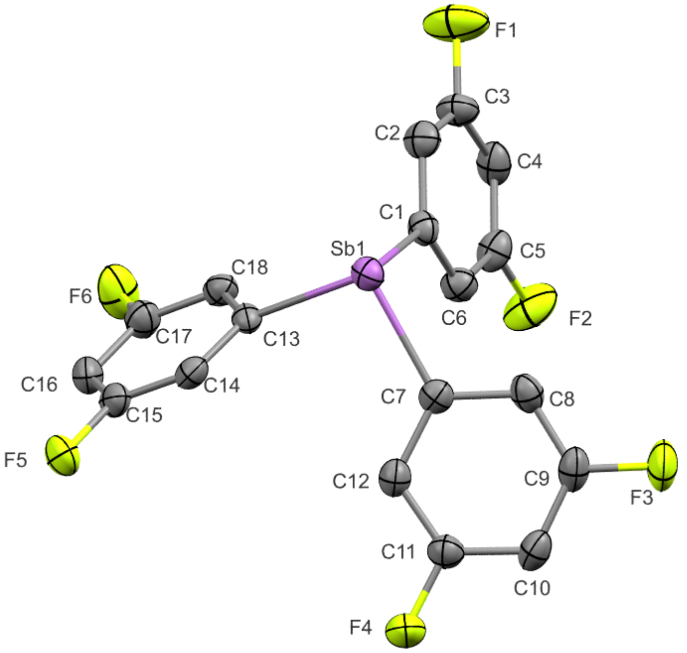

Figure S1. Solid state structure of **1-d**. Ellipsoids shown at 50% probability. Hydrogen atoms have been omitted. Selected structural parameters (bond lengths in angstroms and bond angles in degrees): Sb1-C13 2.159(4), Sb1-C7 2.156(4), Sb1-C1 2.159(4), C13-Sb1-C1 95.33(15), C7-Sb1-C13 97.76(15) C7-Sb1-C1 94.38(15).

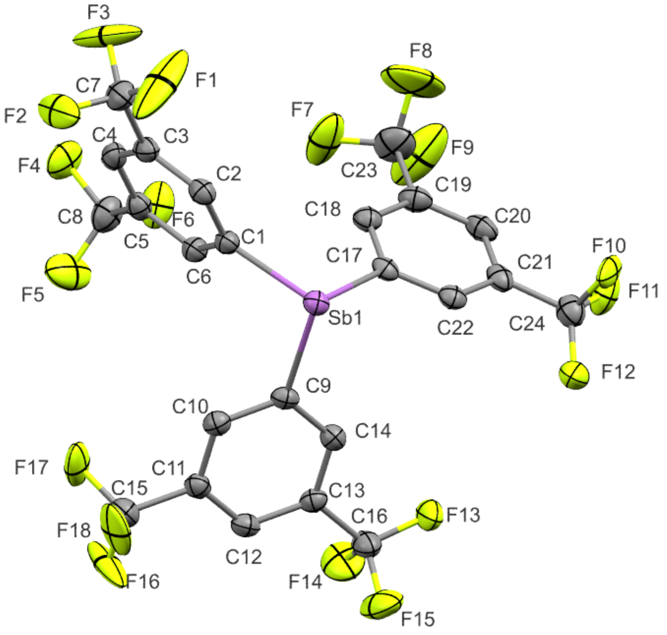

Figure S2. Solid state structure of **1-g**. Ellipsoids shown at 50% probability. Hydrogen atoms have been omitted. The trifluoromethyl groups are disordered and modelled over two positions, only one is shown. Selected structural parameters (bond lengths in angstroms and bond angles in degrees): Sb1-C17 2.155(2), Sb1-C1 2.144(2), Sb1-C9 2.160(2), C17-Sb1-C9 94.93(8), C1-Sb1-C17 97.05(8), C1-Sb1-C9 96.04(8).

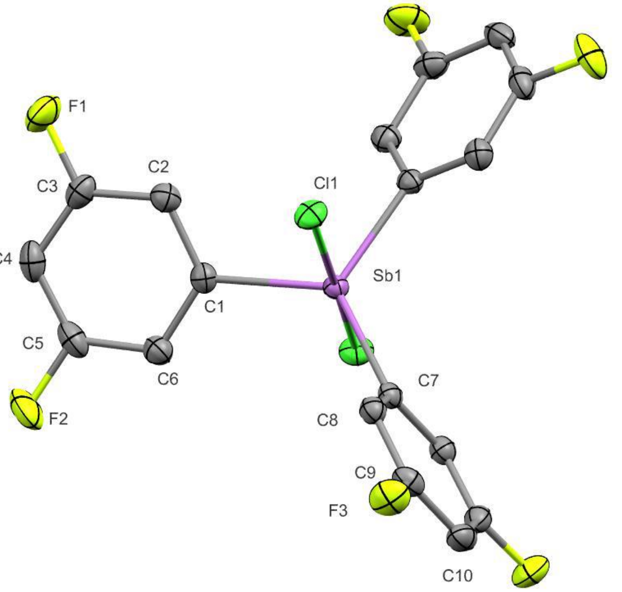

Figure S3. Solid state structure of **2-d**. Ellipsoids shown at 50% probability. Hydrogen atoms have been omitted. Selected structural parameters (bond lengths in angstroms and bond angles in degrees): Sb1-Cl1 2.4555(4), Sb1-C7 2.128(3), Sb1-C11 2.1089(18), Sb1-C1 2.1089(18), Cl1-Sb1-Cl11 176.02(2), C7-Sb1-Cl1$_1$ 91.991(12), C7-Sb1-Cl1 91.991(12),C1-Sb1-Cl1 89.01(5),C1-Sb1-Cl1$_1$ 88.86(5),C1$_1$-Sb1-Cl1$_1$ 89.01(5),C1$_1$-Sb1-Cl1 88.86(5),C1$_1$-Sb1-C7 122.25(5),C1-Sb1-C7 122.25(5),C1-Sb1-C1$_1$ 115.51(10). $_1$1-X,+Y,1/2-Z

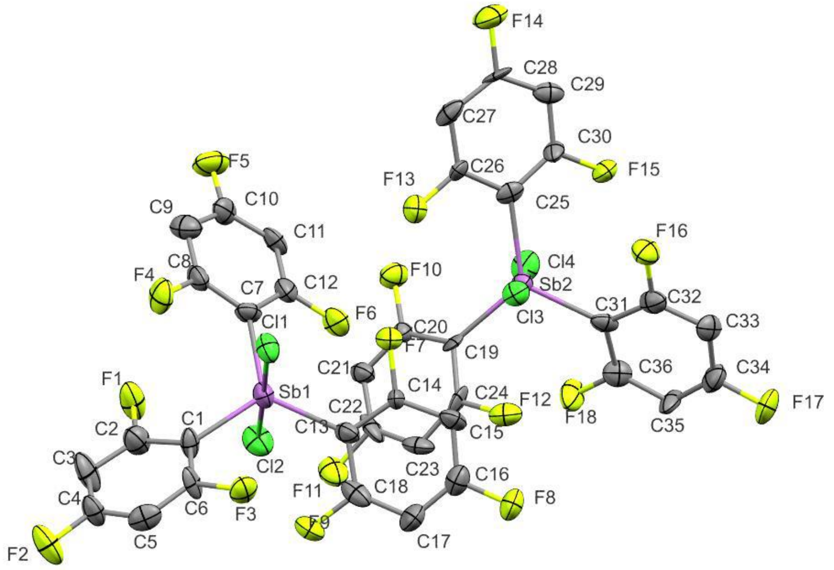

Figure S4. Solid state structure of **1-e.** Ellipsoids shown at 50% probability. Hydrogen atoms have been omitted. Selected structural parameters (bond lengths in angstroms and bond angles in degrees): Sb2-C25 2.092(10), Sb2-C19 2.086(11), Sb2-C31 2.061(12), Sb2-Cl3 2.395(3), Sb2-Cl4 2.422(3), Sb1-C13 2.094(12), Sb1-C1 2.082(11), Sb1-C7 2.091(10), Sb1-Cl2 2.408(3), Sb1-Cl1 2.433(3), C25-Sb2-Cl3 87.2(3), C25-Sb2-Cl4 88.5(3), C19-Sb2-C25 123.4(5), C19-Sb2-Cl3 93.3(3), C19-Sb2-Cl4 88.2(3), C31-Sb2-C25 123.1(5), C31-Sb2-C19 113.4(4), C31-Sb2-Cl3 92.5(3), C31-Sb2-Cl4 90.7(3), Cl3-Sb2-Cl4 175.58(10), C13-Sb1-Cl2 89.9(3), C13-Sb1-Cl1 91.1(3), C1-Sb1-C13 117.8(4), C1-Sb1-C7 117.3(5), C1-Sb1-Cl2 90.3(3), C1-Sb1-Cl1 89.4(3), C7-Sb1-C13 124.8(5), C7-Sb1-Cl2 89.8(3), C7-Sb1-Cl1 89.5(3), Cl2-Sb1-Cl1 178.99(13).

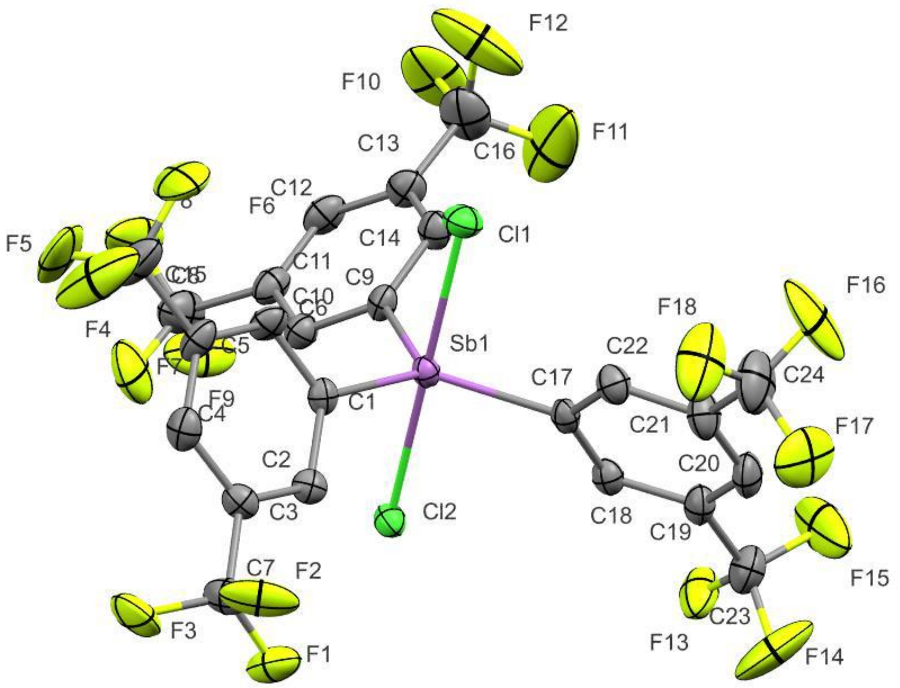

Figure S5. Solid state structure of **2-g**. Ellipsoids shown at 50% probability. Hydrogen atoms have been omitted. The trifluoromethyl groups are disordered and modelled over two positions, only one is shown. Selected structural parameters (bond lengths in angstroms and bond angles in degrees): Sb1-Cl2 2.4275(7), Sb1-Cl1 2.4330(7), Sb1-C1 2.117(3), Sb1-C17 2.120(2), Sb1-C9 2.117(3), Cl2-Sb1-Cl1 178.88(2), C1-Sb1-Cl2 90.25(8), C1-Sb1-Cl1 90.71(8), C1-Sb1-C17 120.21(10), C17-Sb1-Cl2 90.00(7), C17-Sb1-Cl1 90.02(7), C9-Sb1-Cl2 89.43(8), C9-Sb1-Cl1 89.62(8), C9-Sb1-C1 117.16(10), C9-Sb1-C17 122.63(10).

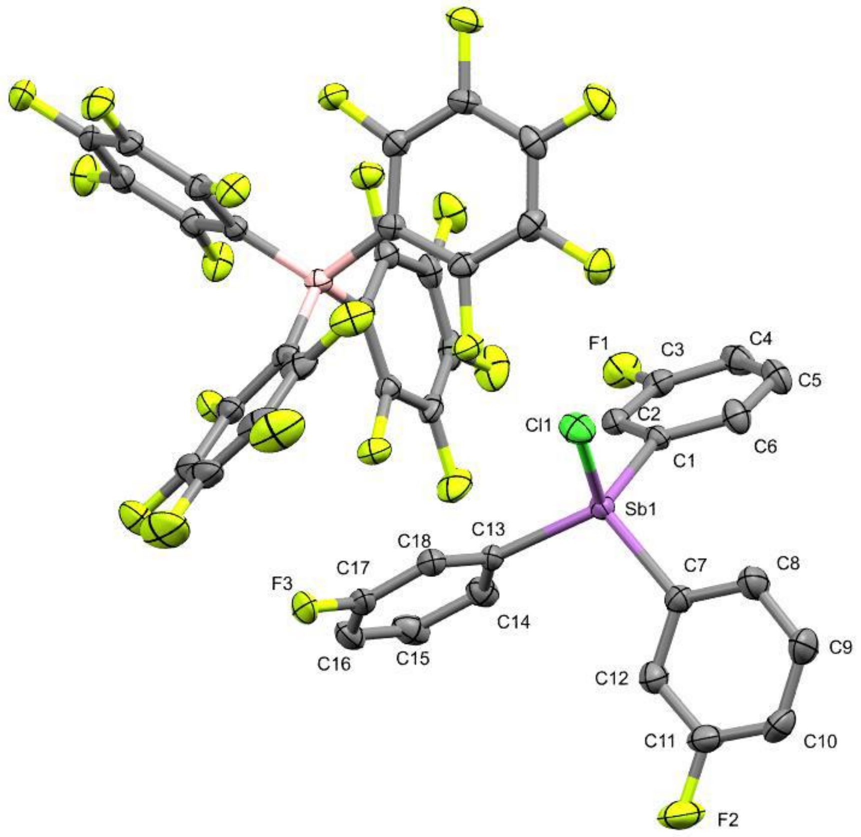

Figure S6. Solid state structure of **3-b**. Ellipsoids shown at 50% probability. Hydrogen atoms and labels for borate counteranion have been omitted. F3 is disordered over two positions, only one is shown. SHELXD (dual space) was used to provide an initial structure solution. Selected structural parameters (bond lengths in angstroms and bond angles in degrees): Sb1-Cl1 2.2762(9), Sb1-C1 2.087(3), Sb1-C13 2.084(3), Sb1-C7 2.088(4), C1-Sb1-Cl1 109.52(10), C1-Sb1-C7 110.06(14), C13-Sb1-Cl1 106.07(10), C13-Sb1-C1 115.28(13),C13-Sb1-C7 108.49(14),C7-Sb1-Cl1 107.05(10).

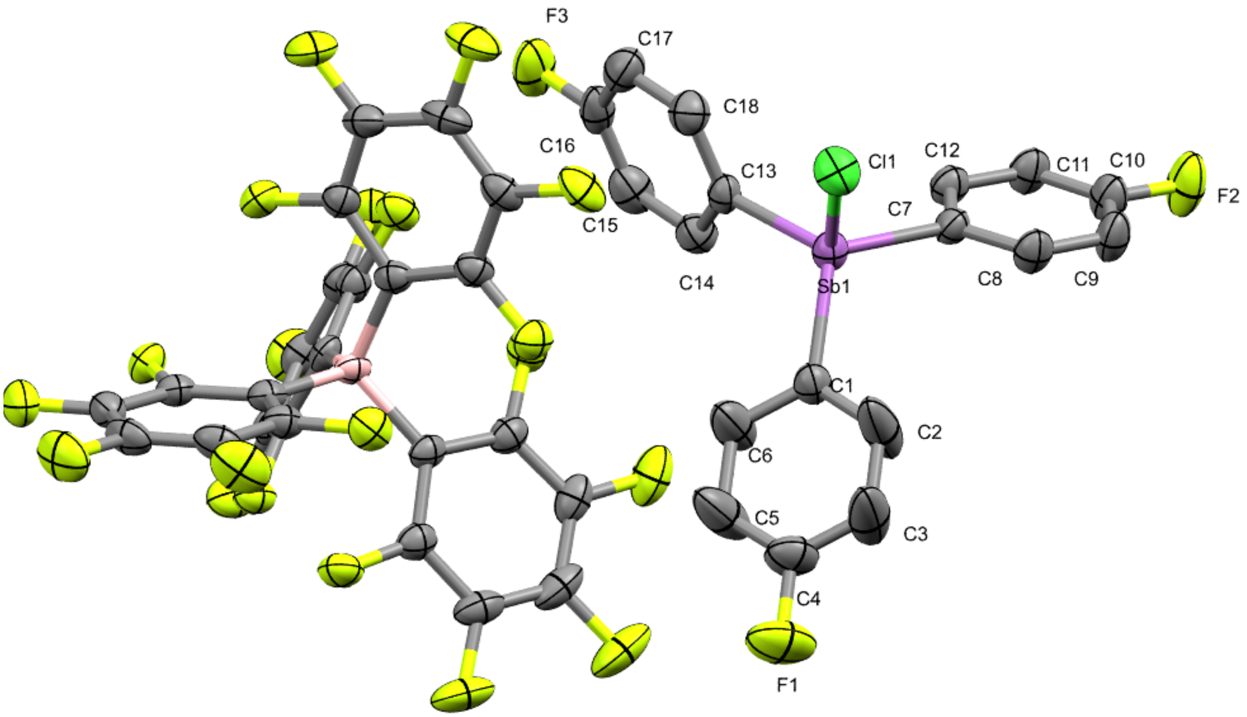

Figure S7. Solid state structure of **3-c.** Ellipsoids shown at 50% probability. Hydrogen atoms, disordered co-crystalised dichloromethane and labels for borate counteranion have been omitted. Selected structural parameters (bond lengths in angstroms and bond angles in degrees): Sb1-Cl1 2.2923(10), Sb1-C13 2.087(3), Sb1-C7 2.084(3), Sb1-C1 2.085(4), C13-Sb1-Cl1 105.58(10), C7-Sb1-Cl1 105.94(10), C7-Sb1-C13 109.66(13), C7-Sb1-C1 114.48(14),C1-Sb1-Cl1 103.77(10),C1-Sb1-C13 116.29(14).

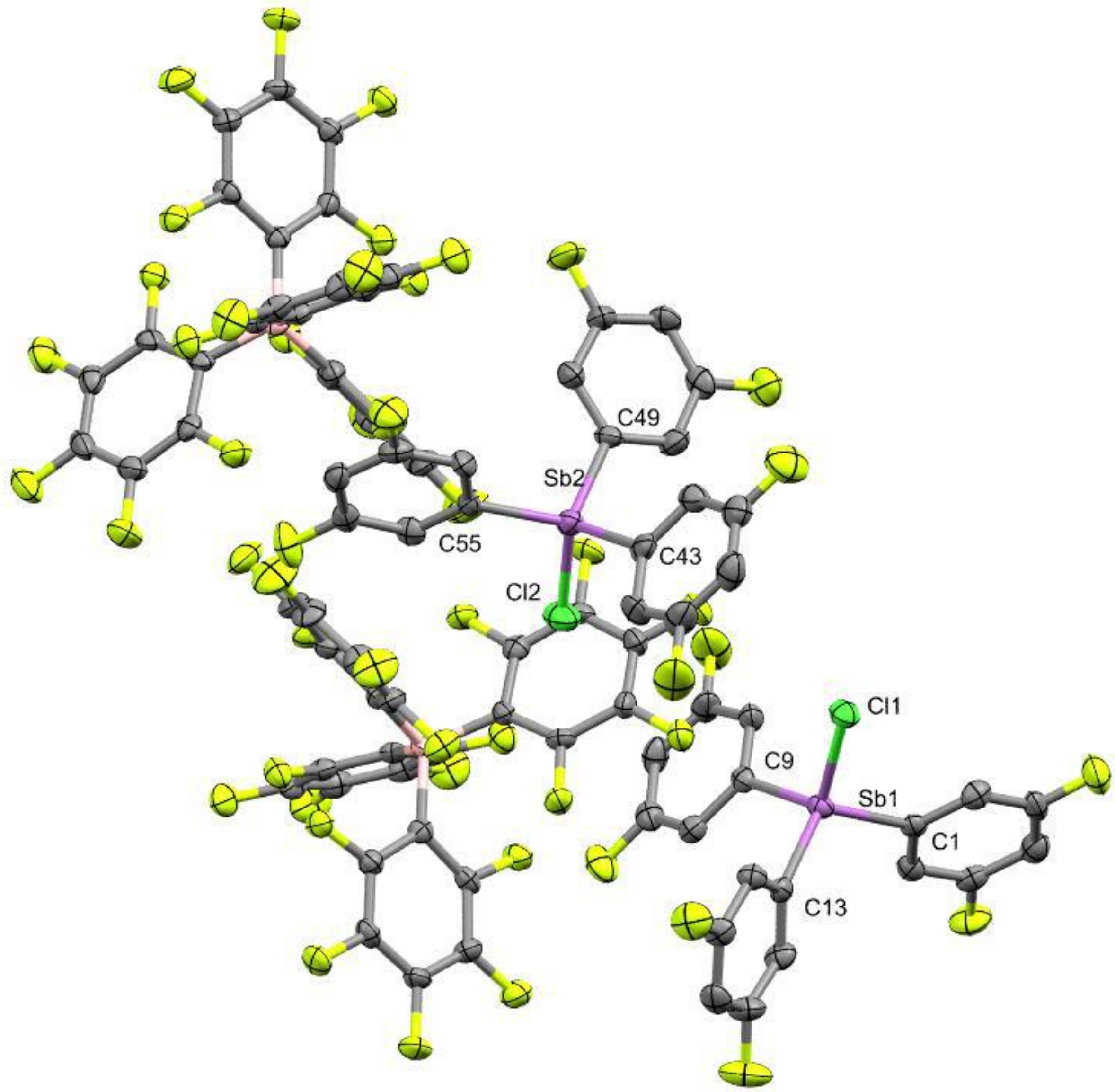

Figure S8. Solid state structure of **3-d.** Ellipsoids shown at 50% probability. Hydrogen atoms have been omitted and only selected labels are shown. The structure is racemically twinned (TWIN LAW (-1.0, 0.0, 0.0, 0.0, -1.0, 0.0, 0.0, 0.0, -1.0), BASF [0.480(12)]) Selected structural parameters (bond lengths in angstroms and bond angles in degrees): Sb1-Cl1 2.2608(16), Sb1-C13 2.096(7), Sb1-C9 2.096(6), Sb1-C1 2.088(7), Sb2-Cl2 2.2604(19), Sb2-C49 2.088(7), Sb2-C55 2.090(6), Sb2-C43 2.093(7), C13-Sb1-Cl1 109.11(19) C9-Sb1-Cl1 110.72(18), C9-Sb1-C13 107.9(3), C1-Sb1-Cl1 105.4(2), C1-Sb1-C13 116.4(2), C1-Sb1-C9 107.3(3), C49-Sb2-Cl2 108.9(2), C49-Sb2-C55 112.2(3), C49-Sb2-C43 108.1(3), C55-Sb2-Cl2 107.1(2), C55-Sb2-C43 112.9(3), C43-Sb2-Cl2 107.5(2).

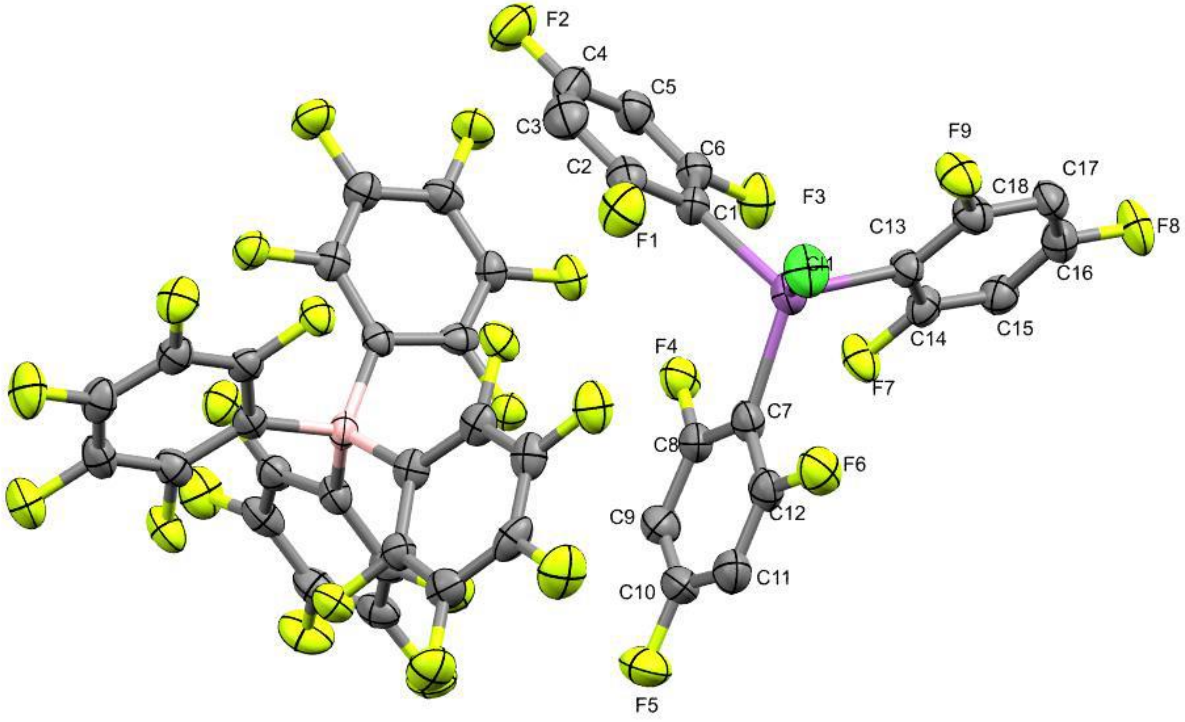

Figure S9. Solid state structure of **1-e.** Ellipsoids shown at 50% probability. Hydrogen atoms and labels for borate counteranion have been omitted. Selected structural parameters (bond lengths in angstroms and bond angles in degrees): Sb1-Cl12.2532(19), Sb1-C13 2.067(6), Sb1-C1 2.084(6), Sb1-C7 2.072(7), C13-Sb1-Cl1 108.2(2), C1-Sb1-C1 111.1(3), C13-Sb1-C7 112.7(3), C1-Sb1-Cl1 106.2(2), C7-Sb1-Cl1 107.2(2), C7-Sb1-C1 111.1(2).

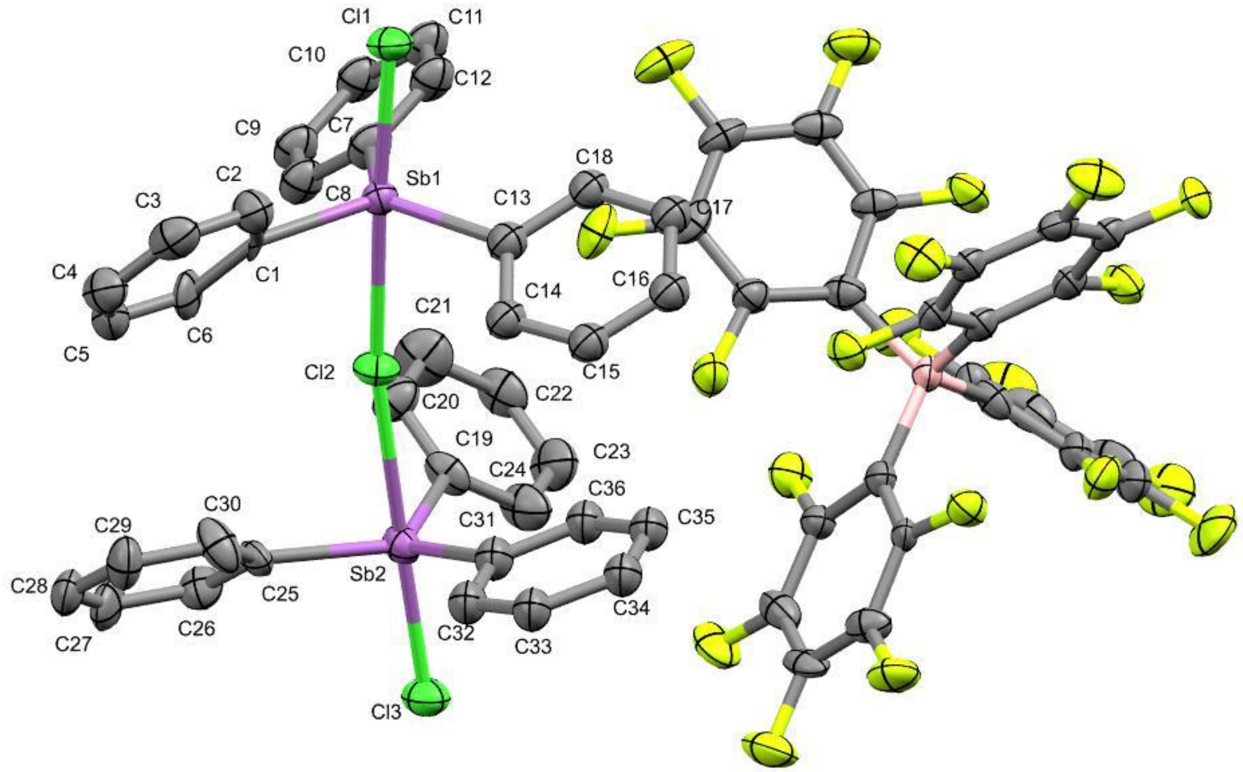

Figure S10. Solid state structure of **4-a.** Ellipsoids shown at 50% probability. Hydrogen atoms and labels for borate counteranion have been omitted. Selected structural parameters (bond lengths in angstroms and bond angles in degrees):Sb1-Cl2 2.679(3), Sb1-Cl1 2.388(3), Sb1-C7 2.123(11), Sb1-C13 2.104(12), Sb1-C1 2.120(9), Sb2-Cl2 2.782(3), Sb2-Cl3 2.372(3), Sb2-C25 2.105(10), Sb2-C19 2.109(9), Sb2-C31 2.087(12), Cl1-Sb1-Cl2 176.39(10), C7-Sb1-Cl2 84.4(4), C7-Sb1-Cl1 93.2(4), C13-Sb1-Cl2 86.1(3), C13-Sb1-Cl1 93.0(3), C13-Sb1-C7 123.4(4), C13-Sb1-C1 114.1(4), C1-Sb1-Cl2 87.2(3), C1-Sb1-Cl1 96.3(3), C1-Sb1-C7 121.0(4), Cl3-Sb2-Cl2 178.39(11), C25-Sb2-Cl2 83.7(3), C25-Sb2-Cl3 96.8(3), C25-Sb2-C19 118.3(5), C19-Sb2-Cl2 86.9(3), C19-Sb2-Cl3 94.2(3), C31-Sb2-Cl2 81.2(3), C31-Sb2-Cl3 97.2(4), C31-Sb2-C25 119.1(4), C31-Sb2-C19 119.2(4).

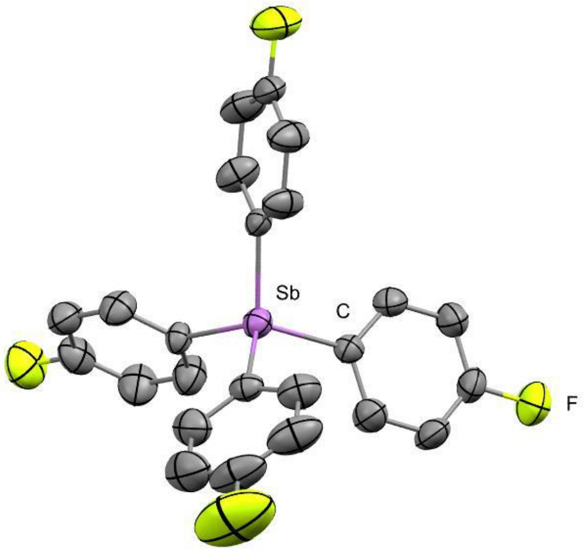

Figure S11. Solid state structure of the cation from $[(4\text{-}FC_6H_4)_4Sb][B(C_6F_5)_4]$. Ellipsoids shown at 50% probability. Hydrogen atoms and labels for borate counteranion have been omitted. Selected structural parameters (bond lengths in angstroms and bond angles in degrees): Sb1-C1 2.082(3), Sb1-C19 2.099(3), Sb1-C13 2.096(3), Sb1-C7 2.084(3), C1-Sb1-C19 108.34(13) C1-Sb1-C13 112.66(12), C1-Sb1-C7 109.90(12), C13-Sb1-C19 109.85(13), C7-Sb1-C19 110.43(12), C7-Sb1-C13 105.65(13).

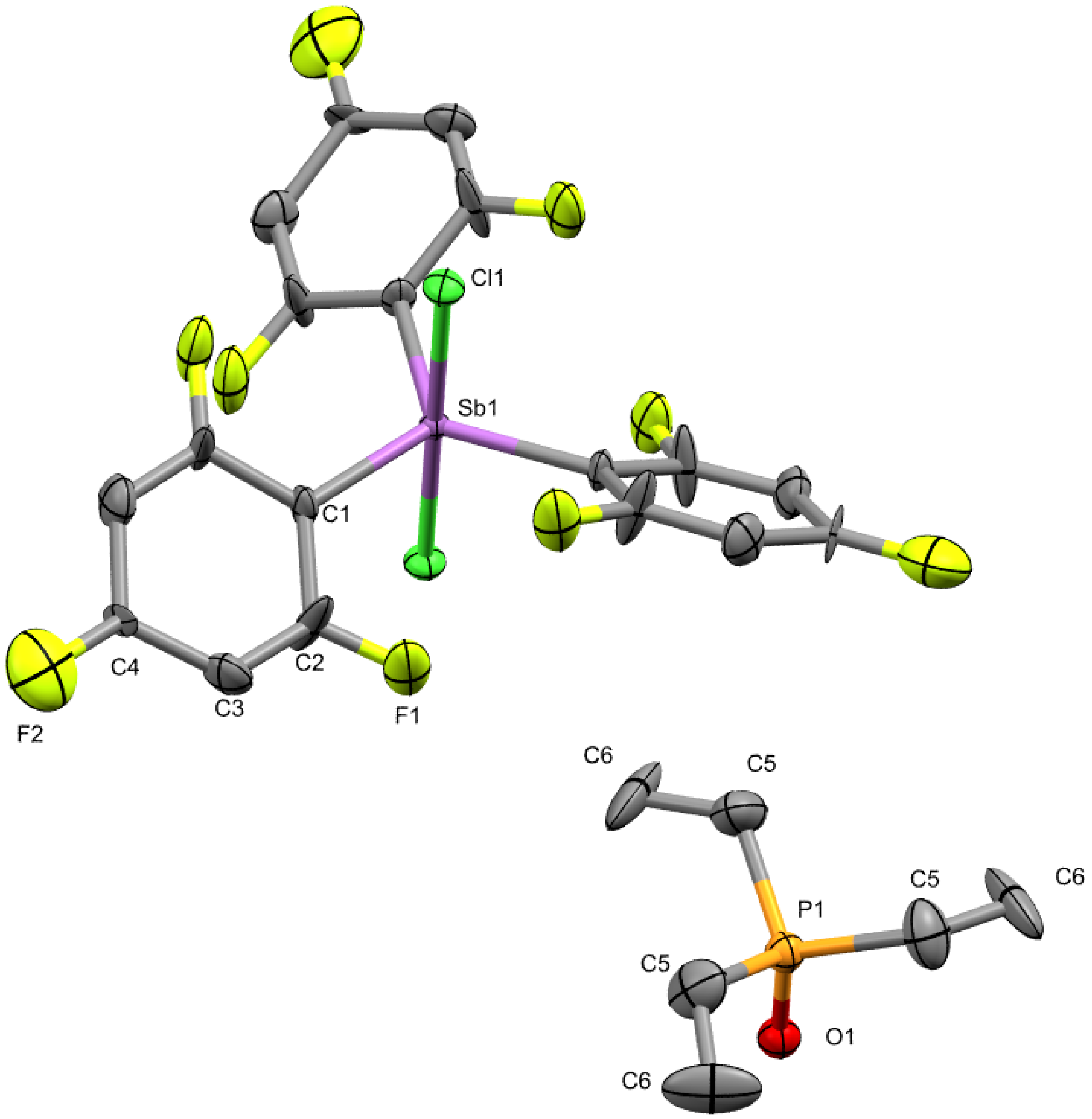

Figure S12. Solid state structure of **2-e·Et_3PO.** Ellipsoids shown at 50% probability. Hydrogen atoms and symetry eqiuvalent labels have been omitted. The structure is racemically twinned (TWIN LAW (-1.0, 0.0, 0.0, 0.0, -1.0, 0.0, 0.0, 0.0, -1.0), BASF [0.480(12)]). Selected structural parameters (bond lengths in angstroms and bond angles in degrees): Sb1-Cl1 2.404(3), Sb1-C1 2.085(12), P1-O1 1.496(9), Cl1-Sb1-Cl1$_1$ 180.0, C1-Sb1-Cl1 90.0, C1$_2$-Sb1-Cl1 90.000(1), C1-Sb1-C1$_3$ 120.000(2), C1-Sb1-C1$_2$ 120.0.

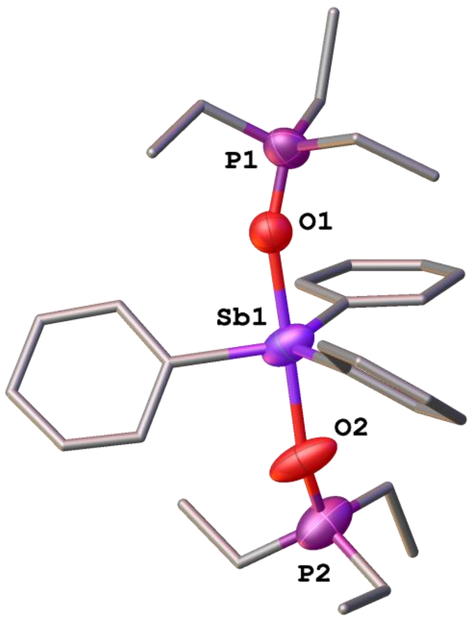

Figure S13. Solid state structure of $[Ph_3Sb(OPEt_3)_2]^{2+}$ included to show the connectivity only. Ellipsoids shown at 50% probability for heavy atoms, C atoms are shown in wireframe. Hydrogen atoms and two incompletely modelled OTf^- anions have been omitted. Selected structural parameters are not included due to the poor data quality and incomplete modelling of triflates.

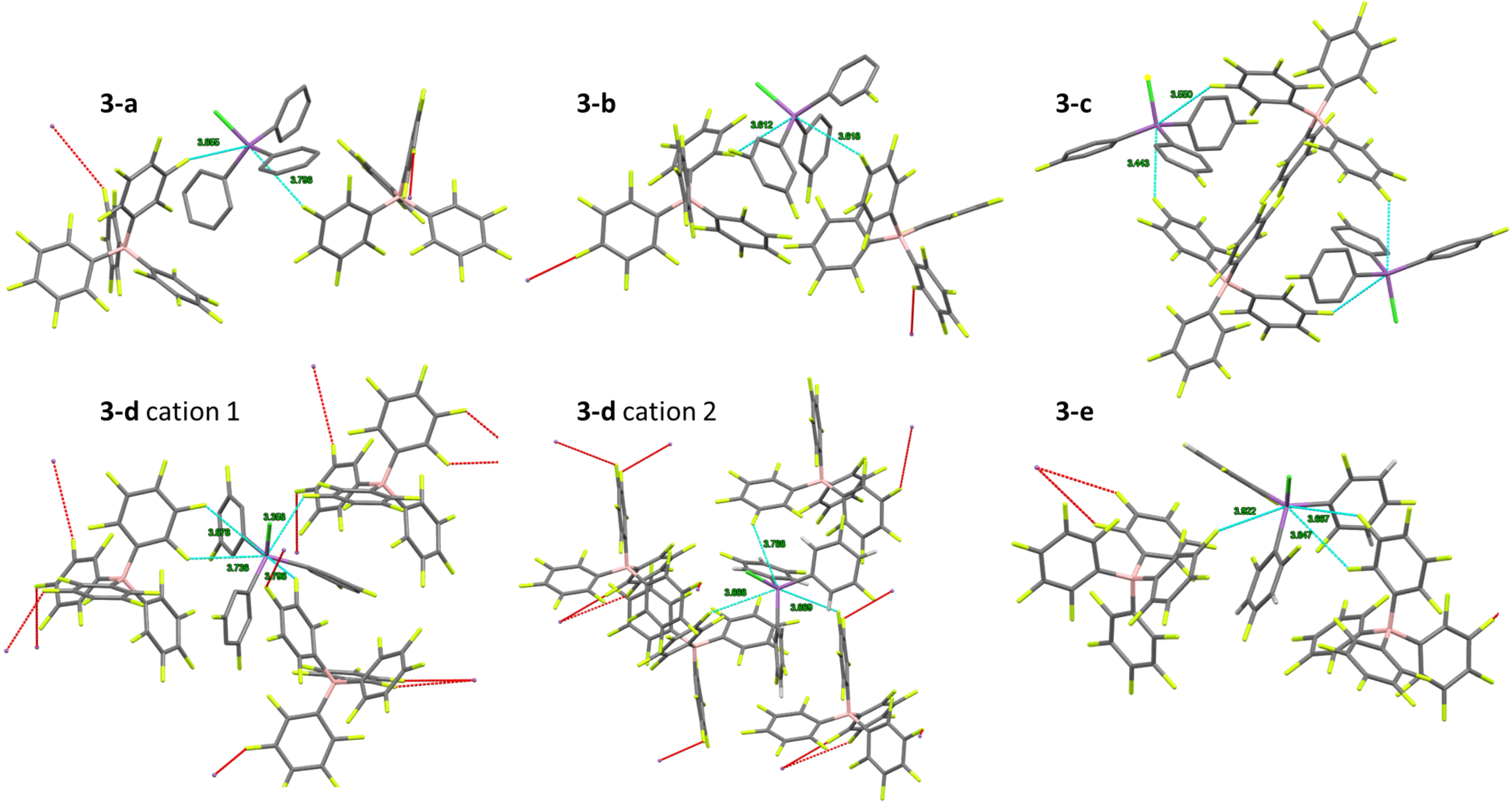

Figure S14. Depiction of cation-anion contacts in the solid-state structures of **3-Ar**.

NMR Spectra

1-b (3-$FC_6H_4)_3Sb$

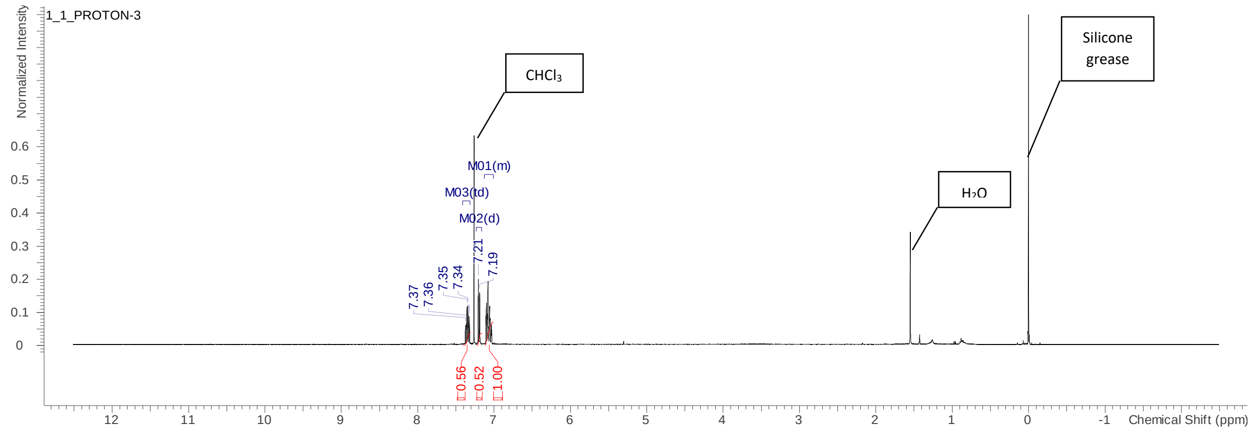

Figure S15. ^{1}H NMR spectrum of **1-b** in $CDCl_3$.

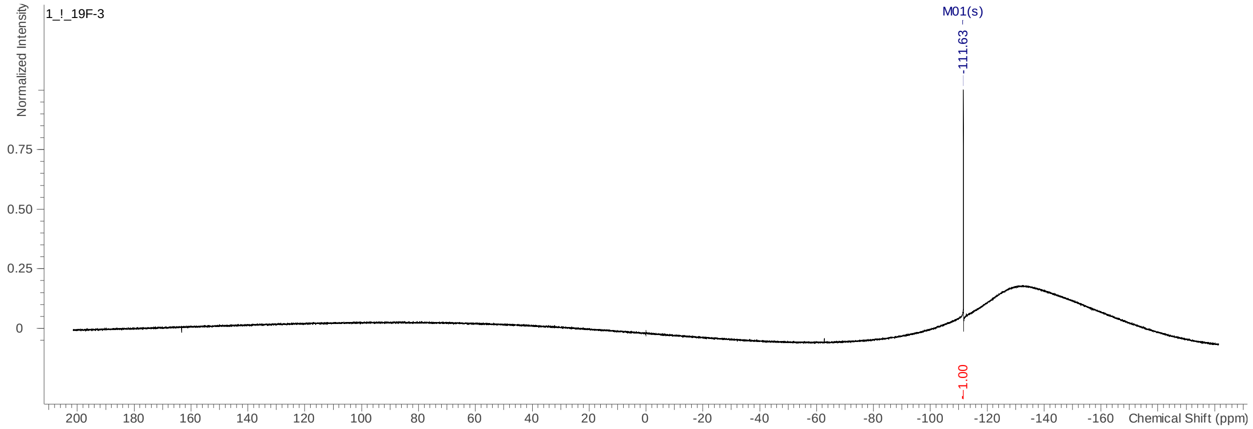

Figure S16. $^{13}C\{^{1}H\}$ NMR spectrum of **1-b** in $CDCl_3$.

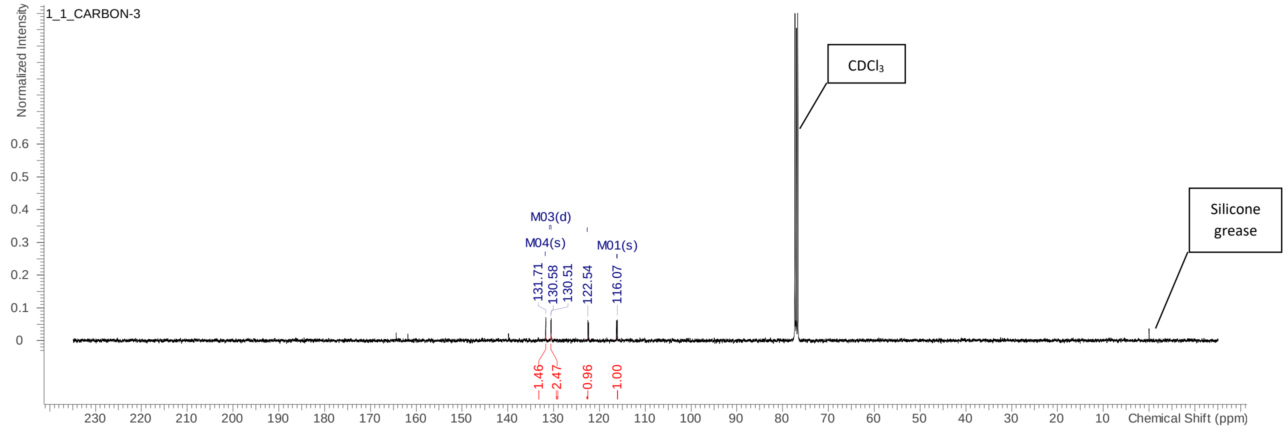

Figure S17. ^{19}F NMR spectrum of **1-b** in $CDCl_3$.

1-c $(4\text{-}FC_6H_4)_3Sb$

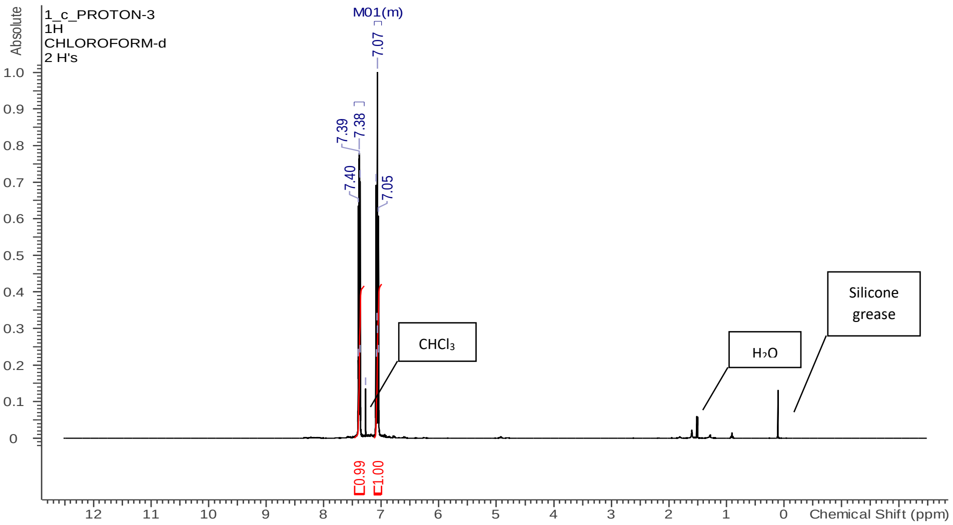

Figure S18. ^1H NMR spectrum of **1-c** in $CDCl_3$.

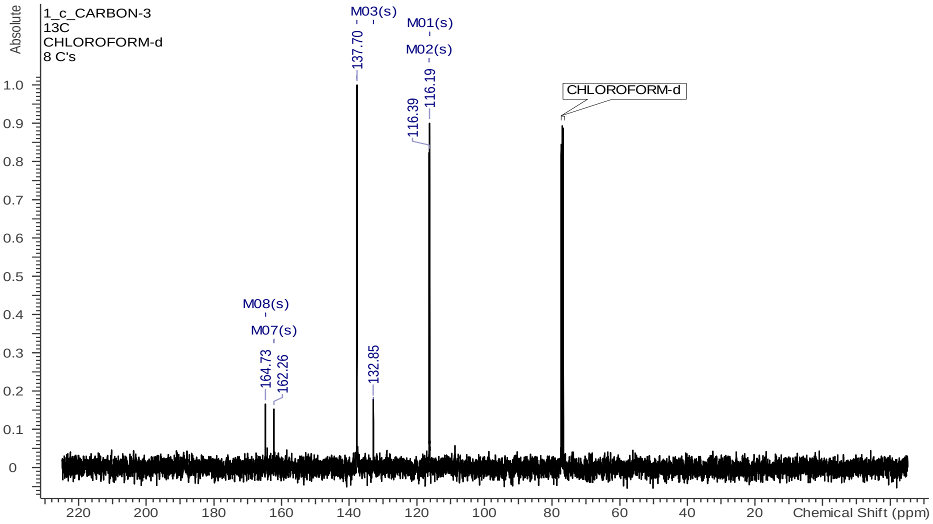

Figure S19. $^{13}C\{^1H\}$ NMR spectrum of **1-c** in $CDCl_3$.

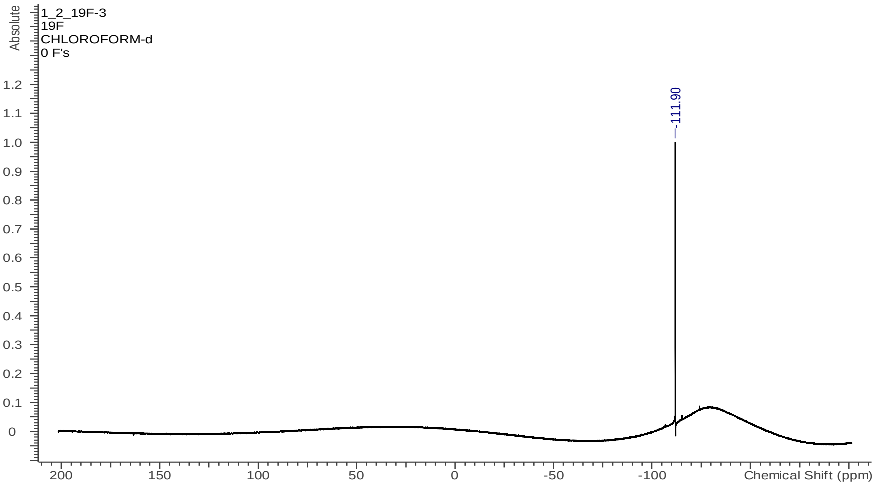

Figure S20. ^{19}F NMR spectrum of **1-c** in $CDCl_3$.

1-d $(3.5\text{-}F_2C_6H_3)_3Sb$

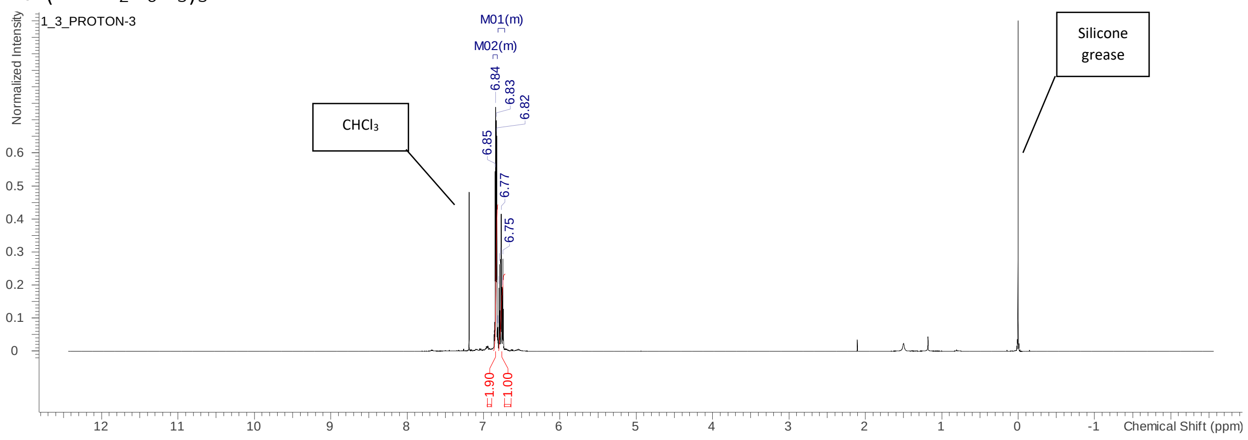

Figure S21. ^{1}H NMR spectrum of **1-d** in $CDCl_3$.

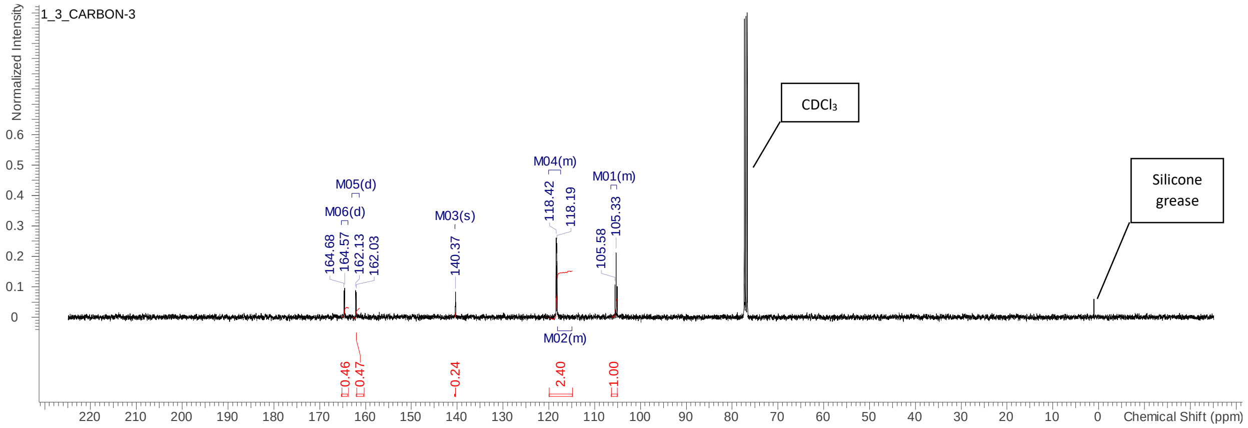

Figure S22. $^{13}C\{^{1}H\}$ NMR spectrum of **1-d** in $CDCl_3$.

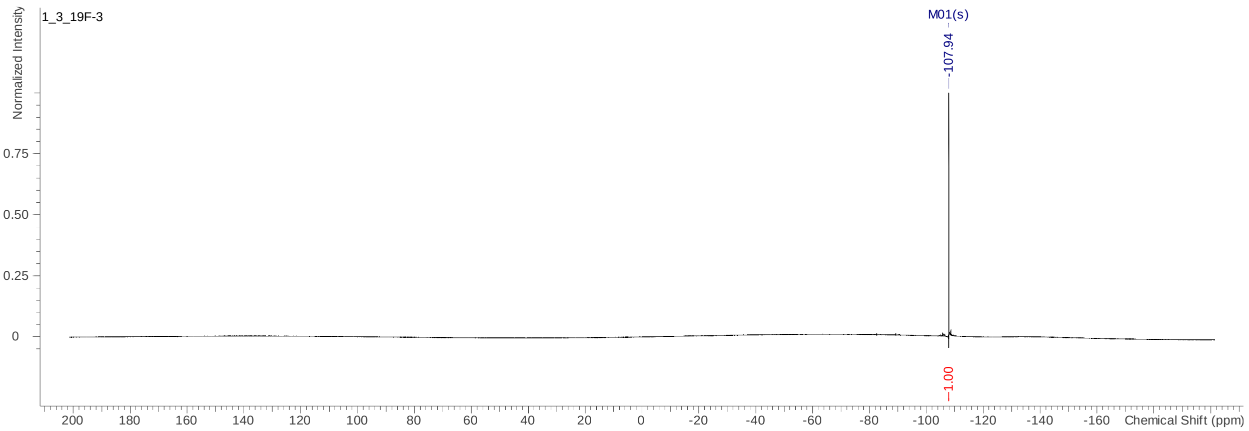

Figure S23. ^{19}F NMR spectrum of **1-d** in $CDCl_3$.

1-e (2,4,6-$F_3C_6H_2$)$_3$Sb

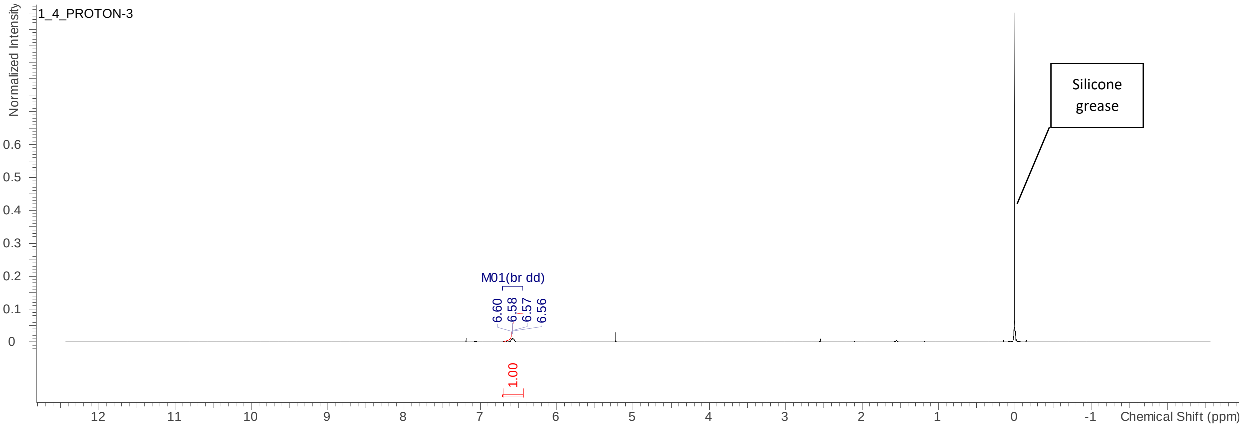

Figure S24. ^{1}H NMR spectrum of **1-e** in $CDCl_3$.

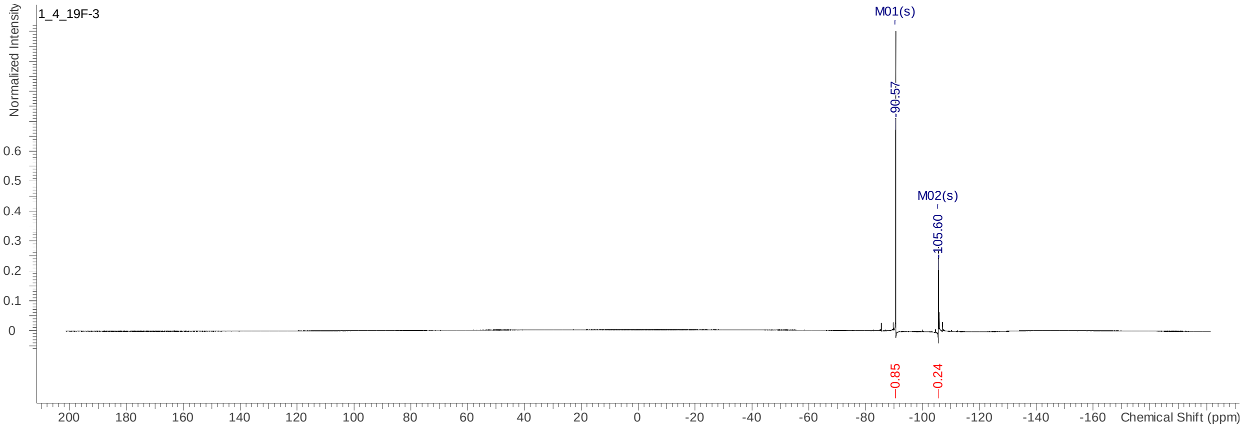

Figure S25. ^{13}C{^{1}H} NMR spectrum of **1-e** in $CDCl_3$.

1-g $(3,5-(CF_3)_2C_6H_3)_3Sb$

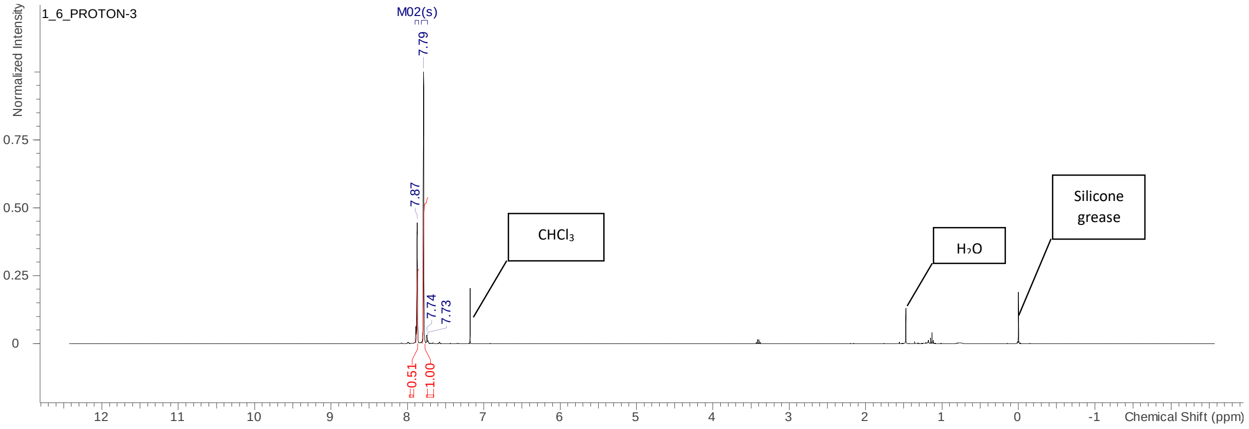

Figure S26. ^1H NMR spectrum of **1-g** in $CDCl_3$.

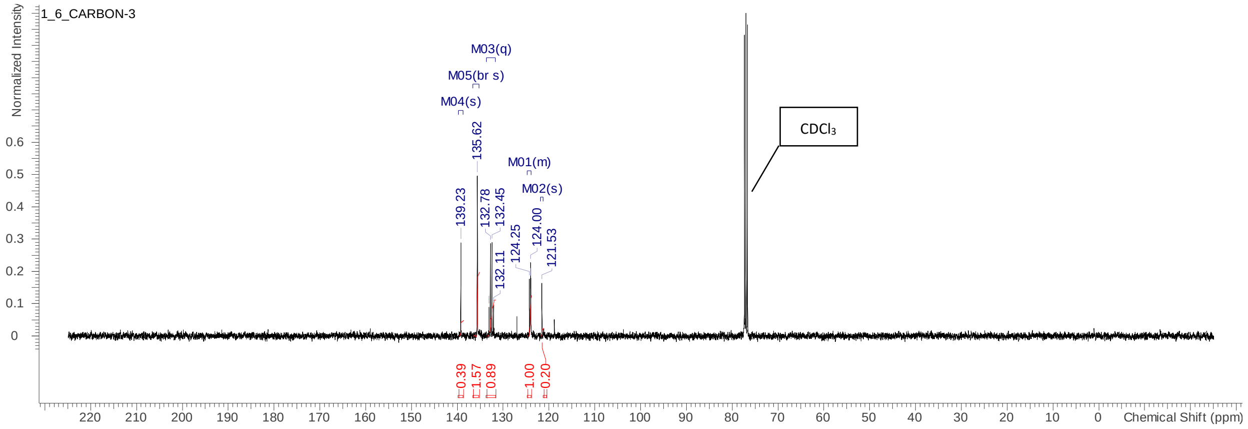

Figure S27. $^{13}C\{^1H\}$ NMR spectrum of **1-g** in $CDCl_3$.

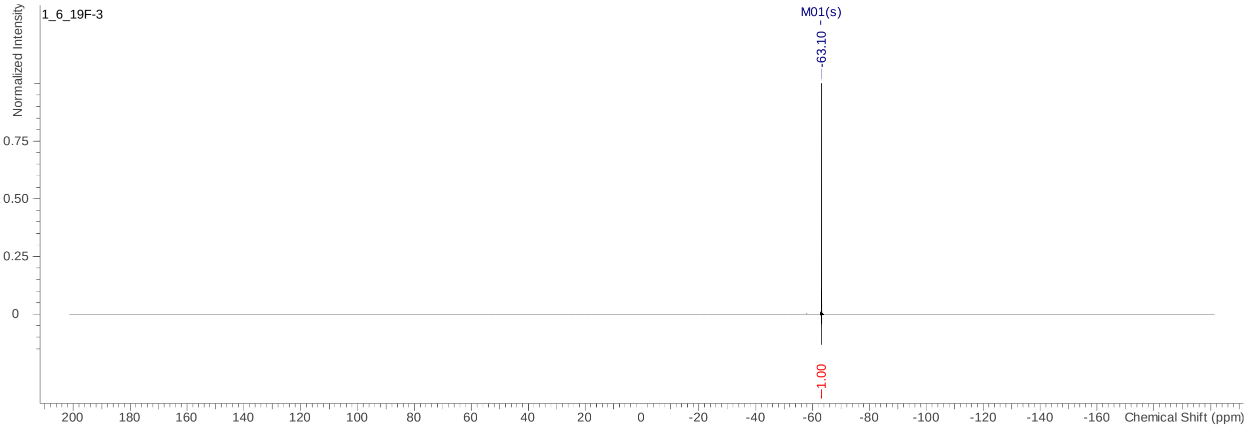

Figure S28. ^{19}F NMR spectrum of **1-g** in $CDCl_3$.

2-b $(3\text{-}FC_6H_4)_3SbCl_2$

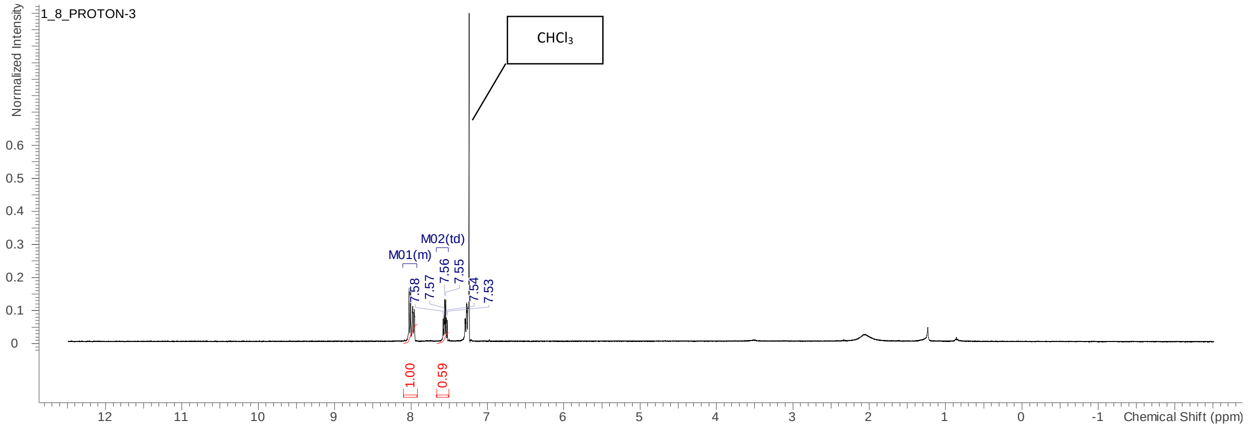

Figure S29. ^{1}H NMR spectrum of **2-b** in $CDCl_3$.

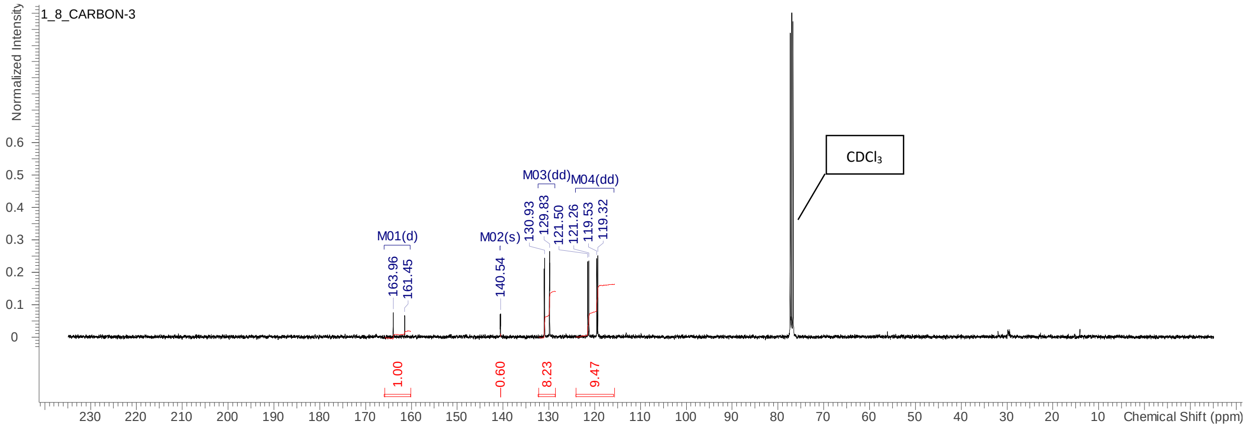

Figure S30. $^{13}C\{^{1}H\}$ NMR spectrum of **2-b** in $CDCl_3$.

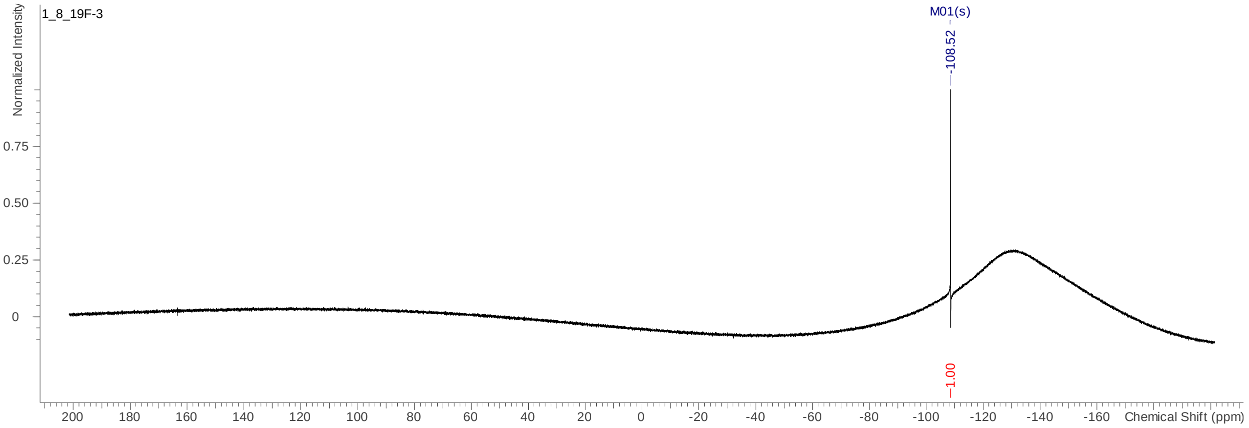

Figure S31. ^{19}F NMR spectrum of **2-b** in $CDCl_3$.

2-c $(4\text{-}FC_6H_4)_3SbCl_2$

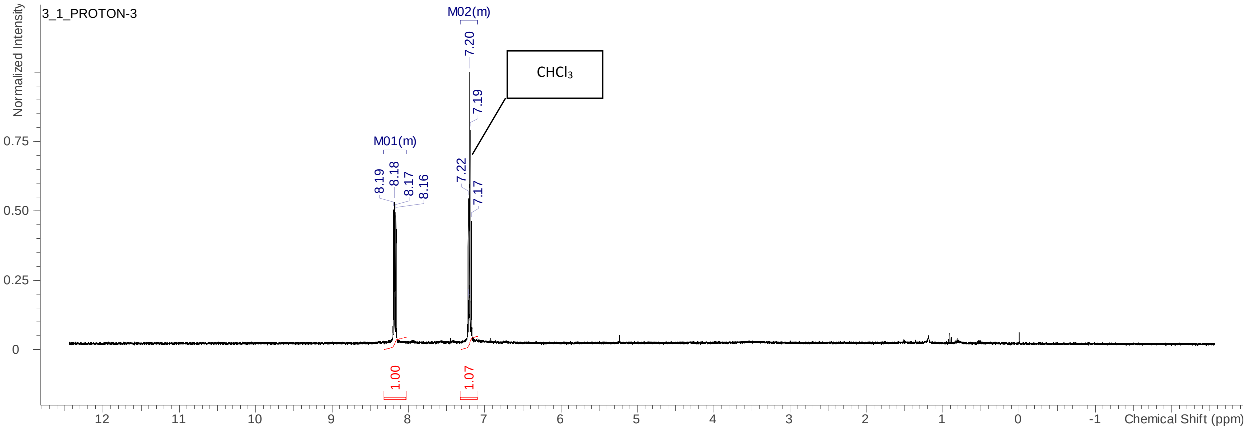

Figure S32. ^{1}H NMR spectrum of **2-c** in $CDCl_3$.

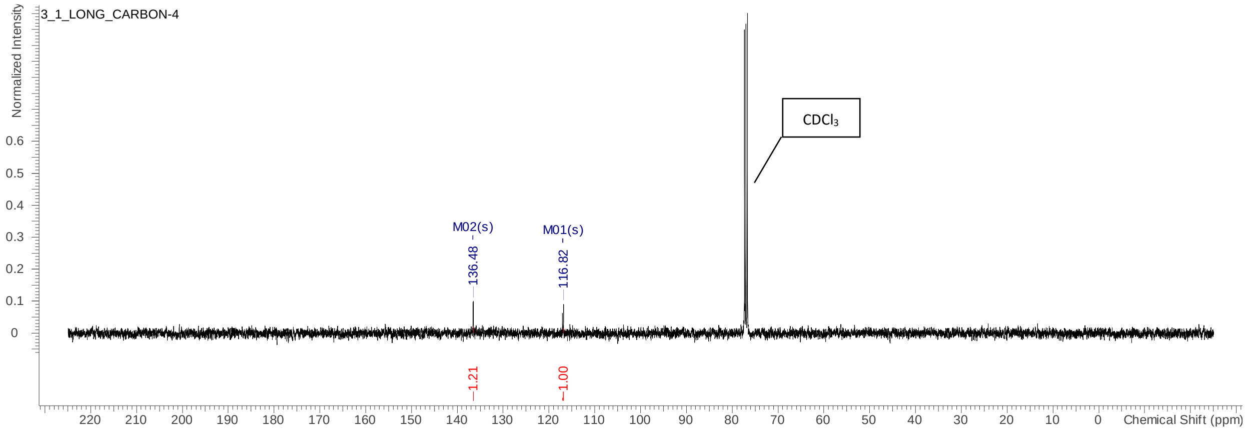

Figure S33. $^{13}C\{^{1}H\}$ NMR spectrum of **2-c** in $CDCl_3$.

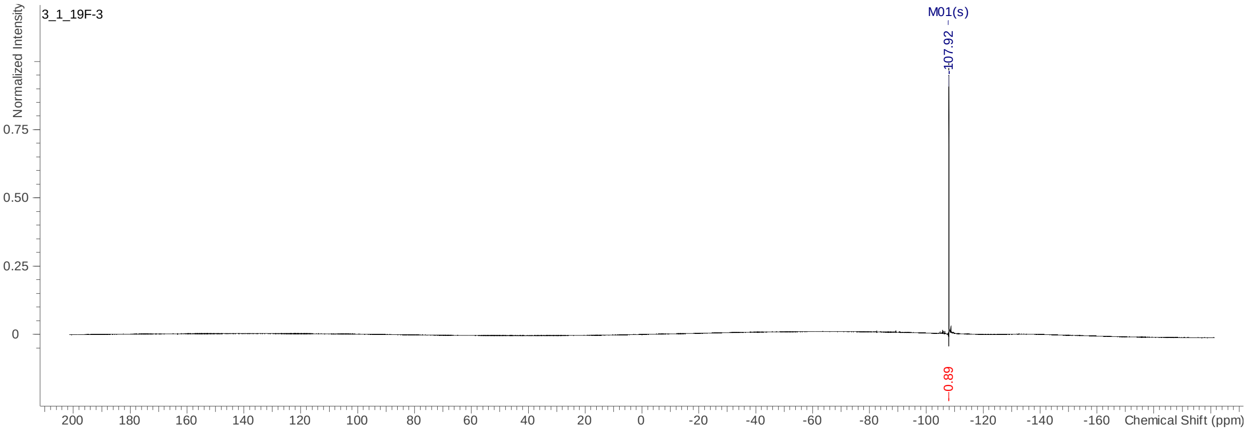

Figure S34. ^{19}F NMR spectrum of **2-c** in $CDCl_3$.

2-d $(3,5-F_2C_6H_3)_3SbCl_2$

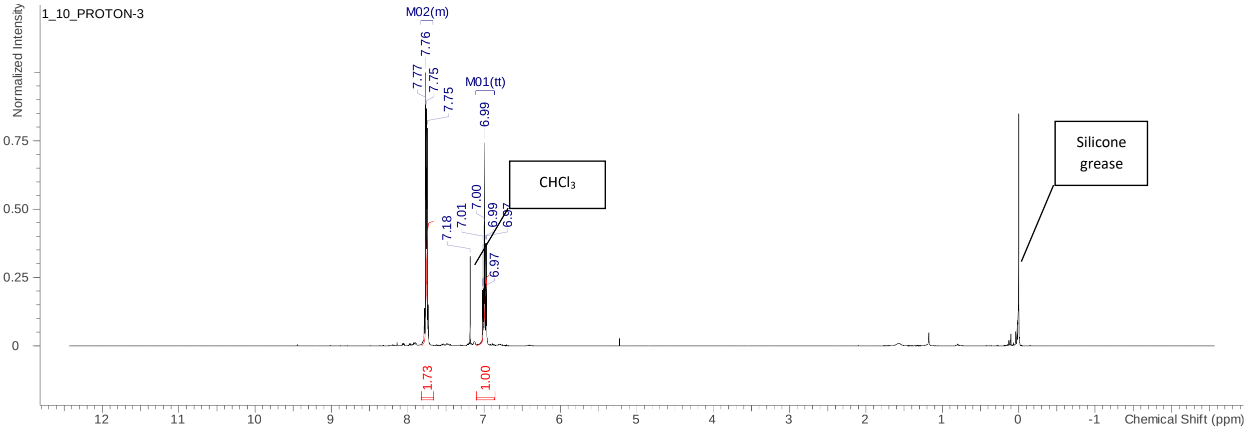

Figure S35. ^{1}H NMR spectrum of **2-d** in $CDCl_3$.

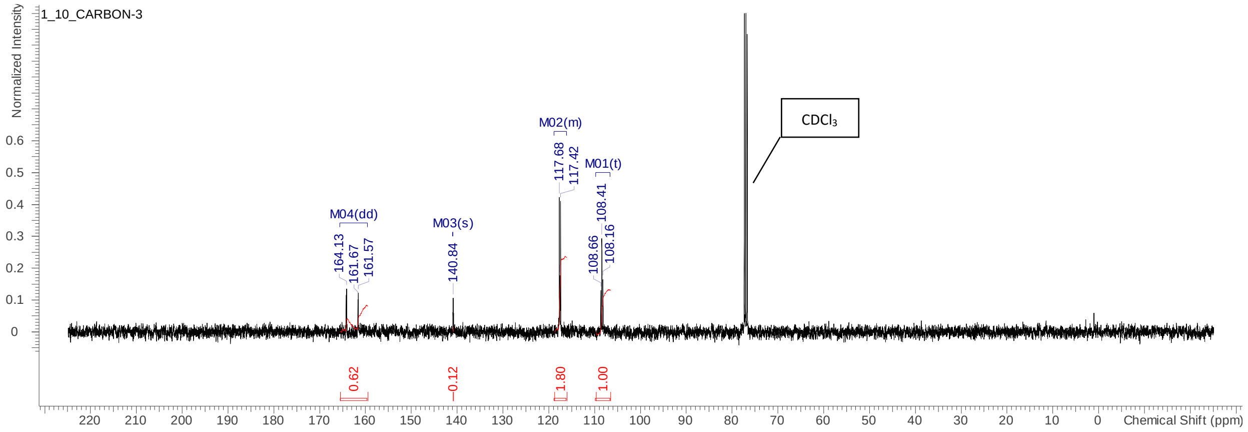

Figure S36. $^{13}C\{^{1}H\}$ NMR spectrum of **2-d** in $CDCl_3$.

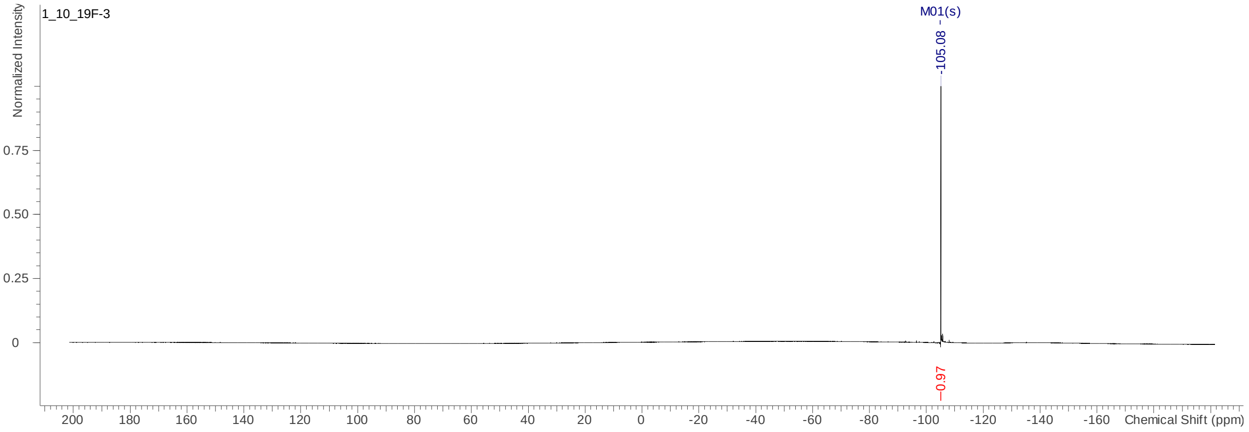

Figure S37. ^{19}F NMR spectrum of **2-d** in $CDCl_3$.

2-e $(2,4,6\text{-}F_3C_6H_2)_3SbCl_2$

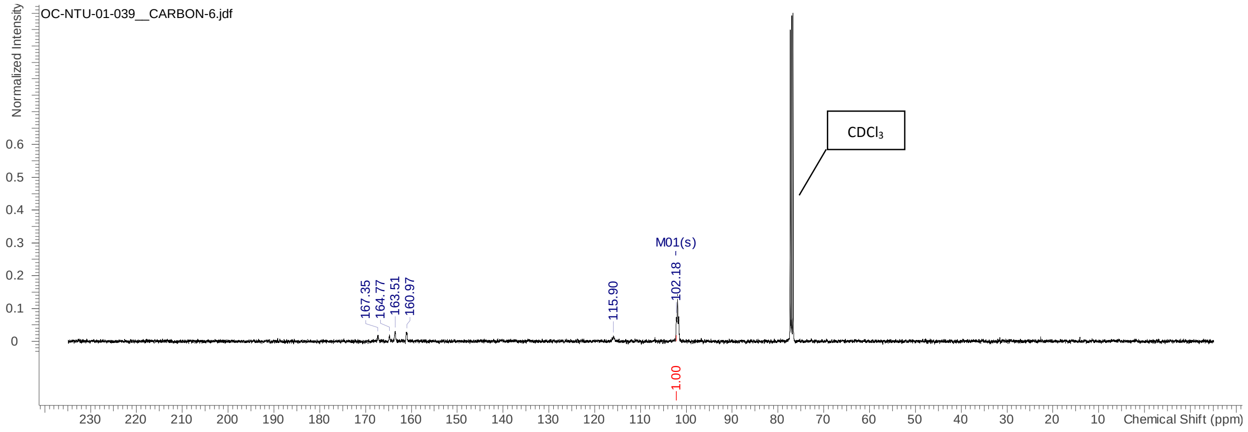

Figure S38. ^1H NMR spectrum of **2-e** in $CDCl_3$.

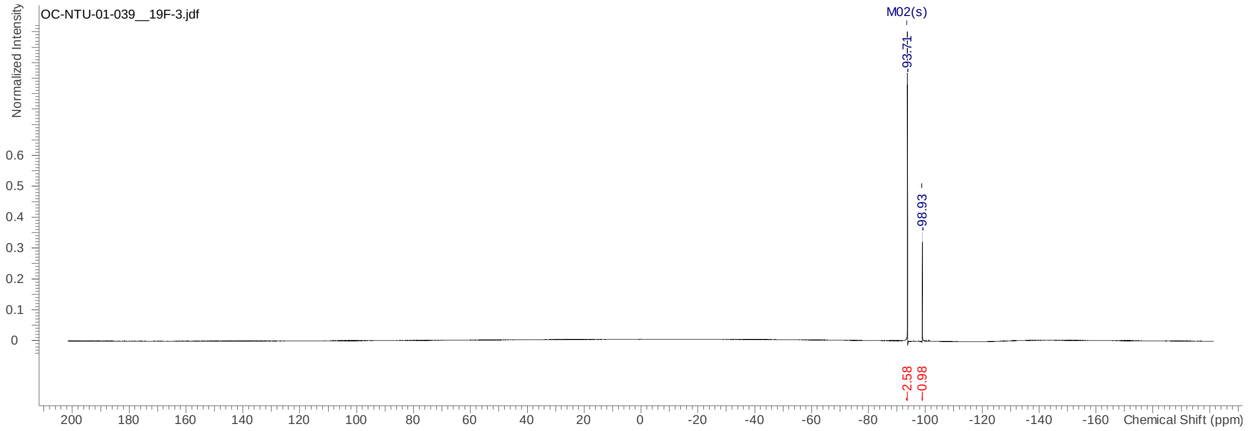

Figure S39. ^{19}F NMR spectrum of **2-e** in $CDCl_3$.

2-f $(C_6F_5)_3SbCl_2$

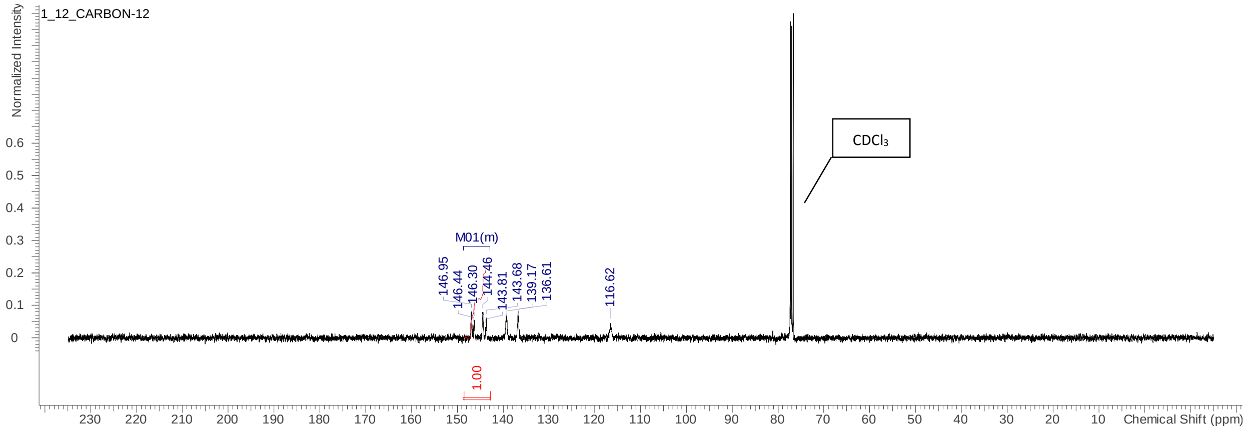

Figure S40. $^{13}C\{^{1}H\}$ NMR spectrum of **2-f** in $CDCl_3$.

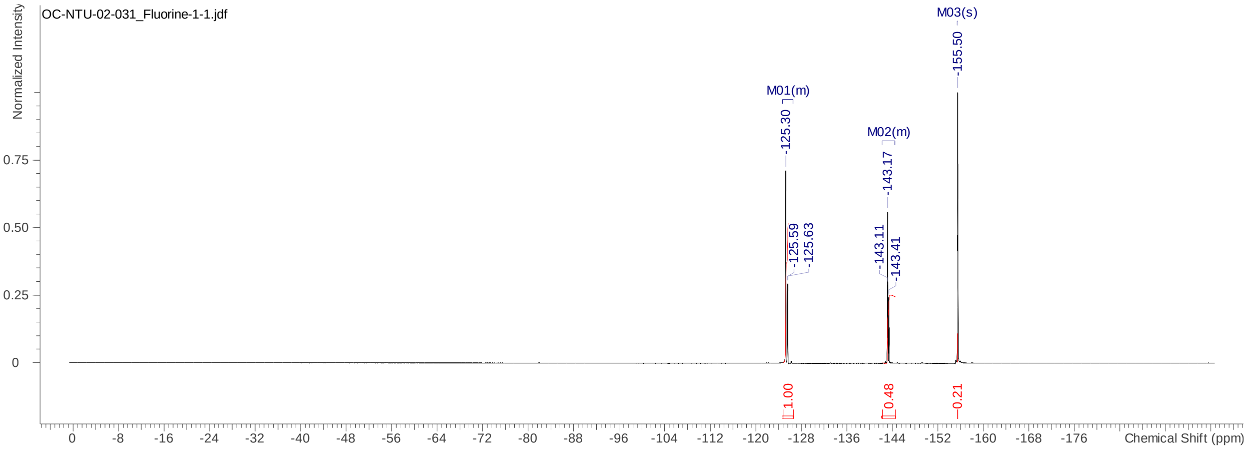

Figure S41. ^{19}F NMR spectrum of **2-f** in $CDCl_3$.

2-g $(3,5\text{-}(CF_3)_2C_6H_3)_3SbCl_2$

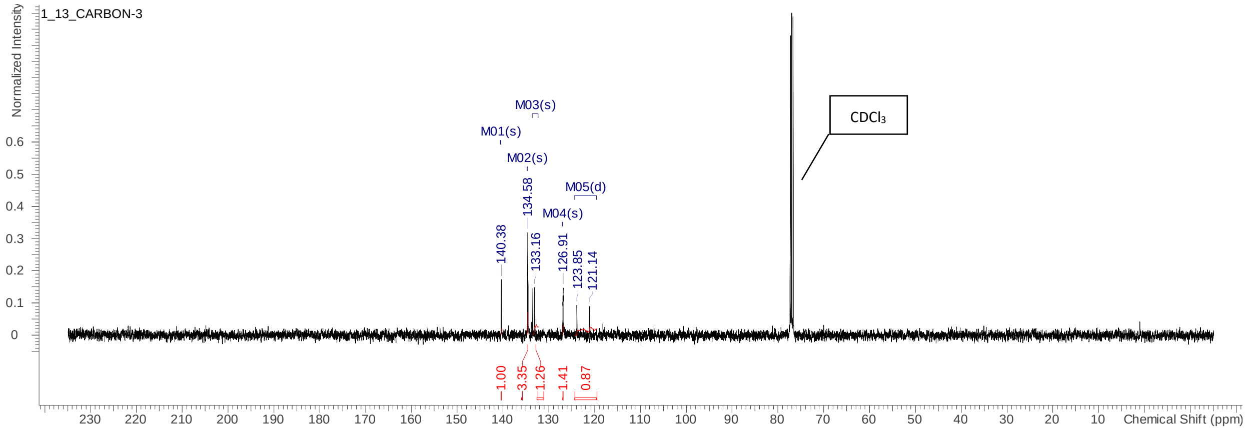

Figure S42. $^{13}C\{^1H\}$ NMR spectrum of **2-g** in $CDCl_3$.

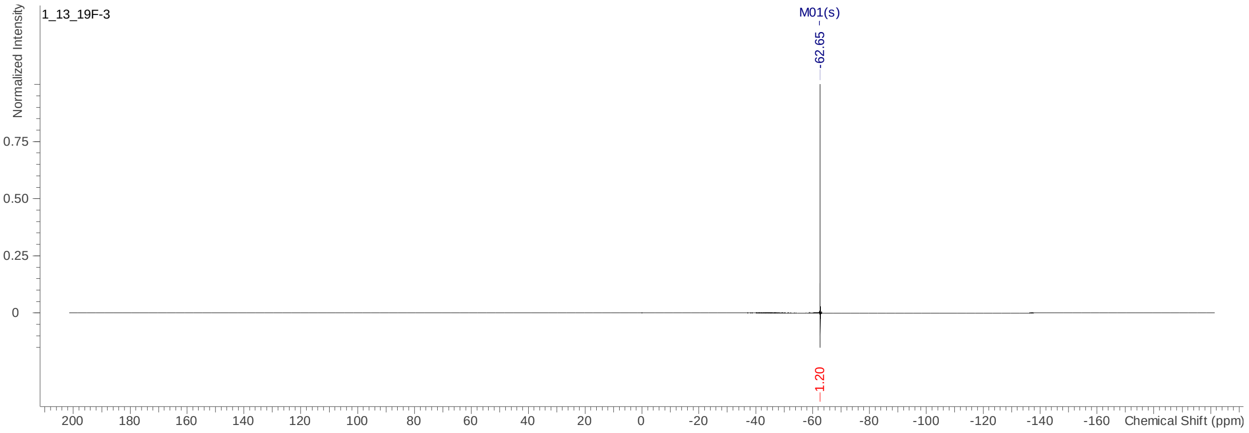

Figure S43. ^{19}F NMR spectrum of **2-g** in $CDCl_3$.

3-a [(Ph)$_3$SbCl][B(C_6F_5)$_4$]

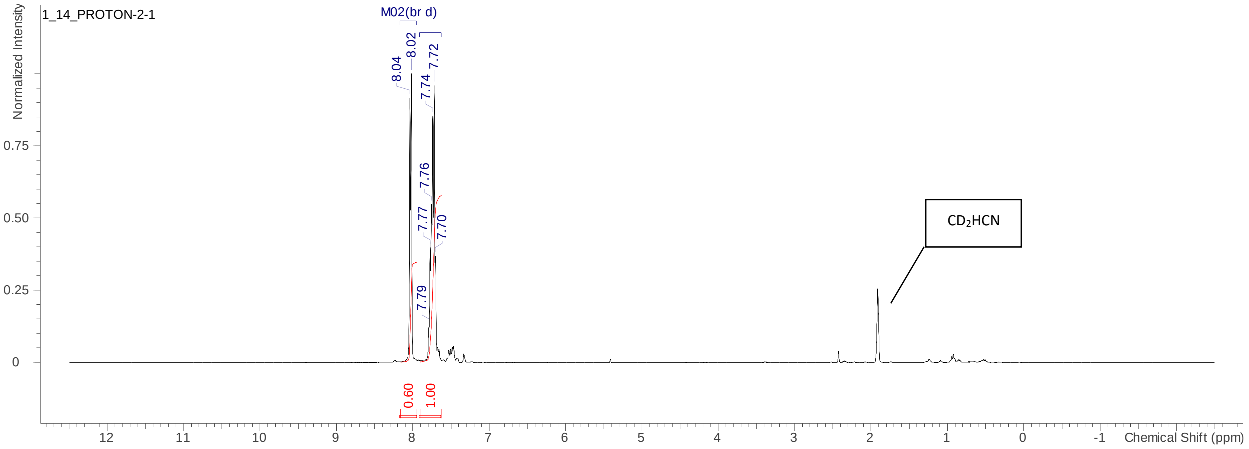

Figure S44. ^{1}H NMR spectrum of **3-a** in CD_3CN.

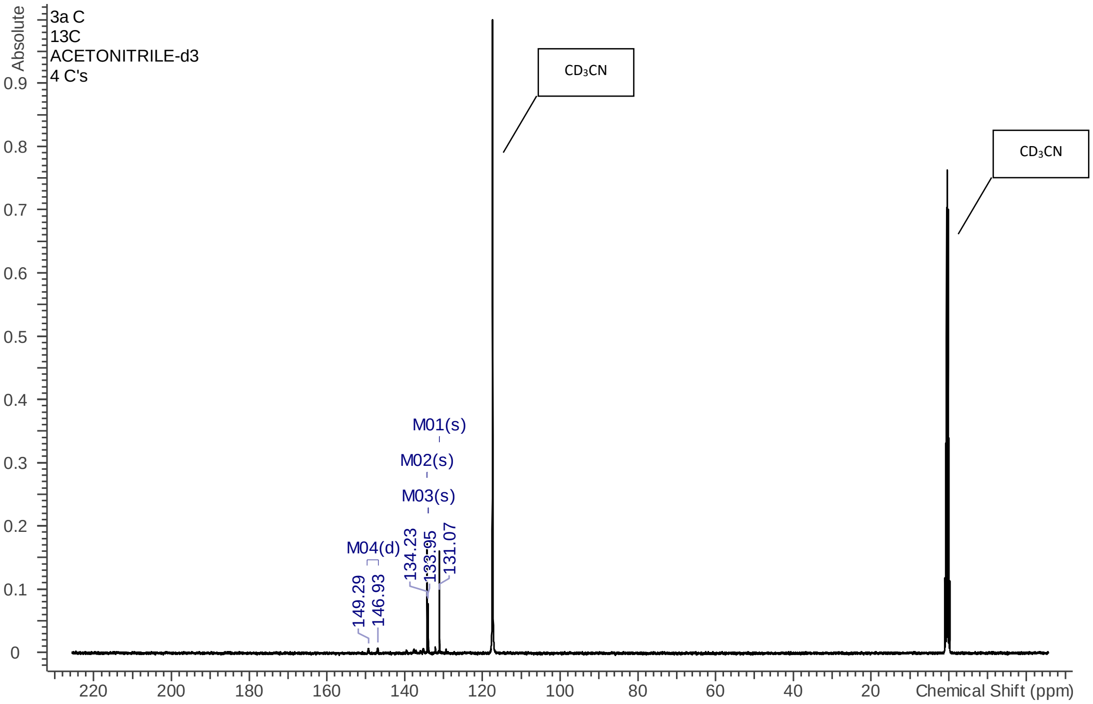

Figure S45. $^{13}C\{^{1}H\}$ NMR spectrum of **3-a** in CD_3CN.

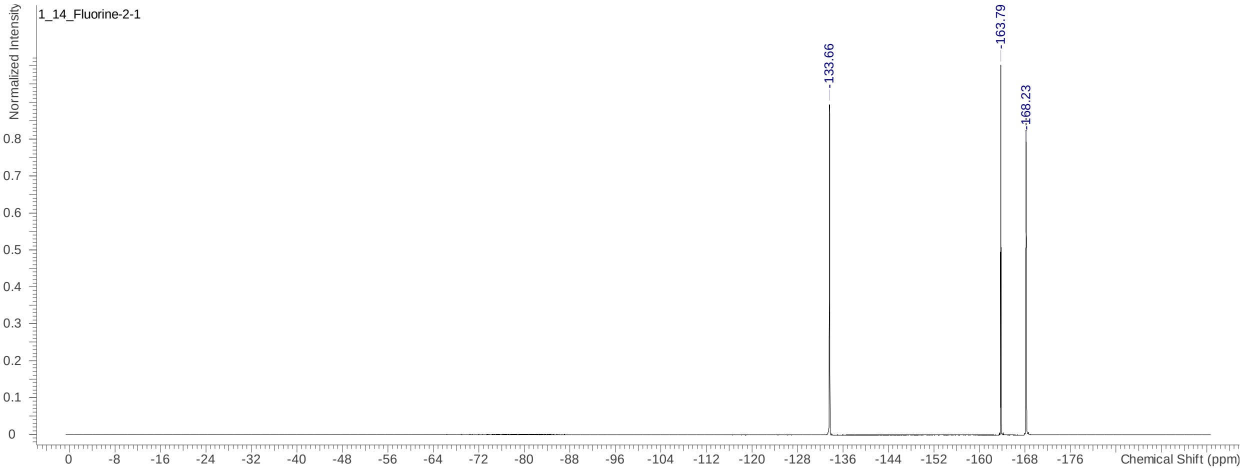

Figure S46. ^{19}F NMR spectrum of **3-a** in CD_3CN.

3-b [$(3\text{-}FC_6H_4)_3SbCl$][$B(C_6F_5)_4$]

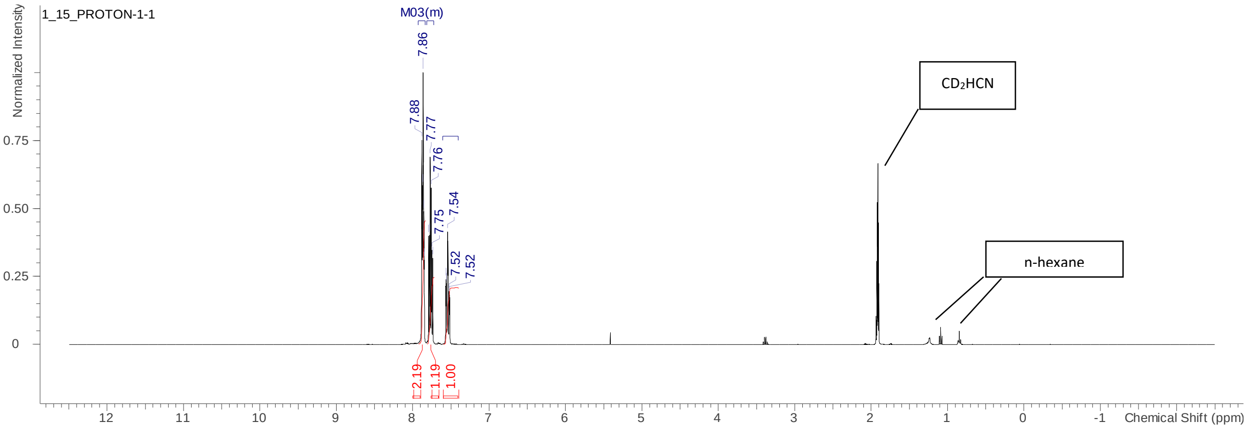

Figure S47. ^{1}H NMR spectrum of **3-b** in CD_3CN.

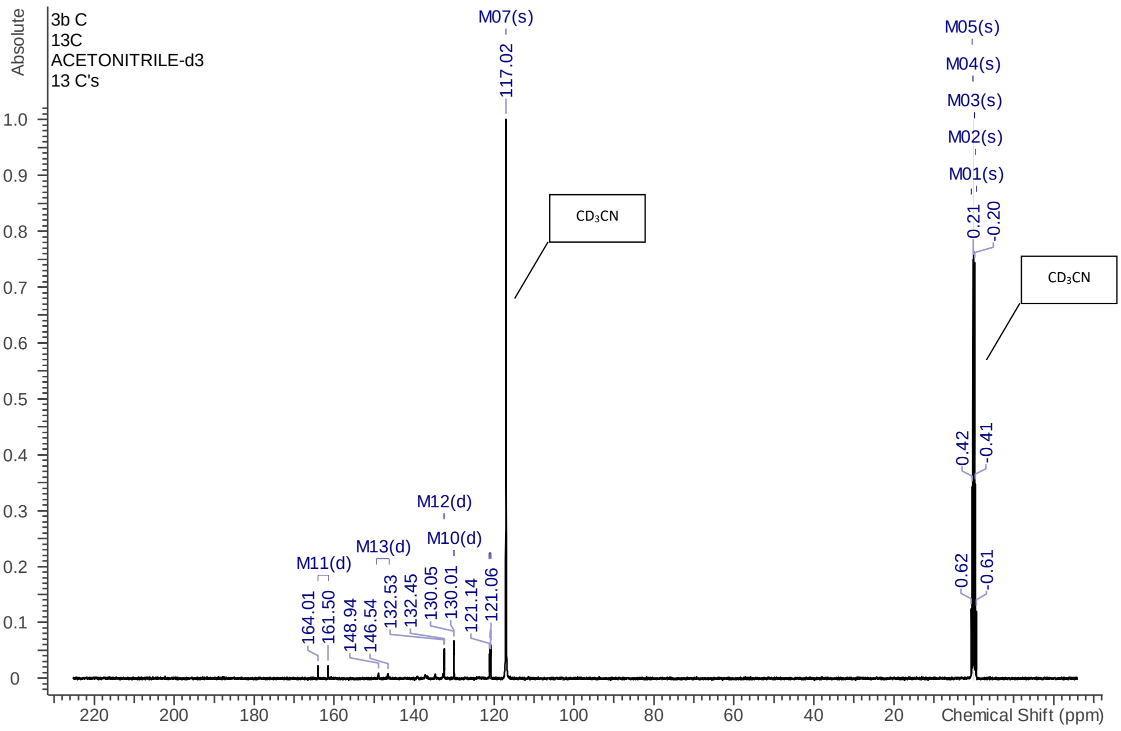

Figure S48. $^{13}C\{^{1}H\}$ NMR spectrum of **3-b** in CD_3CN.

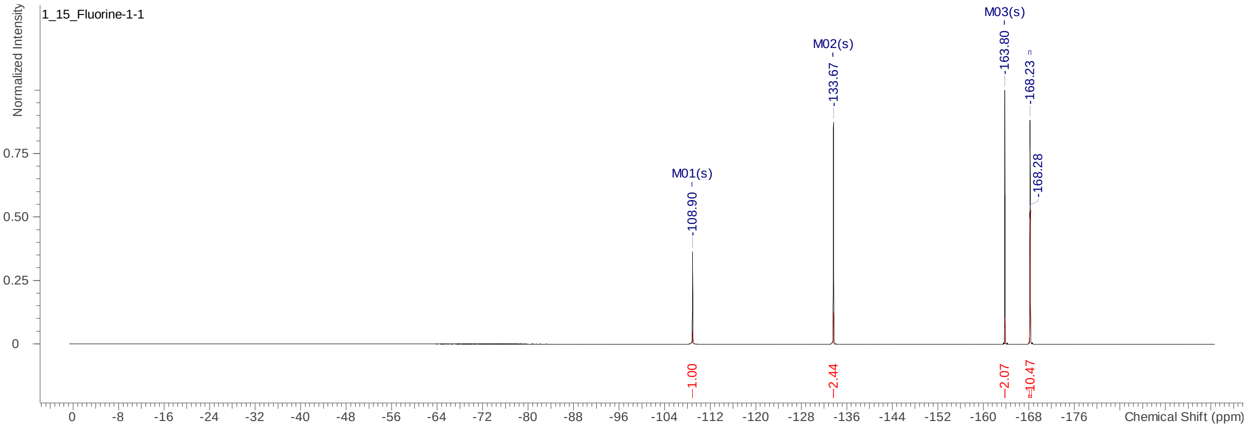

Figure S49. ^{19}F NMR spectrum of **3-b** in CD_3CN.

3-c [$(4\text{-}FC_6H_4)_3SbCl$][$B(C_6F_5)_4$]

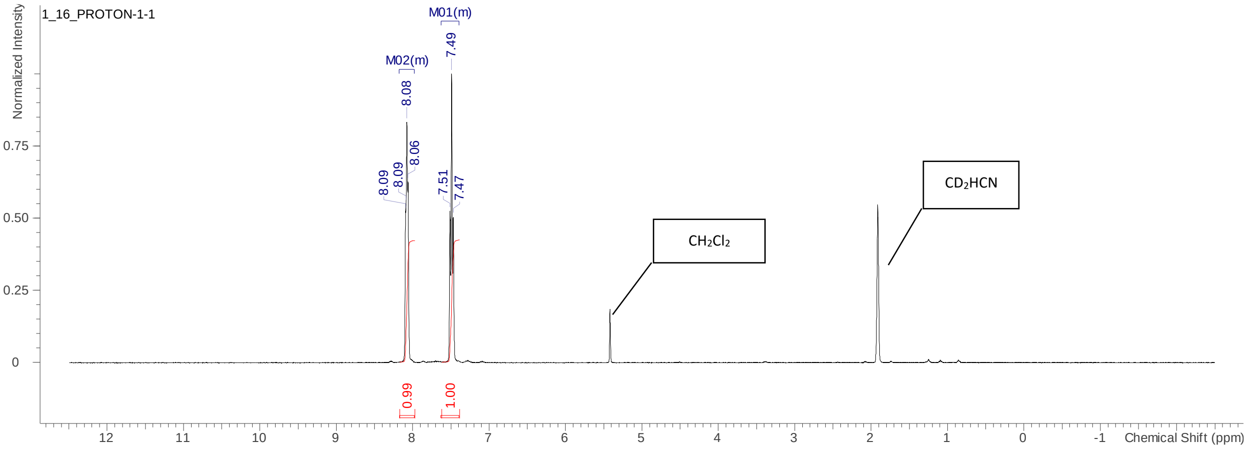

Figure S50. ^{1}H NMR spectrum of **3-c** in CD_3CN.

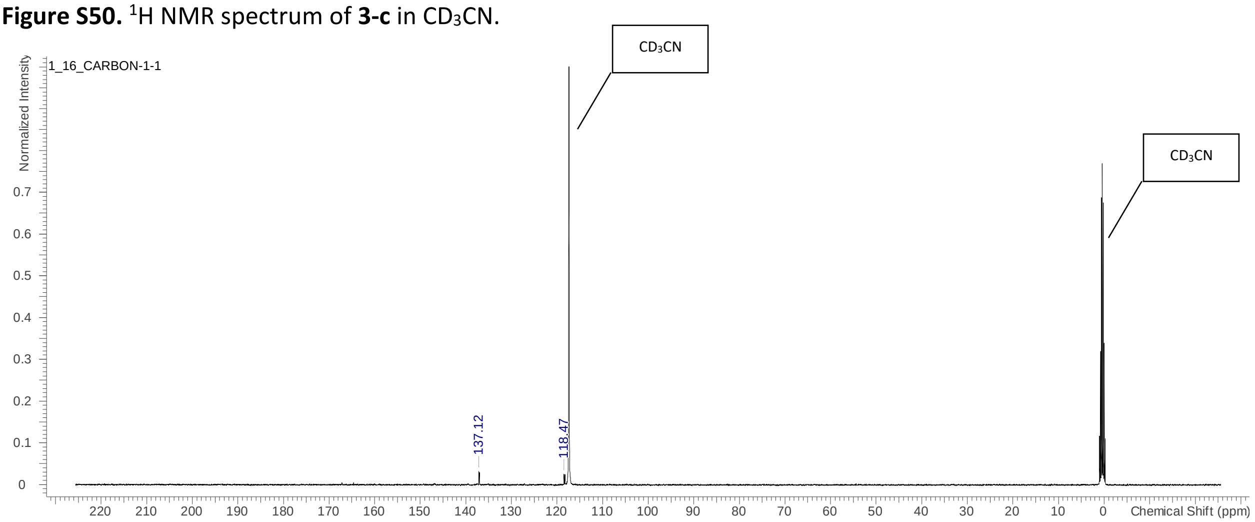

Figure S51. $^{13}C\{^{1}H\}$ NMR spectrum of **3-c** in CD_3CN.

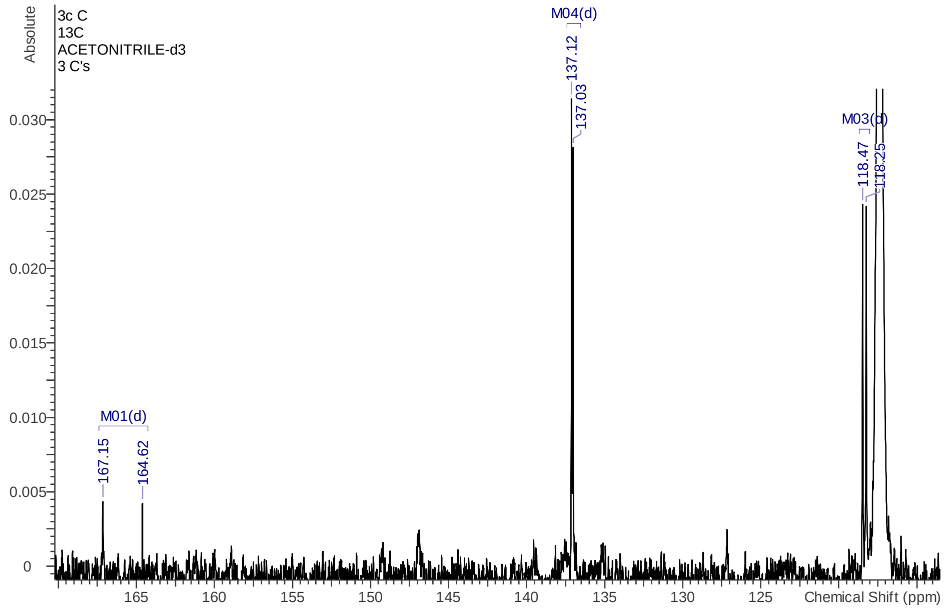

Figure S52. Expanded aromatic region of the $^{13}C\{^{1}H\}$ NMR spectrum of **3-c** in CD_3CN.

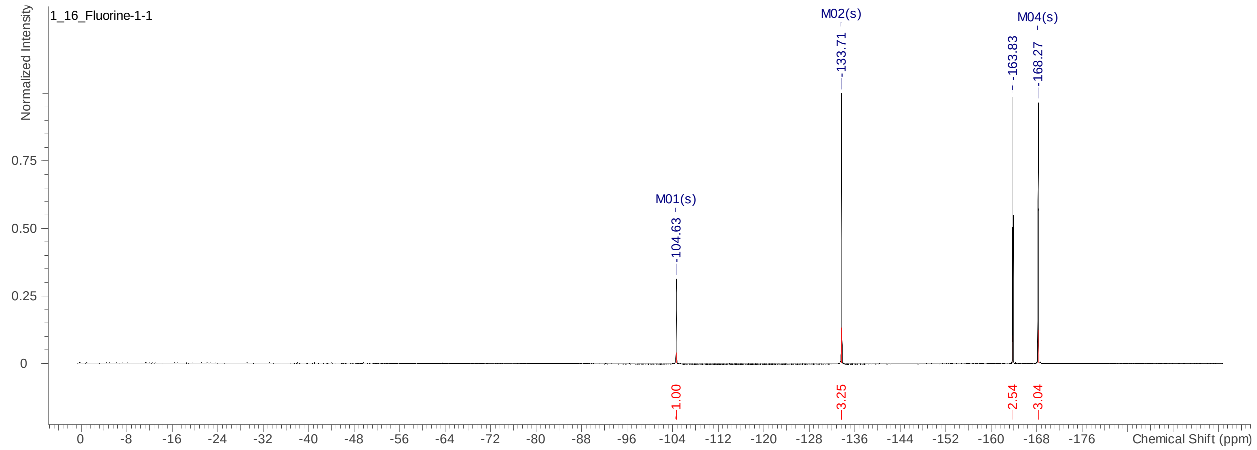

Figure S53. ^{19}F NMR spectrum of **3-c** in CD_3CN.

3-d [(3,5-$F_2C_6H_3$)$_3$SbCl][B(C_6F_5)$_4$]

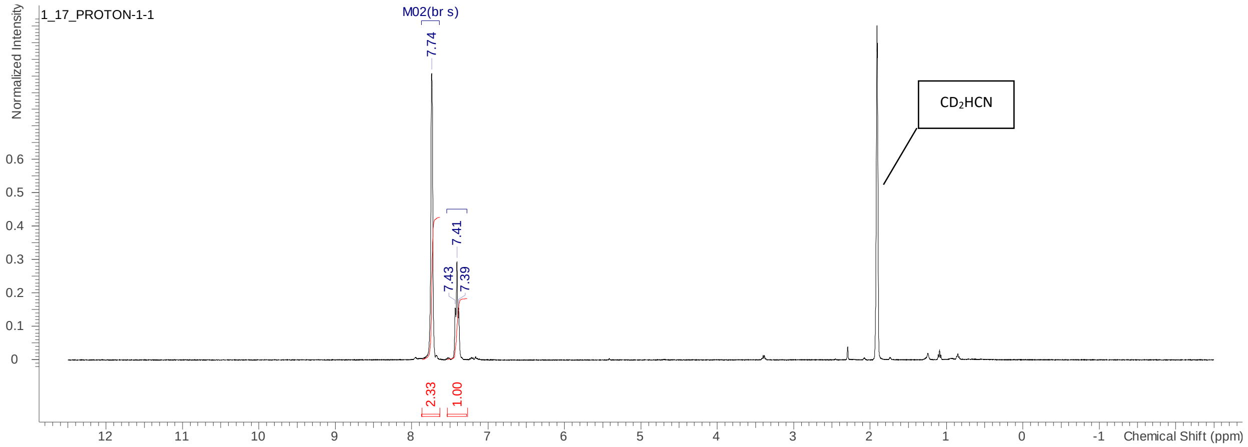

Figure S54. ^{1}H NMR spectrum of **3-d** in CD_3CN.

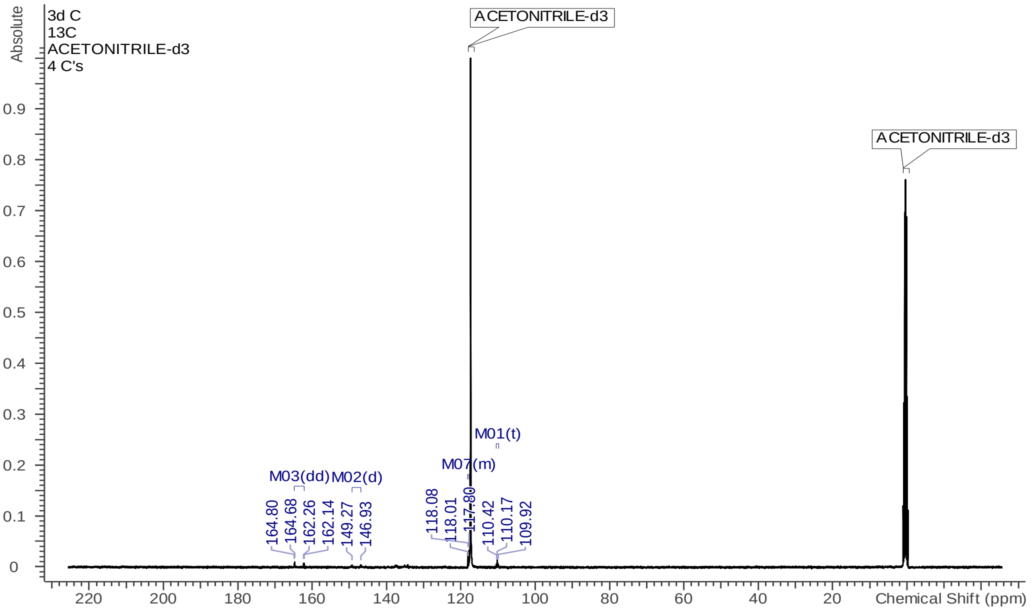

Figure S55. ^{13}C{^{1}H} NMR spectrum of **3-d** in CD_3CN.

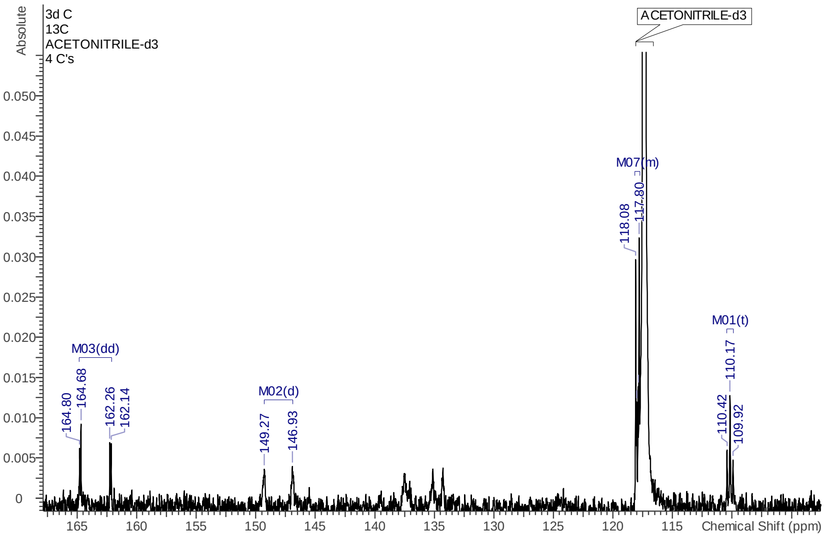

Figure S56. Expanded aromatic region of the $^{13}C\{^{1}H\}$ NMR spectrum of **3-d** in CD_3CN.

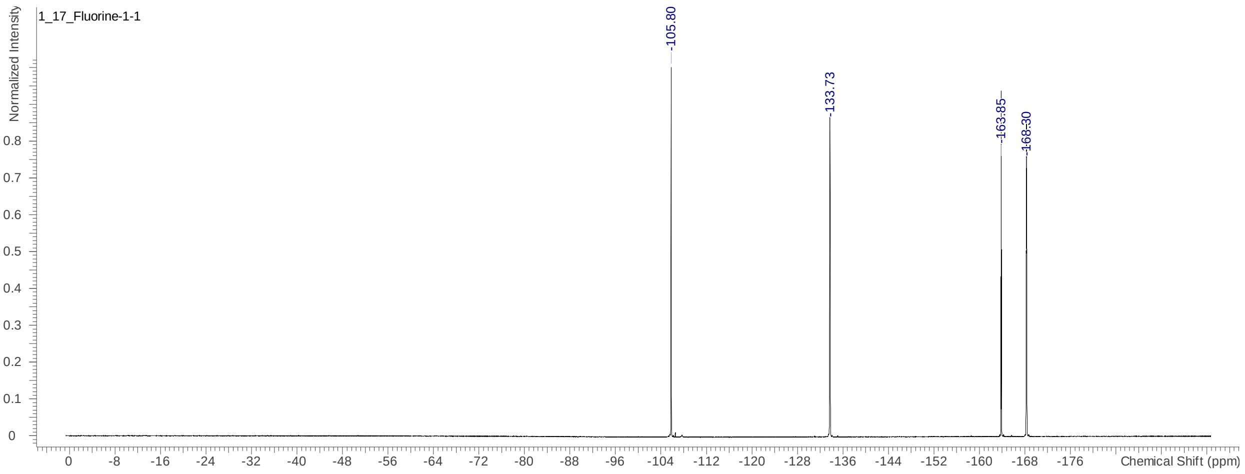

Figure S57. ^{19}F NMR spectrum of **3-d** in CD_3CN.

3-e [(2,4,6-$F_3C_6H_2)_3SbCl][B(C_6F_5)_4$]

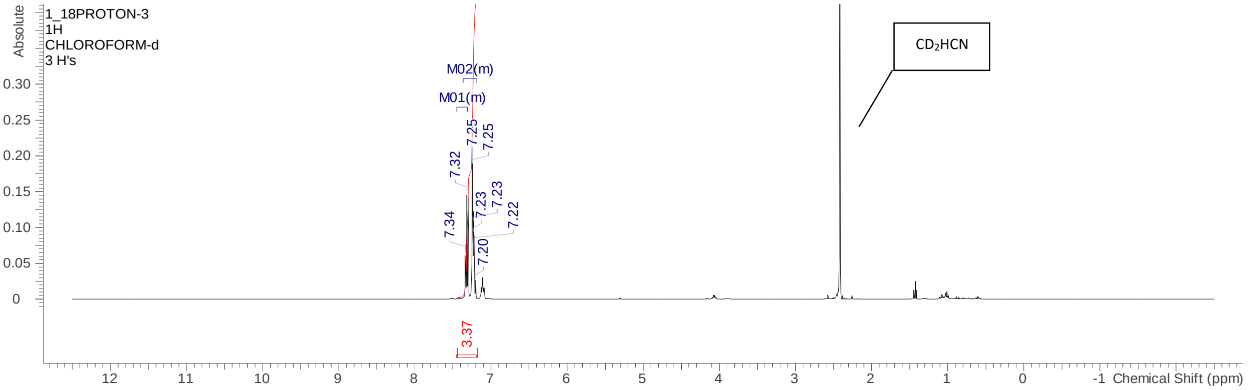

Figure S58. ^{1}H NMR spectrum of **3-e** in CD_3CN.

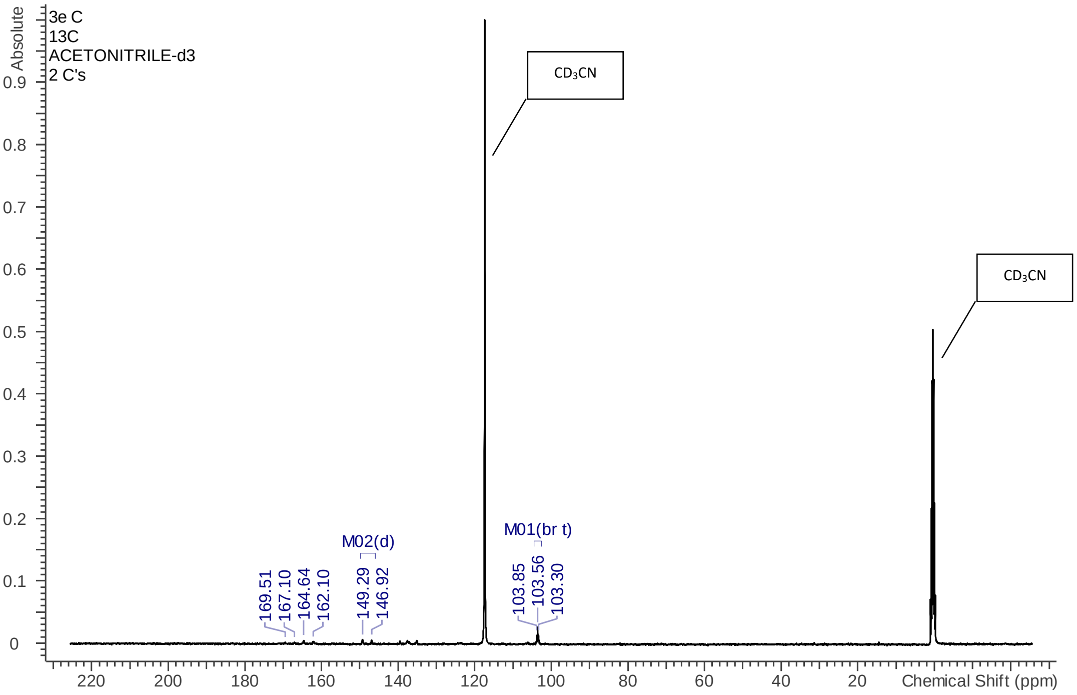

Figure S59. $^{13}C\{^1H\}$ NMR spectrum of **3-e** in CD_3CN.

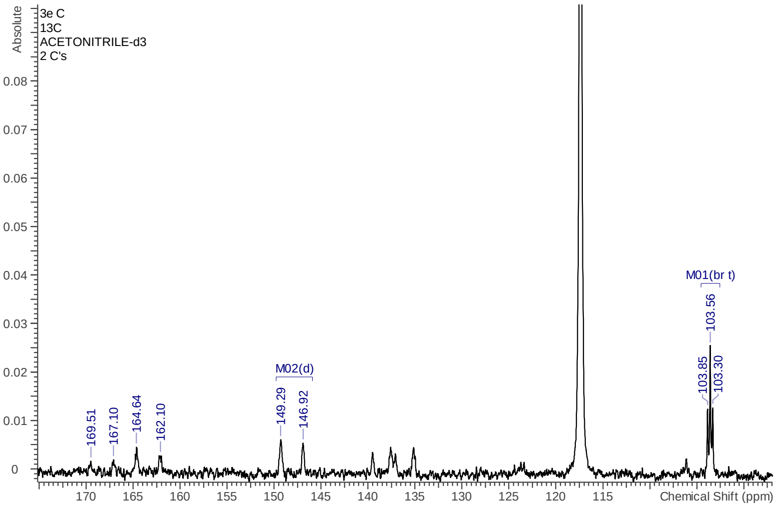

Figure S60. Expanded aromatic region of the $^{13}C\{^{1}H\}$ NMR spectrum of **3-e** in CD_3CN.

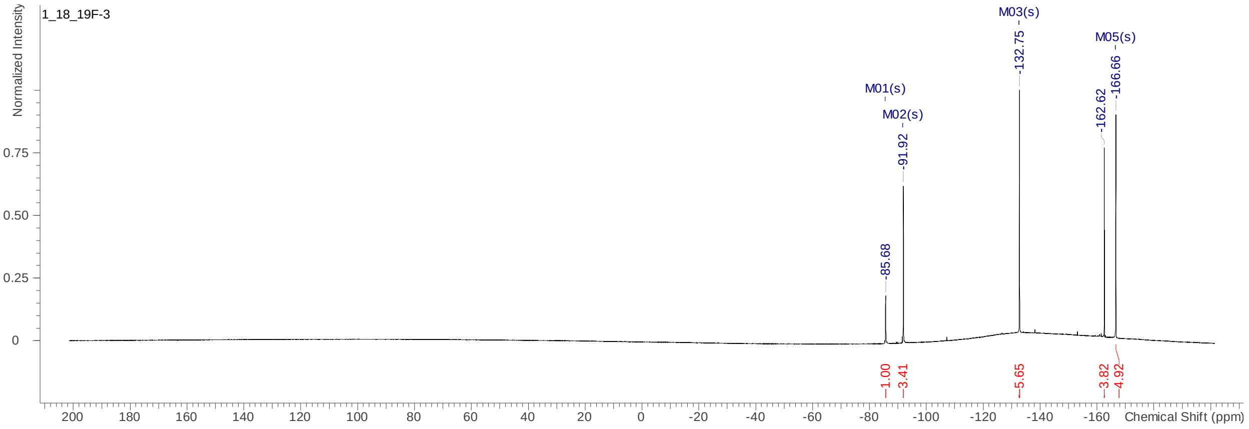

Figure S61. ^{19}F NMR spectrum of **3-e** in CD_3CN.

4-a $[(Ph_3SbCl)_2(\mu\text{-}Cl)][B(C_6F_5)_4]$

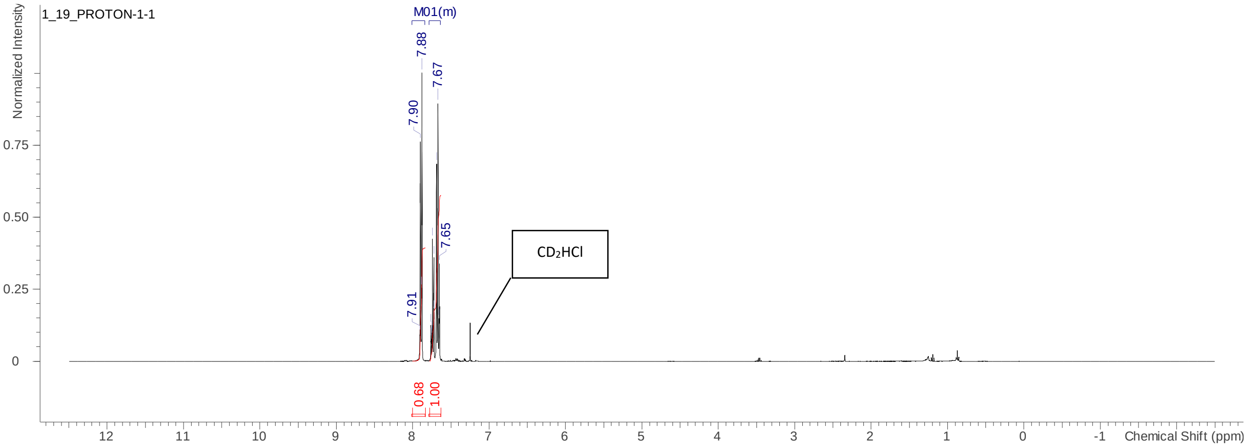

Figure S62. ^{1}H NMR spectrum of **4-a** in $CDCl_3$.

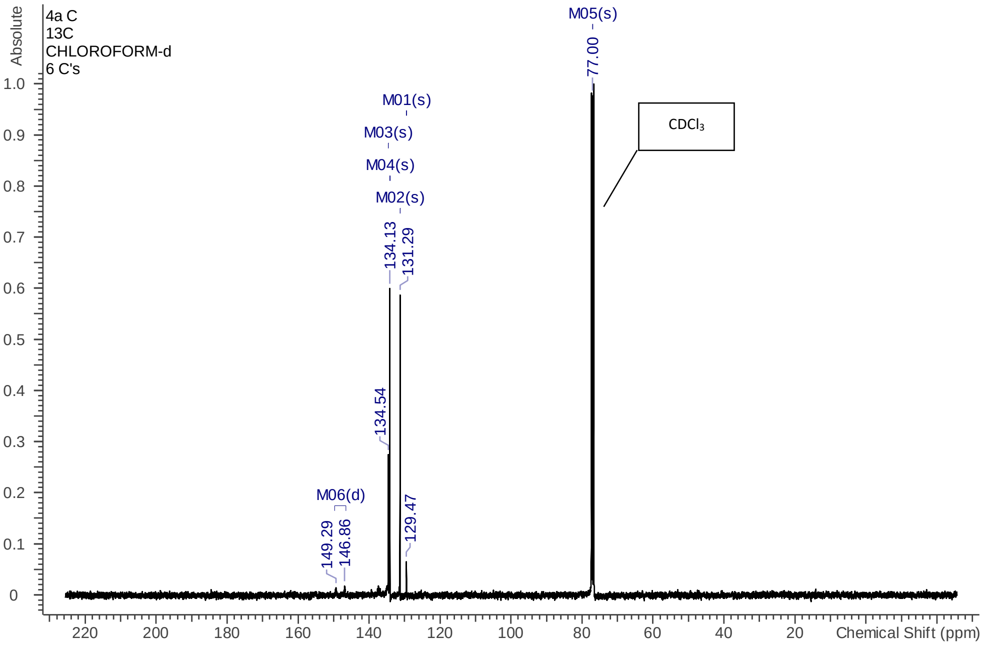

Figure S63. ^{13}C{^{1}H} NMR spectrum of **4-a** in $CDCl_3$.

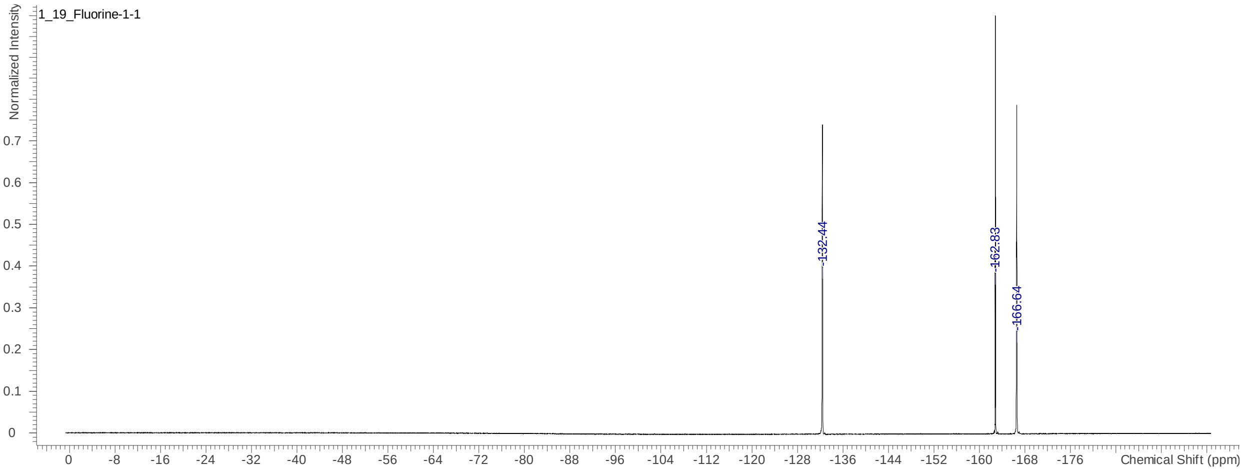

Figure S64. ^{19}F NMR spectrum of **4-a** in $CDCl_3$.

Calculated Frontier Molecular Orbitals

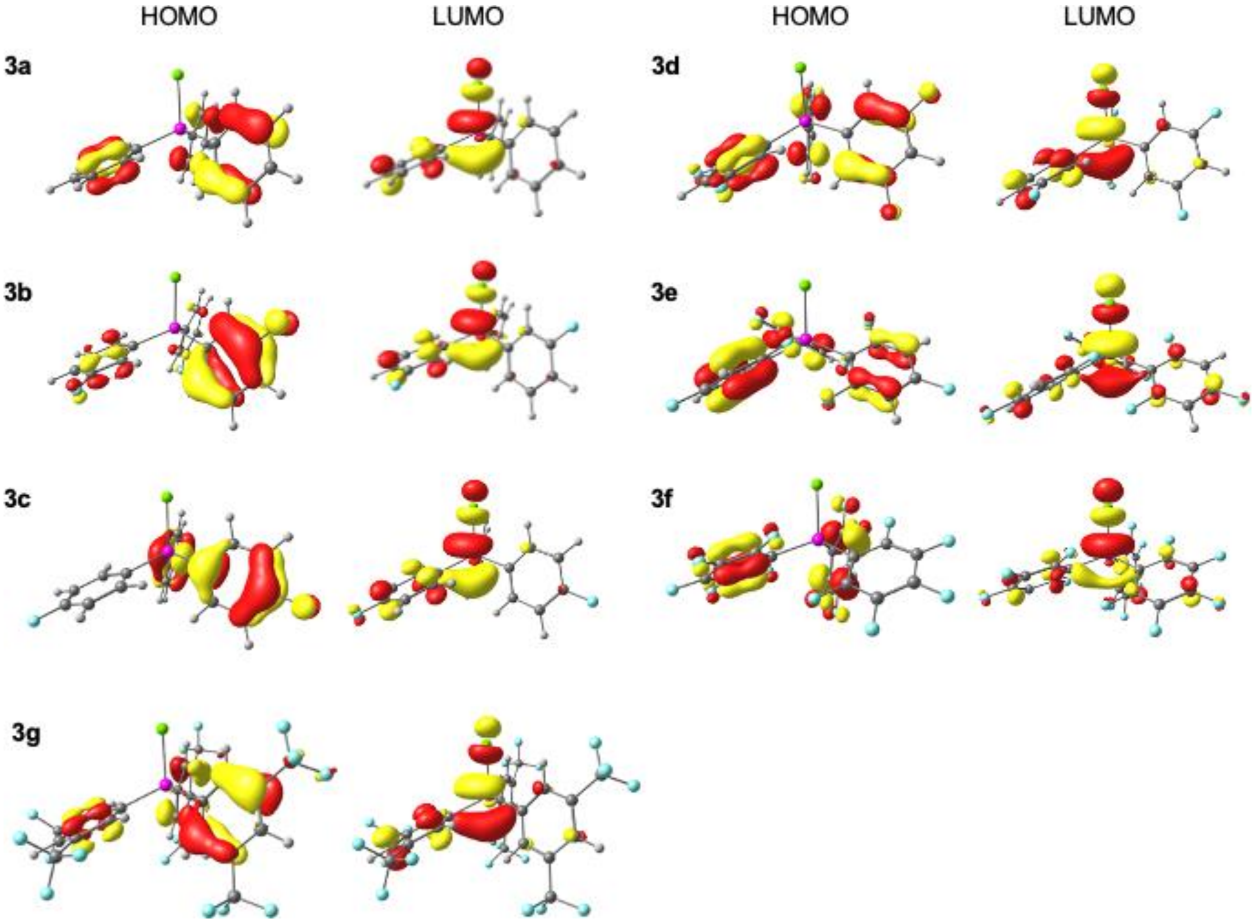

Figure S65. Graphical representations of calculated frontier molecular orbitals for the **3-Ar** series.

Table S2. Calculated FIA and LUMO values for extended **3-Ar** series.

Compound	Sb-Cl (Å)	FIA (kJ/mol)	LUMO (eV)
3-a	2.2821(14)	566	–4.64
3-b	2.2924(11)	595	–4.97
3-c	2.2757(9)	591	–4.82
3-d	2.2604(19), 2.2608(16)	623	–5.28
3-e	2.2532(19)	597	–5.15
3-f	–	652	–5.65
3-g	–	665	–5.64
$[SiEt_3(tol)]^+$	–	706	–5.25

Computational investigation of alternative reaction pathways in the reduction of Ph_3SbCl^+

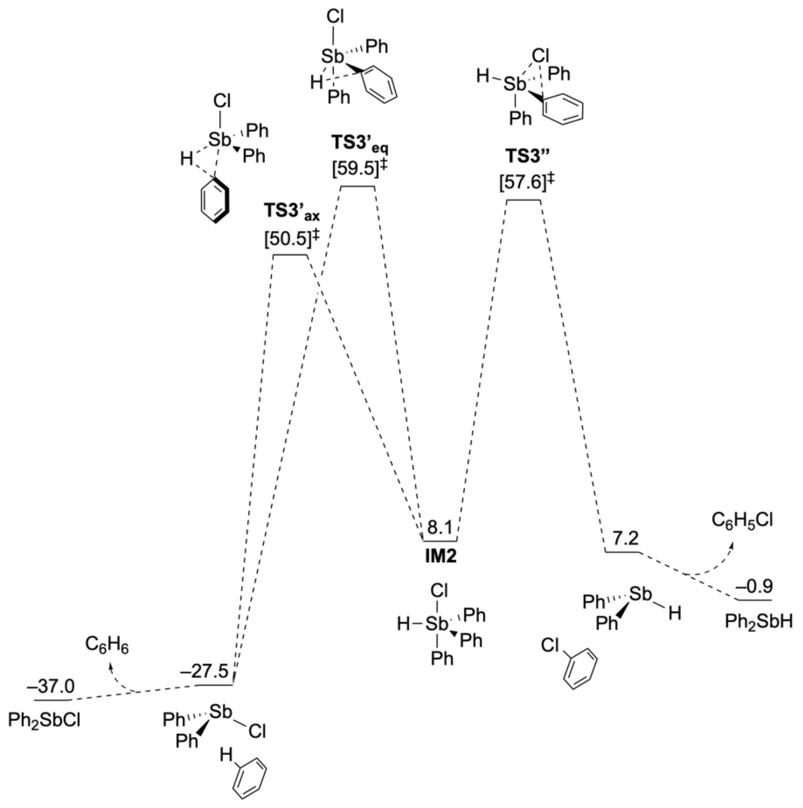

Figure S66. Calculated reaction profile for the reduction of Ph_3SbCl^+ by Et_3SiH via elimination of benzene or chlorobenzene. Gibbs Free Energies (in kcal mol^{-1}) relative to starting materials Ph_3SbCl^+ and 2 Et_3SiH. All energies are calculated at the M06–2X(D3)/def2-QZVPP//M06-2X(D3)/def2-SVP level of theory corrected for CH_2Cl_2 solvent.

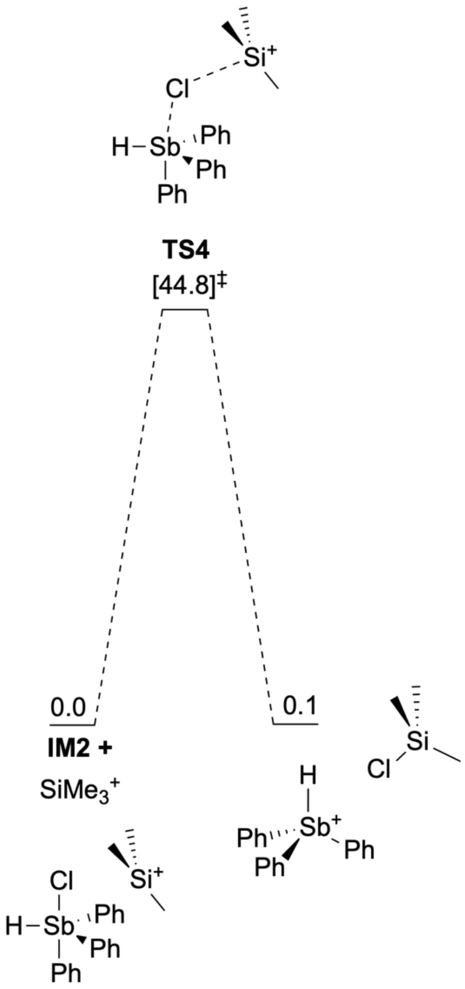

Figure S67. Calculated reaction profile for the reduction of Ph_3SbCl^+ by Et_3SiH via initial abstraction of chloride by $SiMe_3^+$. Gibbs Free Energies (in kcal mol^{-1}) relative to starting materials Ph_3SbCl^+ and 2 Et_3SiH. All energies are calculated at the M06–2X(D3)/def2-QZVPP//M06-2X(D3)/def2-SVP level of theory corrected for CH_2Cl_2 solvent.

References

(1) Lambert, J. B.; Zhang, S.; Ciro, S. M. Silyl Cations in the Solid and in Solution. *Organometallics* **1994**, *13* (6), 2430–2443. https://doi.org/10.1021/om00018a041.

(2) Lancaster, S. Alkylation of boron trifluoride with pentafluorophenyl Grignard reagent; Tris(pentafluorophenyl)boron; borane. *ChemSpider Synthetic Pages*. https://doi.org/10.1039/SP215.

(3) Martin, E.; Hughes, D. L.; Lancaster, S. J. The Composition and Structure of Lithium Tetrakis(Pentafluorophenyl)Borate Diethyletherate. *Inorganica Chim Acta* **2010**, *363* (1), 275–278. https://doi.org/10.1016/j.ica.2009.09.013.

(4) Connelly, S. J.; Kaminsky, W.; Heinekey, D. M. Structure and Solution Reactivity of (Triethylsilylium)Triethylsilane Cations. *Organometallics* **2013**, *32* (24), 7478–7481. https://doi.org/10.1021/om400970j.

(5) Fild, M.; Glemser, O.; Christoph, G. Synthese von Tris-Pentafluorphenylarsin, -Stibin Und -Phosphin Sowie von Trimethyl-Pentafluorphenylsilan. *Angewandte Chemie* **1964**, *76* (23), 953–953. https://doi.org/10.1002/ANGE.19640762304.

(6) de Ketelaere, R. F.; Delbeke, F. T.; van der Kelen, G. P. Organo Group VB Chemistry III. Synthesis and NMR Spectra of Some Tertiary Substituted Arylstibines and Arylbismuthines. *Journal of Organometallic Chemistry*. 1971, pp 365–368. https://doi.org/http://dx.doi.org/10.1016/S0022-328X(00)87520-8.

(7) Yasuike, S.; Nakata, K.; Qin, W.; Matsumura, M.; Kakusawa, N.; Kurita, J. Synthesis of Arylboronates by Boron-Induced Ipso-Deantimonation of Triarylstibanes with Boron Trihalides and Its Application in One-Pot Two-Step Transmetallation/Cross-Coupling Reactions. *J Organomet Chem* **2015**, *788*, 9–16. https://doi.org/10.1016/J.JORGANCHEM.2015.04.017.

(8) Rahman, A. F. M. M.; Murafuji, T.; Ishibashi, M.; Miyoshi, Y.; Sugihara, Y. Chlorination of P-Substituted Triarylpnictogens by Sulfuryl Chloride: Difference in the Reactivity and Spectroscopic Characteristics between Bismuth and Antimony. *J Organomet Chem* **2005**, *690* (19), 4280–4284. https://doi.org/10.1016/J.JORGANCHEM.2005.06.040.
